# Genome-wide identification of evolutionarily conserved Small Heat-Shock and eight other proteins bearing α-crystallin domain-like in kinetoplastid protists

**DOI:** 10.1371/journal.pone.0206012

**Published:** 2018-10-22

**Authors:** André G. Costa-Martins, Luciana Lima, João Marcelo P. Alves, Myrna G. Serrano, Gregory A. Buck, Erney P. Camargo, Marta M. G. Teixeira

**Affiliations:** 1 Department of Parasitology, Institute of Biomedical Sciences, University of São Paulo, São Paulo, SP, Brazil; 2 INCT-EpiAmO–Instituto Nacional de Epidemiologia na Amazônia Ocidental, Porto Velho, RO, Brazil; 3 Department of Microbiology and Immunology, Virginia Commonwealth University, Richmond, VA, United States of America; University of Toronto, CANADA

## Abstract

Small Heat-Shock Proteins (sHSPs) and other proteins bearing alpha-crystallin domains (ACD) participate in defense against heat and oxidative stress and play important roles in cell cycle, cytoskeleton dynamics, and immunological and pathological mechanisms in eukaryotes. However, little is known about these proteins in early-diverging lineages of protists such as the kinetoplastids. Here, ACD-like proteins (ACDp) were investigated in genomes of 61 species of 12 kinetoplastid genera, including *Trypanosoma* spp. (23 species of mammals, reptiles and frogs), *Leishmania* spp. (mammals and lizards), trypanosomatids of insects, *Phytomonas* spp. of plants, and bodonids. Comparison of ACDps based on domain architecture, predicted tertiary structure, phylogeny and genome organization reveals a kinetoplastid evolutionarily conserved repertoire, which diversified prior to trypanosomatid adaptation to parasitic life. We identified 9 ACDp orthologs classified in 8 families of *Try*ACD: four previously recognized (HSP20, *Try*p23A, *Try*p23B and ATOM69), and four characterized for the first time in kinetoplastids (*Try*ACDP, *Try*SGT1, *Try*DYX1C1 and *Try*NudC). A single copy of each ortholog was identified in each genome alongside *Try*NudC1/*Try*pNudC2 homologs and, overall, ACDPs were under strong selection pressures at main phylogenetic lineages. Transcripts of all ACDPs were identified across the life stages of *T. cruzi*, *T. brucei* and *Leishmania* spp., but proteomic profiles suggested that most ACDPs may be species- and stage-regulated. Our findings establish the basis for functional studies, and provided evolutionary and structural support for an underestimated repertoire of ACDps in the kinetoplastids.

## Introduction

Trypanosomatids (Euglenozoa, Kinetoplastea, Trypanosomatidae) are successful and widespread protists that branched very early from other eukaryotes. They are obligate parasites of all classes of vertebrates to which they are transmitted by hematophagous arthropods and leeches. Some trypanosomatids can be pathogenic to humans (*Trypanosoma cruzi*, *T*. *brucei* ssp. and *Leishmania* spp.), livestock (*T*. *b*. *brucei*, *T*. *congolense* and *T*. *vivax*) and plants (*Phytomonas*), but the majority are apparently harmless [1–3]. They exhibit remarkably diverse life cycles, are monoxenous of insects, and dixenous species rely on different strategies to infect, proliferate, differentiate, be transmitted by their vectors, and evade host defenses. Most trypanosomes proliferate in the bloodstream. However, *T*. *cruzi* and other species of the subgenus *Schizotrypanum* develop as amastigote and trypomastigote forms in the cytosol of mammalian cells, *Leishmania* spp. multiply as amastigotes within macrophage compartments in vertebrate hosts. Most trypanosomatids develop exclusively in the digestive tract of their vectors and are transmitted among insects by coprofagia and to vertebrates by contamination via the vector bit wound or through exposure to mucosal surfaces, with infective forms present in vector feces (e.g., *T*. *cruzi* in triatomine bugs). In contrast, infective forms of *T*. *brucei* and *T*. *rangeli* develop respectively in the salivary glands of tsetse flies and triatomine bugs, and although *Leishmania* spp. developed only in sand fly guts, these three species are inoculated into vertebrate hosts with saliva during vector feeding [1, 2, 4–6].

The kinetoplastids live in different environments and consequently have to deal with different stress challenges either in the aquatic habitats of the bodonids, and throughout successive life cycle changes in their vertebrate and invertebrate hosts, and various intra-host niches. The success of kinetoplastids in their complex and variable life-cycles relies on their capacity to survive often stressful conditions. Commensal bodonids have to face drastic changes in their free-living lifestyle in aquatic habitats challenged by often very rapid changes in temperature, humidity, salinity, pH, availability of oxygen, and other environmental stresses. Species of trypanosomatids also survive in a wide range of temperatures, nutrient starvation, pH and oxidative stress during their transition from invertebrate vectors to vertebrates or plant hosts, where they reside in different niches [1–3]. Stress response is characterized by the expression of many proteins, particularly the heat shock proteins (HSPs), whose main function is to act as molecular chaperones, the key regulators of the stress response formed by many proteins interacting with each other and their ligands. The networks of HSPs and other molecules play vital roles in coordinating the capacity of the cells to deal with stress and survive in their habitually rapidly changing environments [7–9].

Several heat-shock protein (HSP) chaperones and co-chaperones have been described in kinetoplastids and classified according to molecular mass as sHSP (small HSP), p23, Hsp40, Hsp60, Hsp70, Hsp90 and Hsp110 [10–15]. The sHSP family consists of heterogeneous members characterized by a molecular signature, the conserved α-crystallin domain (ACD), flanked by a variable N and C-terminal. The α-crystallin domain (ACD) is an ancient and conserved domain ubiquitous in all kingdoms. It is constituted of β-strands forming a compact antiparallel β-sandwich highly similar in three-dimensional folding structure [16–19].

The repertoires of ACD proteins (ACDp) in eukaryotes comprise many families, including the sHSPs and p23, whose functions have been extensively investigated. A large number of functional studies of ACDps in yeasts, plants and a range of metazoans have revealed complex and vital functions as molecular chaperones and in diverse cellular pathways such as cell proliferation, differentiation, migration and signalization, cytoskeleton and cilia dynamics, immunological responses, and pathogen resistance [20–25].

A few ACDp families have been identified in a few species of protists. The ubiquitous sHSP family is comprised of five copies in *Toxoplasma* and six copies in *Plasmodium* but vary broadly with up to dozens of copies in plants and animals (11 in humans) [13,26,27]. In kinetoplastids, a single copy of sHSP (Hsp20) was identified in the genomes of *T*. *brucei*, *T*. *cruzi* and *Leishmania* spp., and its expression was shown to be enhanced by heat and the oxidative stresses of vertebrate infection [13,28,29]. Genetic and functional studies disclosed two p23 proteins (p23A and p23B) in *Leishmania* spp. with homologs in *T*. *cruzi* and *T*. *brucei* [30,31]. Recently, ATOM69, an outer membrane protein translocase of the complex machinery responsible for mitochondrial protein import containing an ACD-like (Hsp20-like) domain was reported in kinetoplastids [32,33]. Whether other ACD-like protein families are present in these protists in addition to these three proteins remains to be investigated.

A better knowledge of ACDp repertoires and their evolution in a comprehensive set of kinetoplastids representing overall evolutionary diversity of these flagellates would provide relevant insights regarding the biology of these organisms and their strategies for facing hostile environments in their vertebrate and invertebrate hosts. Comparative genomic analysis that includes both closely and distantly related species has catalyzed the rapid progress of evolutionary studies of proteins that play important roles in physiological processes of these parasites and in their adaptation to different hosts and environments through their evolutionary histories.

In the present study, our main goal was to apply bioinformatic approaches to systematically investigate the genomic repertoire of ACDps in a highly comprehensive set of parasitic trypanosomatids (61 species of 12 genera), characterize their transcriptomic and proteomic profiles, and identify evidence of diversifying selection pressure on these proteins. We examined representatives of all main phylogenetic lineages of monoxenous and dixenous kinetoplastid species that are infective to vertebrate hosts of different classes, are transmitted by different vectors, and differ in extra- and intra-cellular niches, and in many characteristics of their host-parasite-vector interactions. In addition to obligate parasitic trypanosomatids, we trace the evolution of ACDps to kinetoplastid members of their closest relative free-living bodonids.

## Material and methods

### Kinetoplastid genomes examined in this study

Genomes of the organisms examined in this study were *Trypanosoma* spp. of mammals: *T*. *b*. *brucei*, *T*. *b*. *gambiense*, *T*. *evansi*, *T*. *vivax*, *T*. *congolense*, *T*. *cruzi CL Brener*, *T*. *cruzi* G, *T*. *c marinkellei*, *T*. *dionisii*, *T*. *erneyi*, *T*. *rangeli*, *T*. *conorhini*, *T*. *lewisi*, *T*. *wauwau*, *T*. *noyesi*, *T*. *livingstonei*, *T*. *cyclops* and *T*. *theileri*. *Trypanosoma* spp. of cold-blooded hosts: *T*. *grayi* (crocodiles), *T*. *ralphi* (caiman)*; Trypanosoma* sp. TCC878 (lyzard)*; Trypanosoma* sp. TCC339 (toad), *Trypanosoma* sp. TCC1307 (frog), *Trypanosoma* sp. TCC2186 (frog). Species of *Leishmaniinae* included in the analyses were from mammals, *L*. *braziliensis*, *L*. *panamensis*, *L*. *aethiopica*, *L*. *amazonensis*), *L*. *arabica*, *L*. *donovani*, *L*. *gerbilli*, *L*. *infantum*, *L*. *major*, *L*. *mexicana*, *L*. *tropica*, *L*. *turanica*, *L*. *enrietti* and *L*. *martiniquensis*; and *L*. *tarentolae* of lizard, *Endotrypanum monterogeii* and *E*. *schaudinni* parasites of sloths and sand flies; and the insect parasites *Zelonia costaricensis*, *Zelonia* sp., *Crithidia acanthocephali*, *C*. *thermophila*, *C*. *fasciculata* and *Leptomonas pyrrhocoris*. Other monoxenous trypanosomatids included in the analyses were: *Herpetomonas wanderley*, *H*. *muscarum*, *Angomonas deanei*, *A*. *desouzai*, *Strigomonas culicis*, *S*. *galati*, and *S*. *oncopelti*. Plant trypanosomatids: *Phytomonas serpens*, *P*. *dolleti*, *Phytomonas* sp. HART1, *Phytomonas* sp. EM1 and *Phytomonas* Jma. Free-living kinetoplastid: *Bodo saltans*, *Bodo* sp. and *Parabodo caudatus*.

Reference codes of each organism and respective host species and geographical origin are shown in supplementary data (S1 Table). TCC numbers refer to codes of trypanosomatids cryopreserved at the Trypanosomatid Culture Collection of the University of São Paulo (TCC-USP). Flagellates were cultivated in LIT medium supplemented with 2–5% FBS (Fetal Bovine Serum), and genomes were sequenced using shotgun methodology [34] or MiSeq Illumina platform assembled using Newbler version 2.9.

### Genomic searches, annotation, and sequence alignments

All proteins analyzed here share ACD domain. Due to low levels of similarity in the whole protein sequence, searches were conducted with RPS-BLAST using a maximum expect value (E-value) threshold of 1e-5 against genome open reading frames obtained using the Getorf program of EMBOSS package v6.6.0.0 with standard parameters and Position-specific Score Matrices (PSSMs) for ACD in the Conserved Domain Databases (CDD -NCBI http://www.ncbi.nlm.nih.gov/cdd). PSSMs for ACDps including description and accession numbers are shown in S2 Table. Retrieved sequences were annotated according to domain architectures and similarity searches (BLASTp and PSI-BLAST) against non-redundant protein database (NR-NCBI). A minimum E-value threshold of 1e-5 was used in BLASTp, while for PSI-BLAST searches initial E-value thresholds were 1e-5 and 1e-3; only subjects showing at least 50% query coverage were included in the consecutive rounds. The searches were interrupted in the fourth round. Sequences obtained were deposited in GenBank and descriptions are available in S2 Table.

Analyses of ACD, signature (sequence Logo) and alignment of the orthologs found in trypanosomatids were conducted using GLAM2 (Gapped Local Alignment of Motifs) included in MEME software suite version 3.5.4 [35]. Average amino acid identity between ACDp was calculated with MEGA software v6.0 using the P-distance method and pairwise deletion for missing data. To provide a visual representation of ACDps distance matrix, we performed multidimensional scaling (MDS) plot with two dimensions (2D) using the Bios2mds package of the R statistical language environment for statistical computing [36].

### Prediction of tertiary structure

The tertiary protein structures of ACD from all trypanosomatid ACDp were constructed using the SWISS-MODEL homology-modeling server [37]. ACDp sequences obtained herein were used as query and best-scored templates used to build sequence models. Models were visualized and aligned using the SwissPdbViewer v4.1 program [38]. The access numbers from crystal structures used as templates in homology-modeling are shown in S2 Table.

### Phylogenetic and positive selection analyses of ACDp orthologs

The ACDp orthologs were aligned using MUSCLE v3.8.31 [39], visualized using Seaview v.4 [40], and multiple-sequence alignments were trimmed by applying Gblocks [41] with default options. One amino acid alignment for each ortholog was used to reconstruct phylogenetic trees using maximum likelihood implemented in RaxML v8.2.4 [42]. Models were automatic selected by AIC criteria using PROTGAMMAAUTO option. The robustness of the inferred trees was tested by bootstrap analyses (1000 replicates). Selective pressures acting on each ACDp at specific branches of phylogenetic trees (episodic diversification) were tested using the software HyPhy v.2.22 (https://veg.github.io/hyphy-site/), and the aBSREL (adaptive Branch-Site Random Effects Likelihood) method [43]. Positively selected branches were identified at a significance level of P > 0.05.

### Synteny analyses

Synteny was assessed by comparing the available genetic neighborhood from each ACDp from *L*. *major*, *T*. *brucei* and *T*. *cruzi (*TriTrypDB) using the Genome Browser tool present in TriTrypDB (S1 Fig) and expanded using BLAST to remaining draft genomes. Due to the ongoing nature of genome drafts, synteny could be only confirmed when large contigs were present in available genomes.

### ACD-like proteins expression profiles in transcriptomic and proteomic data

The analyses of ACDps in transcriptomic and proteomic databanks of *T*. *brucei*, *T*. *cruzi* and *Leishmania spp*. were conducted using the strategy search interface available in TriTrypDB (http://tritrypdb.org/tritrypdb/app/query-grid). ACDps expression profiles were investigated in each species and developmental stages. Details of datasets (including all links and references) examined are shown in S3 Table. The profiles herein determined depend largely on the quality of available transcriptomes, and proteomes, and negative searches of any protein may imply incomplete datasets.

## Results and discussion

### The kinetoplastid repertoire of ACD-like proteins comprises 9 genes classified in 8 families

**Fig 1:**
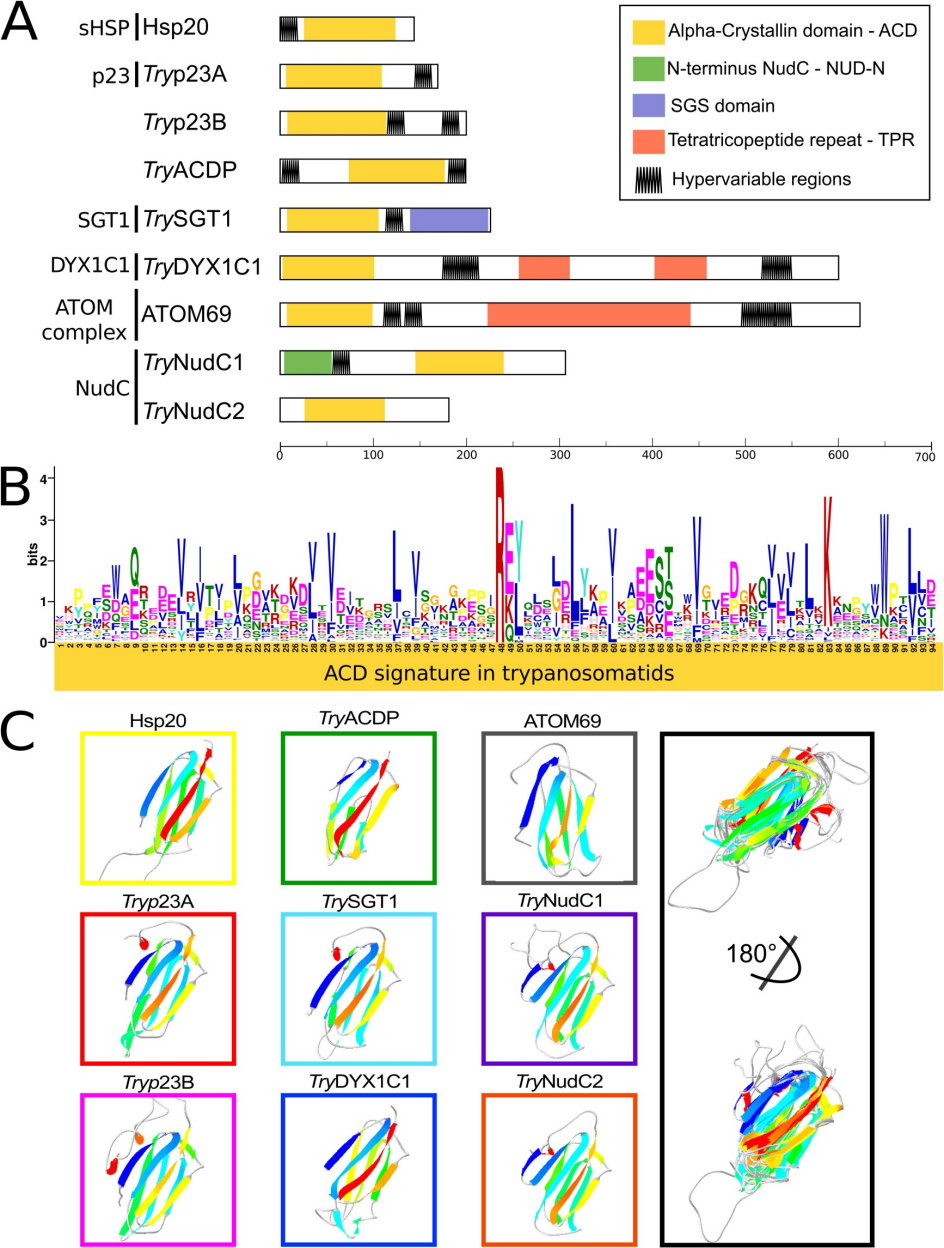
Domain architectures and signatures of kinetoplastid ACD-like proteins. (A) Schematic representation of protein length, conserved domains, and hypervariable regions of ACD-like proteins grouped by homology with archetypical families. (B) Amino acid logo representing p23/alpha-crystallin kye residues conserved in ACDps of trypanosomatids. Gapped motif search was conducted using GLAM2 software from MEME package. (C), Predicted tertiary structure of ACD present in ACDps of kinetoplastids, and ACD structural alignment of models inferred by Swiss Pdb-Viewer. Models were obtained from *T*. *brucei* TREU927 using SWISS MODEL, and visualized with Swiss Pdb-Viewer.

Our genome-wide surveys in genomes of 61 species of kinetoplastids (S1 Table) reveal 8 families (9 genes) of genes coding for ACDps in each genome. Our extensive surveys suggest that the repertoires disclosed herein are complete. As reported for other eukaryotes [16] predicted kinetoplastid ACDps largely diverged in N- and C-terminal sequences while sharing a conserved ACD. Classification of orthologs was based on the predicted protein structures comprising ACD, other domains, and hypervariable regions (Fig 1A). The previously recognized ACDp families Hsp20, p23 (p23A and p23B) and ATOM69 were identified in all genomes examined. Five additional proteins that share domains with those present in archetypical ACDps; i.e., *Try*ACDP, *Try*DYX1C1, *Try*SGT1 and *Try*NudC (*Try*NudC1 and *Try*NudC2 homologs), were characterized herein for the first time in kinetoplastids (Fig 1). All 8 ACD-like families are composed of orthologs conserved across the trypanosomatids and bodonids examined; most families have homologs in other eukaryotes, but Hsp20 is the only present in prokaryotes and eukaryotes [16]. The comparison of the ~100 amino acid ACD-like region of all nine proteins from each trypanosomatid genome revealed significant polymorphism, with the ACD signature composed of a few conserved hydrophobic residues (mainly V, I and L) and Arginine forming the motif R[E-K-Q][Y-L] (Fig 1B). However, the folding of all trypanosomatid ACDps (Fig 1C) predicted by the SWISS-MODEL homology-modeling server [37] confirmed that their tertiary structure is characteristic of proteins bearing ACD and ACD-like domains. The great divergence among trypanosomatid ACDps is compatible with divergence observed among ACD-protein families of other eukaryotes [16]. Sequence alignments and phylogenetic analyses demonstrated that *Try*SGT1, *Try*NudC1/*Try*NudC2, *Try*DYX1C1 share relevant amino acid identities and display a remarkable degree of structural conservation with counterparts from other eukaryotes, including invertebrates, vertebrates and some protists, providing strong support for our conclusion that these proteins are novel members of ACDp families.

**Fig 2:**
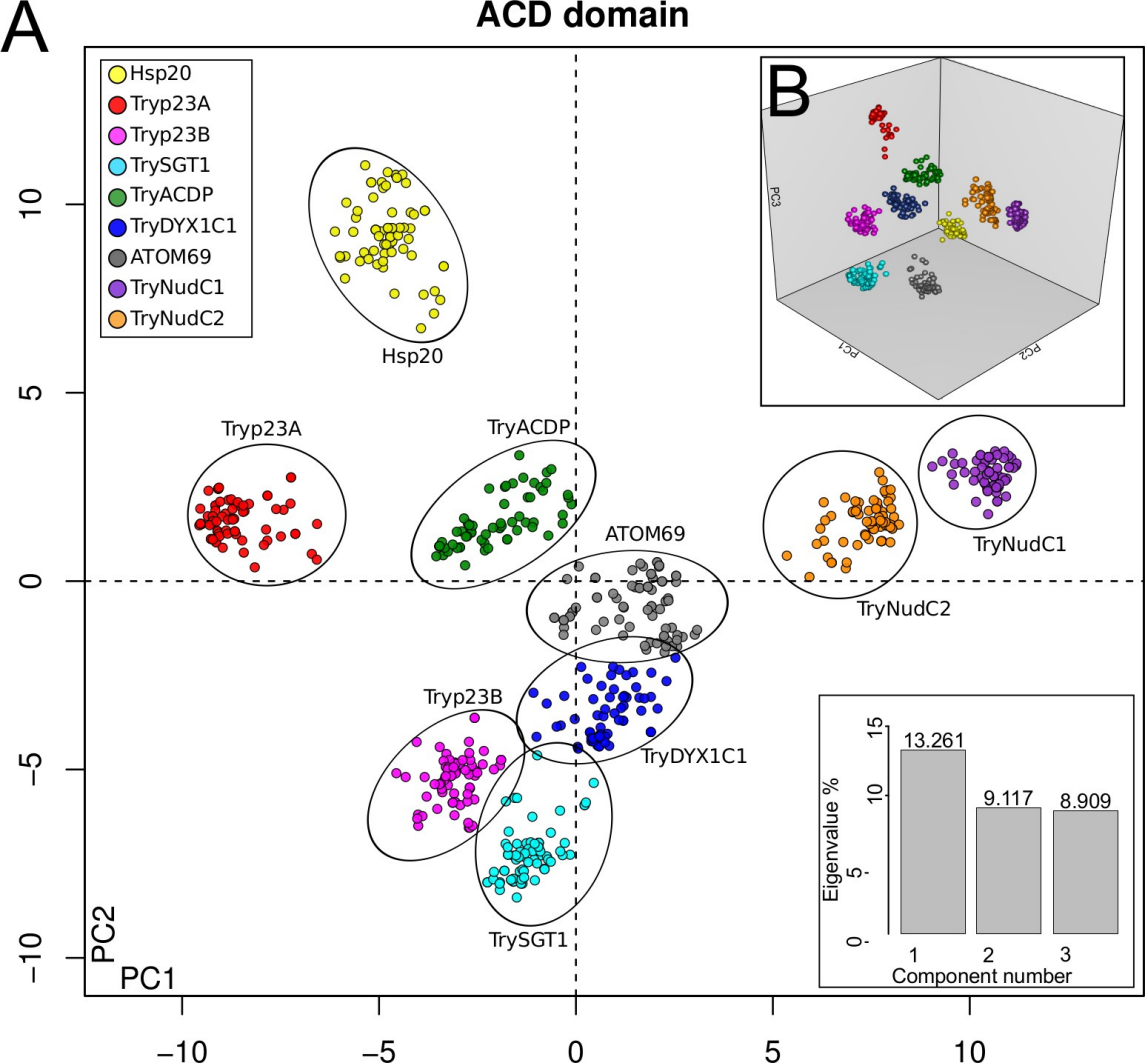
Multidimensional scaling (MDS) analysis of *Try*ACDpse amino acid sequences. MDS plots obtained using Bios2mds R-package showing the amino acid identity space in two (A) and three (B) dimensions.

The MDS analysis performed using all ACD-like sequences revealed 9 clusters (one formed by nearby but non-overlapping clusters of *Try*NudC1 and *Try*NudC2 homologs) representing 8 families (Fig 2). The HSP20 sequences formed a tight cluster distinct from those representing all other families, reflecting its apparent early divergence from a probable common ancestor of all ACD protein families (Fig 2). The position of the Hsp20 family as the most distant from all other ACDps, and its ubiquity in all kingdoms favors the hypothesis of one sHSP ancestor giving origin to all ACDps [16].

The nine genes identified in all surveyed genomes of trypanosomatids were also detected in the closest related *Bodo* sp. and *Bodo saltans*, and the more distant related *Parabodo caudatus*, suggesting that the ACDp repertoire herein identified is conserved across the kinetoplastids, including those of commensal and parasitic life. A blast search in very recently published genome of *Paratrypansoma confusion*, a unique insect parasite positioned between bodonids and other trypanosomatids [44], successfully recovered all ACDps shared by the other kinetoplastids. Therefore, diversification of all ACDps of kinetoplastid occurred prior to their adaptation to exclusive parasitic lifestyles. However, although most trypanosomatids exhibited a single copy of each ACDp, preliminary *Bodo* spp. and *P*. *caudatus* genome surveys revealed at least three extra p23 families (in addition to p23A and p23B), and additional nearly identical copies of p23A, p23B, TryACDp, and NudC2. A few identical copies of *Try*ACDP, DYX1C1 and NudC families were also recovered from a few trypanosomatids (S4 Table). The relative positions of the trypanosomatid species showing additional copies in kinetoplast phylogeny indicates that paralogy in trypanosomatids is a species-specific trait, probably generated by recent gene duplication (detailed below in each ACDp sections and in S4 Table).

Whether bodonids possess additional families or copy numbers of ACDps requires further wide-genome scans for more divergent ACD-like domains. Previous comparative genomics evidenced streamlining of gene families in trypanosomatids compared to *B*. *saltans* [45]. More compacted genomes of trypanosomatids may have been shaped by their adaptation to physiologically narrower and more constant host environments. On the other hand, expansion of species-specific multigene families playing fundamental roles in cellular invasion, and immune evasion is a common feature of pathogenic trypanosomes, and leishmanias [46–48].

ACD proteins of a broad diversity of eukaryotes includes different families such as B5+B5R (Favo-hemo cytochrome NAD(P)H oxidoreductase type B), Melusin (Integrin beta-1-binding protein 2), RAR1-like (cysteine and histidine-rich domain-containing protein 1-like) and CacyBP (Calcyclin-binding protein) [16], that were not detected in our initial genomic surveys of trypanosomatid genomes. Therefore, we attempted to discover additional ACD families not identified by RPS-BLAST using sequences of the archetype described for each of these families as queries. These searches identified putative orthologs for these families in kinetoplastids, but the ACD was not found in their domain architecture (S2 Table). The domain architecture of B5+B5R homologs is formed only by Cytochrome B5 reductase domain in kinetoplastids, whereas human archetype (Q7L1T6) possesses a Heme/Steroid binding domain followed by ACD and Cytochrome b5 reductase domains. In addition, the ACD is also related to the CHORD domain in Melusin (Q9UKP3), and RAR1-like protein families. However, our searches in kinetoplastids identified homologs to these proteins containing the CHORD domain but lacking the ACD. Finally, our search did not recover any homologous of CacyBP in kinetoplastids. Human CacyBP (Q9HB71) displays an architecture formed by a Siah interacting domain (N-terminal), ACD-like, and SGS-like domain in the C-terminus. Thus, it appears that the ACD domain is broadly dispersed among eukaryotic genes, and may be preferentially gained by some genes in more complex eukaryotes for functions yet to be determined. A summary of ACDp analyzed herein and present in other organisms are present in Table 1.

**Table 1:** ACD protein families and some activities in prokaryotes and eukaryotes.

ACD Family	Prokaryotes^P^	Trypanosomatids^T^		Yeasts^Y^	Plants^G^	Vertebrates^V^
**HSP20**	Chaperone virulence factor, Stress tolerance [7,8,26]	Chaperone, stress tolerance, adaptation to host microenvironment [7,8,10–13,16,18,19,26,29]	LmjF.29.2450 Tb927.3.3330 TcCLB.508153.270 TcCLB.510323.40	Chaperone stress tolerance [7,8,16,18,19,26]	Chaperone, stress tolerance, tissue development regulation[7,8,16,18,19,23,26]	Chaperone, crystallin transparency, stress tolerance, cell development [7,8,16,18,19,26]
**p23**	Absent	Protection against HSP90 inhibitors, cochaperone[24,30,31]	Lbp23A LmjF.35.4470 Tb927.9.10230 TcCLB.507007.70 TcCLB.509551.70 Lbp23B LmjF.34.0210 Tb927.10.2620 TcCLB.506195.170 TcCLB.506407.60	Chaperone steroid receptor chaperoning[16,24]	Regulation of root development [16,23,24]	Chaperone, prostaglandin synthase activity, steroid receptor chaperoning[16,26]
**SGT1**	Absent	Unknown	LmjF.20.1640 Tb927.1.3200 TcCLB.507837.60 TcCLB.508405.30	Chaperone activities and kinetochore assembly[49]	Defenses against pathogens, development regulation[20]	Inflammasome assembling and activity[21]
**DYX1C1**	Absent	Unknown	LmjF.32.2850 Tb927.11.16050 TcCLB.508707.120 TcCLB.509073.90	Unknown	Unknown	Chaperone and cochaperone, neuronal migration, cilia structure, human dyslexia, steroid receptor[25]
**NudC**	Absent	Unknown	*Try*NudC1 LmjF.14.0450 Tb927.7.4290 TcCLB.508089.30 TcCLB.508857.50 *Try*NudC2 LmjF.30.0700 Tb927.6.2130 TcCLB.507019.100	Dynein-dependent nuclear migration, chaperone and cochaperone[50]	Thermotolerance, development regulation, chaperone[50]	Chaperone and cochaperone, neuronal migration, thrombopoiesis, megakaryocytopoiesis, cilia structure, monocyte-macrophage differentiation, dynein-dependent nuclear migration[22,50,51,52]
**ATOM69**	Absent	Mitochondrial receptor protein import into kinetoplast [32,33]	LmjF.28.2170 Tb927.11.11460 TcCLB.511803.40	Absent	Absent	Absent
***Try*ACDp**	Absent	Unknown	LmjF.32.2260 Tb927.11.15480 TcCLB.509267.110 TcCLB.510031.70	Absent	Absent	Absent

### The conserved small heat shock protein (sHSP) of kinetoplastids

sHSPs, ranging from 12 to 42 kDa, are ubiquitous in archaea, bacteria and eukarya, and sequence homology supports the evolutionary conservation and constitutive function of this protein as a chaperone. In addition, sHSPs have been implicated in stress tolerance, apoptosis and immune response [53–54] (Table 1). The number of sHSPS ranges from one in yeast, four in *Drosophila*, 11 in humans (hspB1-B10), 14 in *C*. *elegans*, 16 in silkworm and *Xenopous laevis*, to more than 30 in plants [26,53,55]. Our wide genome survey of Hsp20 orthologs disclosed a single copy in each genome examined, corroborating previous findings in *T*. *cruzi* (sHSP16), *T*. *brucei* (HSP20) and *L*. *major* (HSP20/HSP23) [10,13,28]. The only exception was the presence of additional copies in the draft genome of *T*. *congolense*. HSP20 fragments were recovered in *T*. *theileri* (TCC165) genome draft, however a full-length copy of HSP20 (accession: TM35_000013200) is present in the public available *T*. *theileri* (Edinburgh) genome. The Hsp20 of the kinetoplastids exhibited a single hypervariable region at the beginning of N-terminus, and no domains other than the ACD (Fig 1). Analysis of aligned HSP20 full-length amino acid sequences of trypanosomatids revealed high conservation within *Leishmania* (~90% identity) and *Trypanosoma* (~63%), representing the most conserved ACDp found in kinetoplastids.

Despite increased transcription of HSP mRNA triggered by heat shock, due to polycistronic transcription and post-transcriptional control of gene expression in kinetoplastid, HSPs expression is regulated by mRNA degradation through specific RNA-protein interactions [14]. Transcriptomic profiles showed HSP20 expression in insect (epimastigotes and promastigotes) and vertebrate (trypomastigotes and amastigotes) stages of *T*. *cruzi*, *T*. *brucei* and *L*. *major*. Proteomic profiles revealed HSP20 in insect stages of all these species, and also in trypomastigotes of *T*. *brucei* and amastigotes of *Leishmania mexicana* (proteome of *L*. *major* amastigotes is not available), but not in amastigotes of *T*. *cruzi* (S2 Fig). sHSPs are over-expressed by trypanosomes and leishmanias submitted to heat stress, consistent with a role of these proteins in their adaptation to vertebrate hosts [10, 12, 13, 29]. Accession links and references from transcriptomic and proteomic data set analysed in this study are detailed in S3 Table.

### Independent evolution of p23A and p23B orthologs in kinetoplastids supports the division in *Try*p23A and *Try*p23B families

The p23 family is exclusive to eukaryotes and conserved from fungi to vertebrates. Members of the p23 family are the smallest partners of the HSP90 machinery with a simple structure formed by one ACD domain, and a flexible tail at the C-terminus. Proteins of this family act as chaperones or co-chaperones and participate in a complex spectrum of cellular regulatory pathways including steroid receptors, amino acid biosynthesis, cell-signaling kinase pathways, and apoptosis (Table 1). The multifunctional role is due to the interaction of p23 with HSP90 and diverse client proteins such as polymerases, transcription factors, nitric oxide synthase, and protein kinases (Table 1) [16, 24].

In the kinetoplastids, p23 genes were previously described in the genomes of *L*. *major*, *T*. *cruzi* and *T*. *brucei* [10]. Two p23 proteins, Lbp23A and Lbp23B, were identified in *L*. *braziliensis*. Putative orthologs were found in *T*. *cruzi* (Tcp23A and Tcp23B), and *T*. *brucei* (Tbp23A and Tbp23B) [30, 31]. Orthologs of p23A and p23B retrieved from trypanosomatid genomes encode ~180 and 200 amino acid proteins, respectively, lacking any conserved domain other than the ACD. While p23A displays a hypervariable region rich in GG[VLM] motifs followed by DD and EE repetitions, p23B shows a hypervariable region with fewer repetitions of GGL, and DD flanking a region rich in SN, SS and NN motifs (Fig 1, S3 Fig). Amino acid sequences of the ACD-like domains of p23A and p23B orthologs show ~24% of identity among trypanosomatids. ACDp clustering inferred by MDS argues that *Try*p23B is more closely related to *Try*pDYX1C1 and *Try*pSGT1 than to p23A (Fig 2).

**Fig 3:**
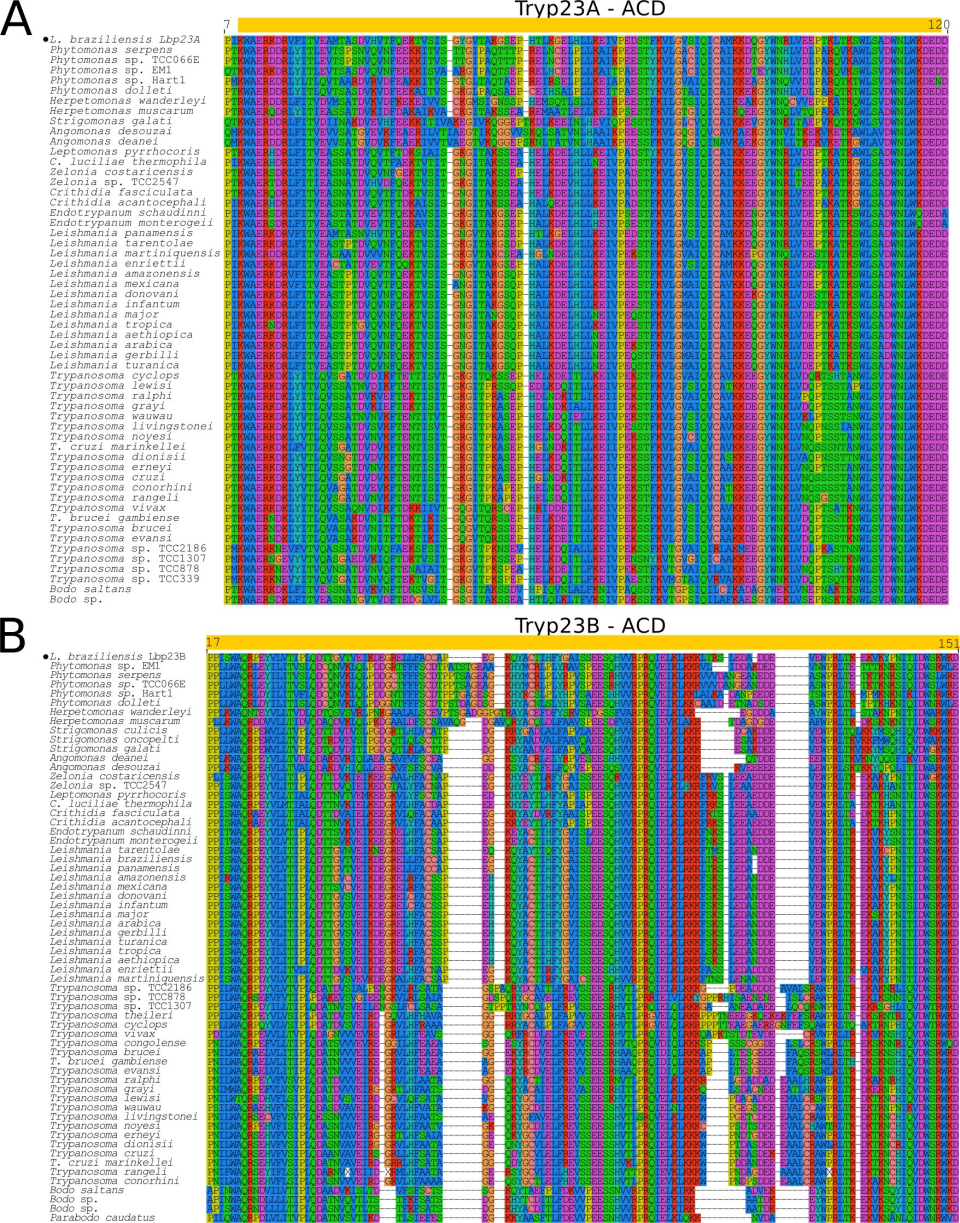
Multiple sequence alignment of ACD from Tryp23A and Tryp23B. (A) *Try*p23A and (B) *Try*p23B alignments carried out using MUSCLE v3.8.31 showing conserved and variable sites. Yellow box indicates the position of ACD, and black filled dots the archetypes for Tryp23A and Tryp23B.

Taking into account notable differences between p23A and p23B regarding sequence identity and divergence, domain architecture, and predicted tertiary structure Figs 1–3, we proposed their separation into two families and two evolutionary lineages: *Try*p23A and *Try*p23B. This proposal is consistent with previous reports of low identity (30%) between Lbp23A and Lbp23B of *L*. *braziliensis*, and differences regarding chaperone activities (although both proteins interact with HSP90), thermal and chemical stabilities, all suggesting functional differences [30] despite sharing p23 domains. This previous study identified both proteins in *L*. *infantum*, *L*. *major*, *L*. *donovani*, *T*. *cruzi*, *T*. *brucei*, and *T*. *vivax*. In general, a single copy of each *Try*p23A and *Try*p23B ortholog was recovered from each trypanosomatid genome. However, multiple copies were identified in the genomes of *T*. *b*. *gambiense* (*Try*p23A and *Try*p23B), *T*. *congolense* (*Tr*yp23A), *T*. *theileri* and *T*. *lewisi* (*Try*p23B).

Transcriptome profiles of *T*. *brucei*, *T*. *cruzi* and *Leishmania* spp reveal that *Try*p23A and Tryp23B are both constitutively expressed in all life cycle stages (S3 Table and S2 Fig). *Try*p23A proteins were detected in insect and vertebrate stages of *T*. *brucei* and *T*. *cruzi* as well as in insect stages of *L*. *major* and *L*. *braziliensis*. Although *Try*p23A protein was not detected in amastigotes of *L*. *mexicana* could, its ortholog was identified in amastigotes of *L*. *donovani* (accession: LdBPK_354540.1.1). Furthermore, *Try*p23B was not detected in the proteomic profiles of amastigotes of *L*. *mexicana*, and in amastigotes and epimastigotes of *T*. *cruzi*, whereas all stages of *T*. *brucei* express this protein (S2 Fig) (Accession links and references of transcriptomic and proteomic databases analyzed in this study are detailed in S3 Table). Previous functional studies demonstrated that although p23 mRNA levels rise in intracellular amastigotes of *L*. *donovani*, protein abundance remains unaffected, and p23 null mutants displayed no difference in infectivity but exhibited a marked hypersensitivity against Hsp90 inhibitors [30,31]. The fact that *L*. *braziliensis* p23A is more stable than p23B together with species-specific and stage-regulated expression patterns suggests distinct roles for *Try*p23A and *Try*p23B, depending on species and stages of parasites. Interestingly, a recent search for proteins harboring p23 domains in the *Plasmodium falciparum* genome also reveal two p23 proteins showing small (13%) amino acid identity to each other and playing different functions [56].

### Mitochondrial ATOM69 (translocase of the outer membrane) is ubiquitous across kinetoplastids

Mitochondrial proteins are translocated across the mitochondrial double membrane by translocases of the inner membrane (TIM) and the outer membrane (TOM) complexes, the machinery responsible for transport of nuclear encoded proteins essential to maintain mitochondrial function, whose origin likely coincided with the origin of eukaryotes (Table 1). The TOM complex of eukaryotes comprises one channel-forming b-barrel protein and six additional subunits, each exhibiting a helical trans-membrane segment [57]. The recently discovered kinetoplastid-specific ATOM complex comprises six subunits, ATOM40, ATOM14, ATOM46, ATOM69, ATOM11 and ATOM12. Only ATOM69 is an ACD-like protein [32,33].

ATOM69 proteins are integral (mitochondrial) membrane receptors exposed to cytosol that display a typical domain architecture formed by an N-terminal ACD-like domain, and a central tetratricopeptide repeat (TPR) domain followed by a C-terminal hypervariable region (Fig 1) with no known homology to translocases of any other organisms. Knockdown of ATOM69 causes growth arrest in *T*. *brucei* in both mammalian and insect stages (Table 1). Prior to the present study, ATOM69 orthologs were reported in *T*. *cruzi*, *T*. *brucei*, *T*. *vivax*, *T*. *congolense*, *L*. *major*, *L*. *mexicana*, *L*. *braziliensis*, *Strigomonas culicis*, *Angomonas deanei*, *Crithidia fasciculata*, *Phytomonas*, and *Bodo saltans* [32]. In general, a single copy of ATOM69 encoding a ~600 amino acid protein with ~66% identity across taxa, was identified in each genome. Of the 58 trypanosomatid species investigated herein, only species of *Phytomonas*, *Strigomonas*, and *Angomonas*, and *T*. *congolense* have additional copies of ATOM69 genes (S4 Table). Expression patterns confirmed ATOM69 transcripts and proteins in insect and vertebrate stages of *Leishmania* (including amastigotes) and *T*. *b*. *brucei*, but this protein was not detected in proteomes of intracellular amastigotes of *T*. *cruzi* (S2 Fig) (Accession links and references of transcriptomic and proteomic databases analyzed in this study are detailed in S3 Table).

### Kinetoplastids have unique SGT1-like proteins lacking the TPR domain

SGT1 (Suppressor of the G2 allele of SKP1) is a subunit of the SCF (SKP1/Cullin-1/F-box protein) class of E3 ubiquitin ligases functioning as a cochaperone of Hsp90 in folding and degradation pathways. SGT1 proteins are conserved in plants, animals and yeast, and likely evolved to cover diverse functions in physiological and pathological processes (Table 1) [20]. This protein is up-regulated by heat shock and other stress conditions, has been implicated in the innate immune response in mammals, pathogen defense in plants, and kinetochore assembly in yeast. The overexpression of SGT1 in tumor tissues suggest that this protein contributes to stabilizing oncoproteins involved in cell proliferation (Table 1) [20,58].

The SGT1 architecture includes three conserved domains: a TPR in the N-terminus, the central ACD-like, and a highly conserved SGT1-specific domain in the C-terminus. These domains are separated by V1 and V2 hypervariable regions. The TPR domain, present in many eukaryotic SGT1 proteins, is involved in chaperone machinery, cell-cycle and protein transport (Table 1). This domain comprises tandem repeats of 34 amino acids that mediate protein-protein interactions and multi-protein complex assembly [59]. Almost nothing is known about SGT1 in kinetoplastids although in *T*. *brucei*, SGT1 mRNA increases after heat shock in the presence of ZC3H11, a zinc finger protein that acts as a post-transcriptional regulator of chaperone [14].

**Fig 4:**
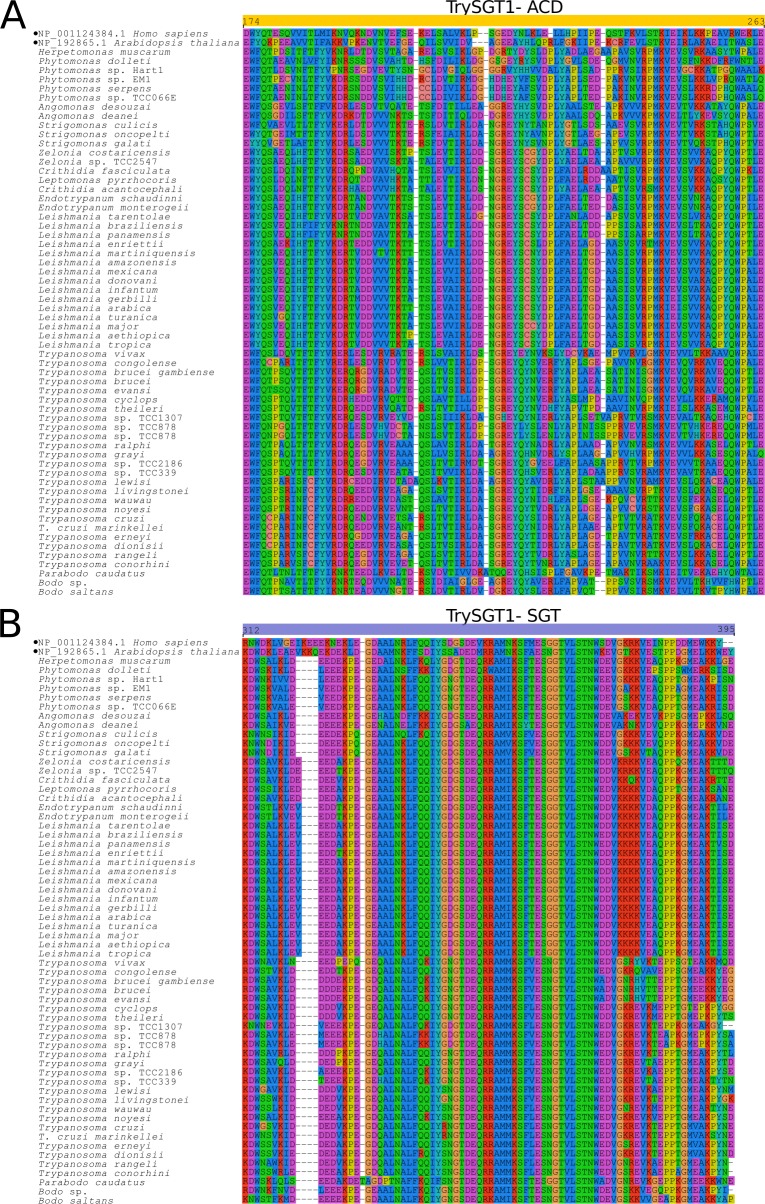
Multiple sequence alignments of ACD and SGT domains of TrySGT1. Multiple sequence alignment of conserved domains in TrySGT1 orthologs: (A) ACD; (B) SGT. Conserved and variable sites are indicated in the alignment (MUSCLE v3.8.31). Yellow and blue boxes mark the position of ACD and SGT, respectively, and black filled dots marks the archetype for STG1 in other eukaryotes.

*Try*SGT1 genes encode a ~ 215 amino acid protein, lacking a signal peptide and showing ~ 65% amino acid identity among the orthologs of trypanosomatids. Notably, *Try*SGT1 exhibits a unique domain architecture comprising just two (SGT1-like and ACD-like) of the three domains present in human SGT1 archetype; i.e., *Try*SGT1 lacks the TPR domain plus the first hypervariable region in the N-terminus (Fig 1). Although encoding a larger protein of 292 amino acids, the SGT1 genes of *Bodo saltans*, *Bodo* sp. and *Parabodo caudatus* also lack a TPR domain (Fig 4) suggesting that loss of TPR domain was an ancient event in the evolution of the kinetoplastids. Functions of the SGT1 protein and the TPR domain in protists are virtually unknown. Over-expression of SGT1 in *Glaciozyma antarctica* protected this yeast from high and low temperature stresses [49]. Prior to the current study, only one protein of the SGT1 network was annotated from a kinetoplastid genome: *T*. *brucei* (Tb11.02.3990) SKP1-like protein. In a study with *Toxoplasma gondii*, SKP1 was involved in parasite detection of oxygen levels and genomic surveys suggested the existence of an ancestral SKP1 glycosylation pathway in other Apicomplexa species, and in protists in general [60]. We searched for SKP1-like genes using *T*. *brucei* SKP1 (Tb927.10.11610/Tb927.11.6130) and recovered a single SKP1-like gene from each genome examined, suggesting that a non-predicted interaction between SKP1/SGST1 may occur in the trypanosomatids. The genomic search revealed, in general, a single copy of *Try*SGT1. The exception was a frog trypanosome (*Trypanosoma* sp TCC878) with multiple nearly identical copies. MDS analyses clustered all *Try*SGT1 orthologs near to *Try*p23B, *Try*DYX1C1 and *Try*ACD-TPR clusters (Fig 2). Transcripts of *Try*SGT1 are constitutively expressed in all life stages of *T*. *brucei*, *T*. *cruzi*, and *Leishmania* spp. We detected *Try*SGT1 in proteomic profiles of different stages of *T*. *brucei* and *Leishmania*, but not detected in any *T*. *cruzi* developmental stages (S2 Fig) (Accession links and references of transcriptomic and proteomic databases analyzed in this study are detailed in S3 Table).

### NudC-like *Try*NudC1 and *Try*NudC2 homologs are ubiquitous in kinetoplastids

Nuclear distribution C (NudC) proteins are conserved from fungi to mammals acting as chaperones and holdases that stabilize or enhance the folding of client proteins. Besides their multiple roles in the cell cycle, evidence has accumulated that NudC proteins have acquired new functions in metazoans, being implicated in many processes such as mitosis, neuronal differentiation and migration, regulation of inflammatory response, platelet production, cytoskeleton dynamics, and proliferation of tumor cells (Table 1) [22,50,51,52].

**Fig 5:**
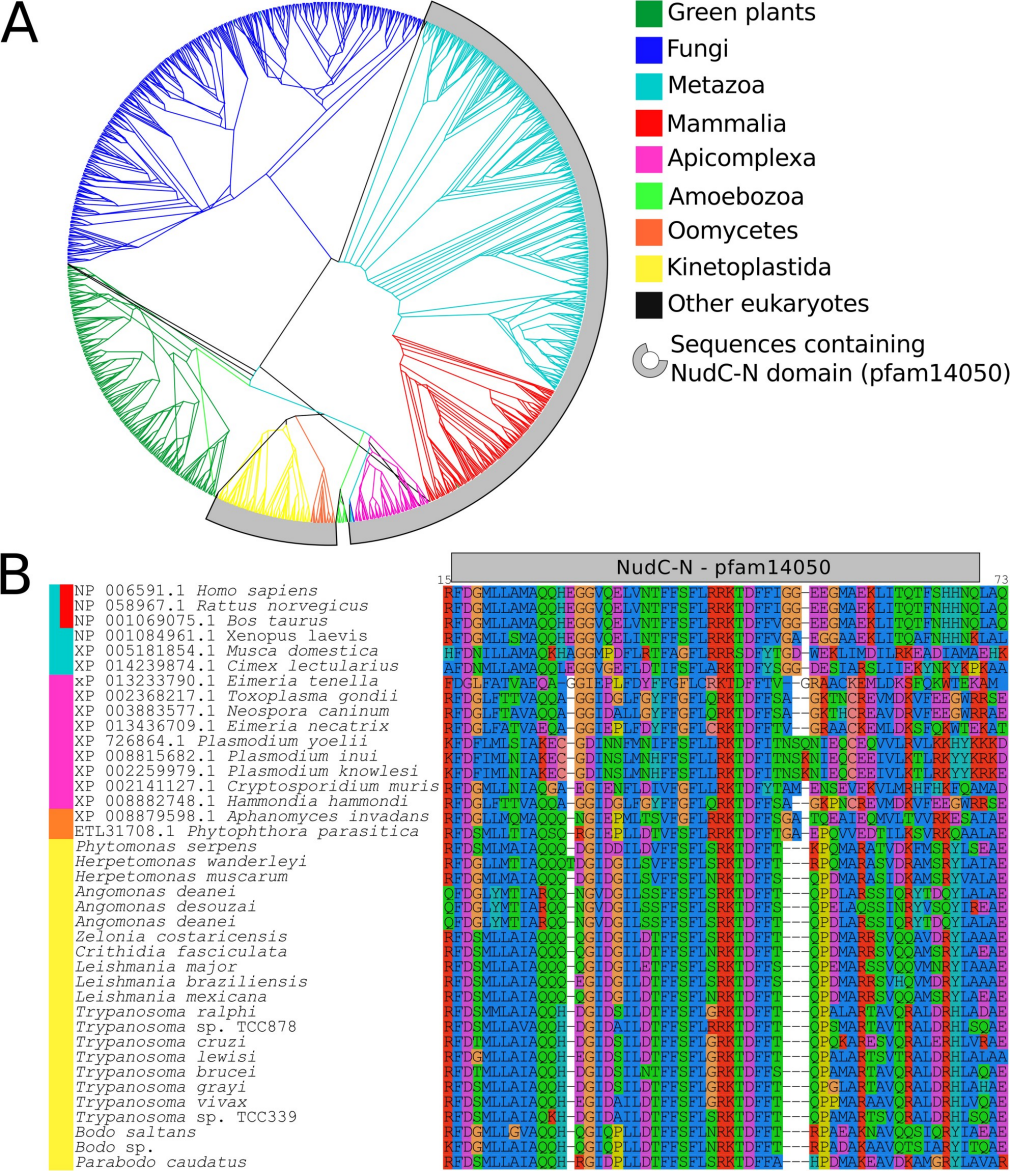
Phylogenetic conservation of NudC-N domain in NudC homologs. (A) Comprehensive genealogy of NudC1 homologs available on NR NCBI database. Eukaryotic sequences with NudC-N domain (pfam14050) are marked in gray, and branches are colored according to taxonomic groups. The access numbers of sequences employed in the analysis are in S1 Table. (B) Multiple sequence alignment of N-NudC domain (pfam14050) representatives of each taxonomic group present in (A).

All members of the NudC family comprise one ACD domain. Conserved NudC homologs have been identified in a range of eukaryotes and three NudC genes, NudC, NudC-like (NudCL) and NudC-like 2 (NudCL2), have been identified exclusively in vertebrates [22]. Our searches of ACD-like domains in the kinetoplastid genomes revealed two NudC homologs, here termed *Try*NudC1 and *Try*NudC2 that clustered closest to each other but did not overlap in MDS analysis (Fig 2). *Try*NudC1 proteins (~315 amino acids) display domain architecture like the human homolog hNudC (NP_006591.1) with the NudC-N domain in the N-terminal extension, and a hypervariable region between residues 66–88 (Fig 1). NudC-N domains of all *Try*NudC1 share ~70% amino acid identity and are quite conserved (~60% amino acid identity) to human NudC-N domain (NP_006591.1). Part of the amino acid alignment of *Try*NudC1 sequences is shown in the Fig 5B. The full-length alignment of *Try*NudC1 orthologs from kinetoplastids and other eukaryotic lineages is shown in S3 Fig.

**Fig 6:**
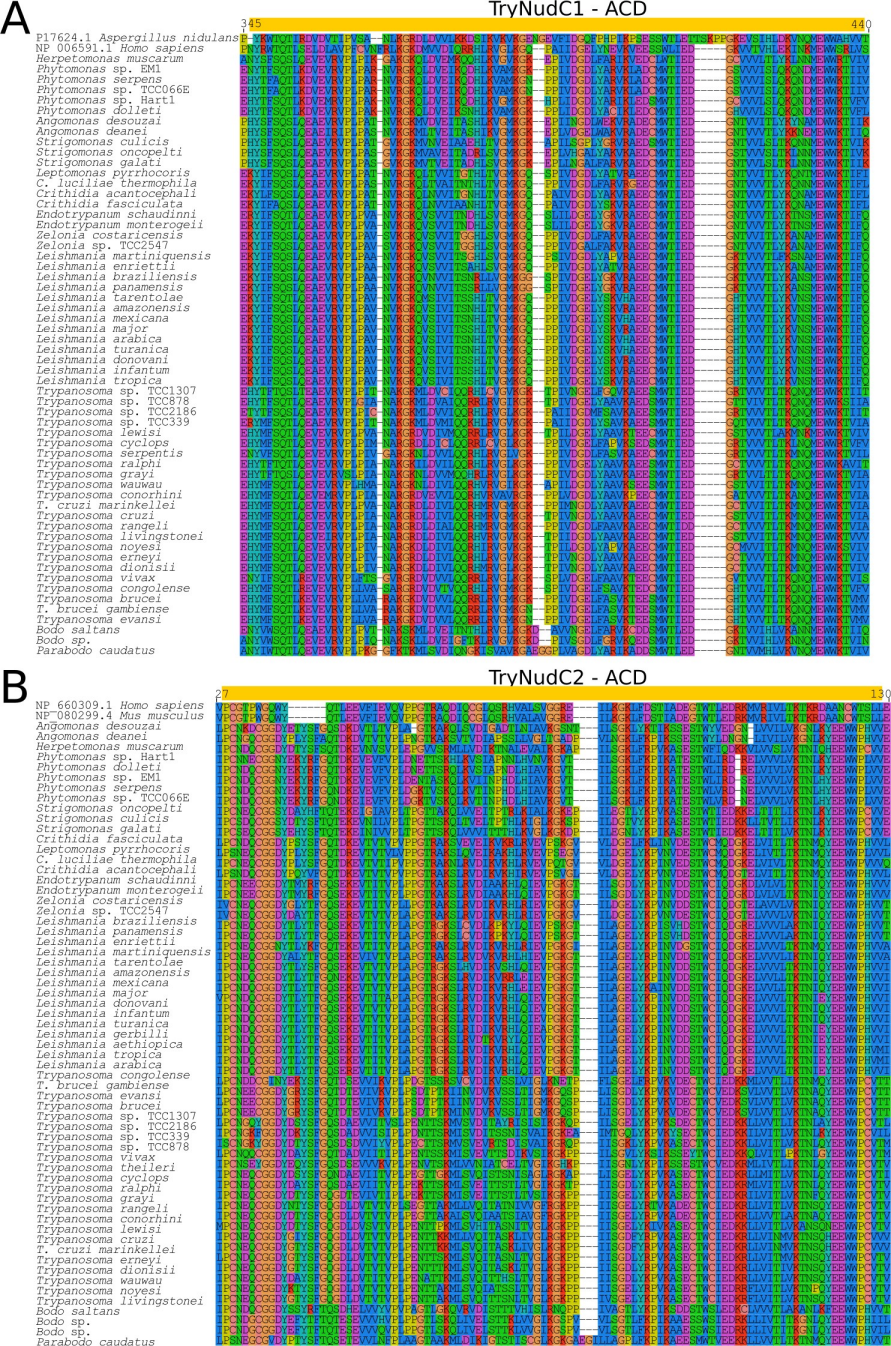
**Multiple sequence alignment of ACD sequences from *Try*NudC1 and *Try*NudC2.** Multiple sequence alignment (MUSCLE v3.8.31), showing conserved and variable sites, of kinetoplastid orthologs and archetypes of *Try*NudC1 (A) and TryNudC2 (B). Yellow boxes indicate the ACD and black filled dots the archetype described in other eukaryotes.

In addition to *Try*NudC1 homologs of human NudC, the kinetoplastids exhibited a NudC homolog hereafter referred to as *Try*NudC2 due to its similarity with NudCL2. Consistent with *Try*NudC2 being more related to NudCL2, both are small proteins lacking the NudC-N N-terminal domain (Fig 1). *Try*NudC2 (~170 amino acids) shared ~62% identity among trypanosomatid orthologs. In contrast to *Try*NudC1, *Try*NudC2 proteins are highly divergent from the NudCL2 of both human (~20% identity) and fungi (~30%). The alignment of *Try*NudC1 and *Try*NucC2 sequences is illustrated in the Fig 6 and S3 Fig. The high degree of conservation of the N-terminal between *Try*NudC1 and mammalian orthologs led us to investigate NudC-N domains (pfam14050) in other organisms. This analysis revealed NudC-N domains, similar to the *Try*NudC1 orthologs in trypanosomatids, protists of Alveolata, *Plasmodium*, *Eimeria*, *Neospora* and *Toxoplasma*, but not in those of Amebozoa, Fungi and green plants (Fig 5A and 5B and S3 Fig). The NudC-N domain was implicated in the regulation of inflammatory pathways in mice by reducing the activity of PAF, a pro-inflammatory messenger that regulates monocyte-macrophage differentiation (Table 1) [52]. NudC proteins were rarely studied in protists. A recent study demonstrated that the expression of a NudC protein by *Entamoeba histolytica* is up-regulated in multinucleated cells, and its overexpression induces larger and multinucleated trophozoites originated from cytokinesis failure [61]. This finding is consistent with the role of NudC proteins in human mitosis, with both depletion and overexpression inducing imperfect cytokinesis [62]. One study done with *Leishmania infantum* revealed an up-regulated NudC-like protein in human-infective metacyclic promastigotes [63].

The identification of putative *Try*NudC2 proteins in kinetoplastids is the first description of a member of NudCL2-like family in a protist, whose functions, similarly to *Try*NudC1, need to be investigated. *Try*NucC2 transcripts were identified in all organisms examined, but *Try*NucC2 protein was only detected in insect forms of *T*. *brucei* (S3 Table and S2 Fig). Overall, NudC proteins play essential roles in regulating cytoskeleton dynamics regulating the dynein pathway responsible for the trafficking of various cargo towards microtubules including endosomes, lysosomes and mRNAs, and depletion of NudC expression leads to the dispersion of the Golgi apparatus and perinuclear microtubules [22,59].

The analyses of *Try*NudC proteomic and transcriptomic profiles (S2 Fig) showed *Try*NudC1 transcripts and proteins in both vertebrate and insect stages of *T*. *brucei* and *T*. *cruzi*, whereas transcripts but not proteins were detected in amastigotes (resident exclusively of macrophages) of *L*. *mexicana* (S2 Fig) (Accession links and references of transcriptomic and proteomic databases analyzed in this study are detailed in S3 Table).

### Unveiling DYXC1-like proteins in kinetoplastids, a protein involved in cytoskeleton and cilia assembling in other organisms

Homologs of DYX1C1, the Dyslexia susceptibility 1 candidate gene 1 protein, have been found in several eukaryotes, ranging from protists to vertebrates. DYX1C1 displays an ACD in the N-terminus and a variable number of TPRs in the C-terminus (Fig 1). DYX1C1 proteins have many partners in multi-protein complexes related to varied functions such as cell migration, nervous system development, autophagy, and cytoskeleton (Table 1) [64,65].

**Fig 7:**
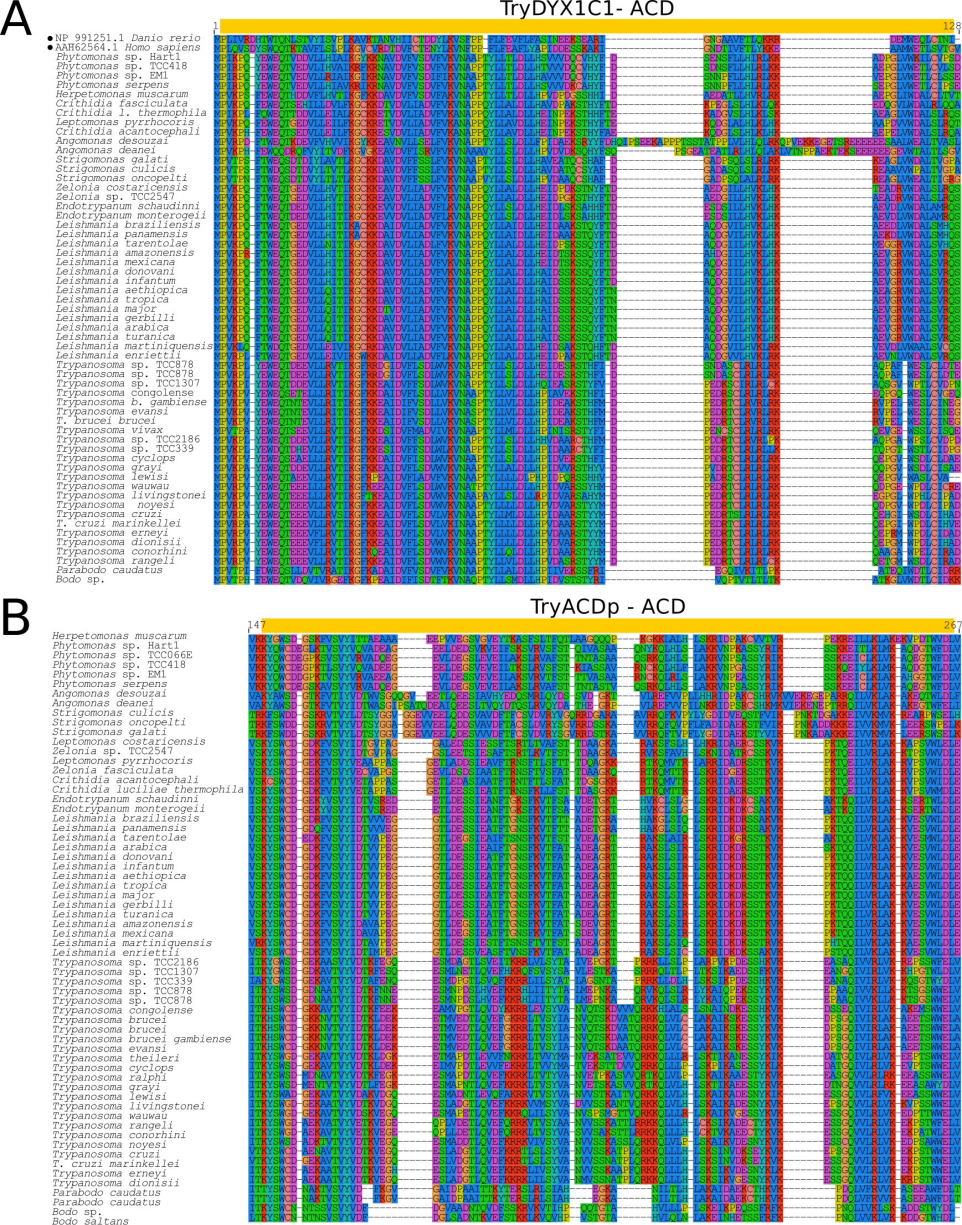
Multiple sequence alignment of ACD from *Try*DYX1C1 and *Try*ACDp sequences. Multiple sequence alignment (MUSCLE v3.8.31) showing conserved and variable sites by comparing kinetoplastid and archetypes sequences of *Try*DYX1C1 (A) and *Try*ACDp (B). ACDs and the archetypes described in other eukaryotes are marked with yellow boxes and filled dots, respectively.

Our genomic surveys recovered a single DYX1C1 gene copy in each trypanosomatid genome, with domain architecture (Fig 1A) and sequence similarity with the archetypical human DYX1C1 (Fig 7A) supporting the designation of these sequences as *Try*DYX1C1. Only *T*. *lewisi*, *T*. *ralphi*, and the frog *Trypanosoma* sp TCC878 showed two or more identical or nearly identical copies of *Try*DYX1C1 genes. We also recovered DYX1C1-like genes from the genomes of *Bodo* sp., *B*. *saltans* (partial sequence), and *P*. *caudatus* (S4 Table). As for other ACDps, sequences of *Try*DYX1C1 diverge to become species-specific, and comparison of full-length amino acid sequences of these trypanosomatid genes revealed only ~60% identity. *Try*DYX1C1 genes encode proteins ranging from 579 to 630 amino acids and share only (~25%) amino acid identity with the smaller 420 amino acid human archetype. Size and amino acid polymorphisms among *Try*DYX1C1 orthologs are mainly due to differences in the two hypervariable regions between residues 170–218 and 533–550 (Fig 1A and S3 Fig). Nevertheless, despite the large divergences from archetypical gene, the tertiary structure predicted for *Try*DYX1C1 confirmed their classification as ACD-like proteins (Fig 1C). Although the domain architecture of *Try*DYX1C1 is more akin to that of ATOM69 protein, MDS analysis places *Try*DYX1C1 near to *Try*p23B and *Try*ACDP (Fig 2), supporting its separation from other ACDps identified in kinetoplastids.

DYX1C1 homologs were identified in many flagellate protists such as *Neigleria*, *Trichomonas*, *Leishmania* spp., *T*. *cruzi*, *T*. *brucei*, and *Toxoplasma gondii* [64]. The function of DYX1C1 in promoting the recruitment, stabilization and organization of microtubules demonstrated in human cells [64] may also be important to the cell architecture of the kinetoplastids. Invasion of mammalian cells by *T*. *cruzi* involves host cell microtubule dynamics (Table 1). Cytoskeletal structures have vital roles in many processes essential to kinetoplastids such as flagella assembling and function, secretory/endocytic system, and movement of organelles [66]. To our knowledge, TryDYX1C1-like proteins of kinetoplastids were not previously characterized by structural and phylogenetic inferences, and their functions remain to be investigated.

Our searches of *Try*DYX1C1 in both transcriptomes and proteomes (S2 Fig) show that although the mRNA is transcribed in all stages of *T*. *brucei*, *T*. *cruzi* and *Leishmania*, protein expression may be stage-regulated and so far only detected in epimastigotes of *T*. *cruzi* and trypomastigotes of *T*. *brucei*. *Try*DYX1C1 protein was not detected in any stage of *Leishmania* spp. (S2 Fig) (Accession links and references of transcriptomic and proteomic databases analyzed in this study are detailed in S3 Table).

### The polymorphic *Try*ACDP is specific of kinetoplastids

Our extensive searching for proteins harboring ACD-like domains uncovered a novel gene family in all kinetoplastid organisms investigated. This gene, designed as *Try*ACDP, encodes proteins of ~ 200 amino acids with domain architecture more similar to those of p23 and HSP20, and therefore lack any other conserved domain (Fig 1A). In MDS analysis, *Try*ACDP sequences clustered between *Try*p23A and *Try*DYX1C1 (Fig 2).

Although orthologs of *Try*ACDP were recovered from the genomes of all kinetoplastids examined, these genes were the most polymorphic of all members of the ACD-like protein family. Comparison of full-length trypanosomatid *Try*ACDPs revealed only ~43% amino acid sequence identity. Nevertheless, the aligned ACD domain (Fig 7B) and the predicted tertiary structure of *TryAC*DP were compatible with ACDps (Fig 1C). *Try*ACDP appeared fragmented in the genome of *T*. *vivax* Y486 (~100 amino acids), although the gene fragment and flanking genes were preserved in a locus syntenic with *T*. *brucei* (S1 Fig), and no full *Try*ACDP was recovered from transcriptomes (*T*. *vivax* Y486 and TvLins). Present as single copy genes in almost all genomes, additional copies were so far identified in *T*. *brucei*, the frog trypanosome (TCC878) and *P*. *caudatus* (S4 Table).

To investigate the existence of proteins related to *Try*ACDP in other protists, we conducted a PSI-BLAST search against NR. Only after exhaustive searching (four rounds of PSI-BLAST), we recovered highly divergent sequences of proteins bearing ACD-like domain from pathogenic oomycetes *Saprolegnia*, *Phytophthora*, *Aphanomyces*, and *Plasmopara* (XP_009521390.1, XP_002905176.1, XP_008896317.1, CEG43972.1, XP_008605824.1, XP_009829214.1). However, no clear inference about orthology of kinetoplastid and oomycetes genes was possible because of a very low sequence similarity through entire sequences including ACD-like domains. Therefore, *Try*ACDP identified in the present study appears to form a kinetoplastid-specific family with an as yet unknown function. The analyses of transcriptomic and proteomic profiles (S2 Fig) demonstrated that *Try*ACDP genes are constitutively expressed through trypanosomatid life stages, but protein was not detected in epimastigotes of *T*. *cruzi* (S2 Fig) (Accession links and references of transcriptomic and proteomic databases analyzed in this study are detailed in S3 Table).

### Genomic organization of *Try*ACD proteins is highly syntenic in trypanosomatids

**Fig 8:**
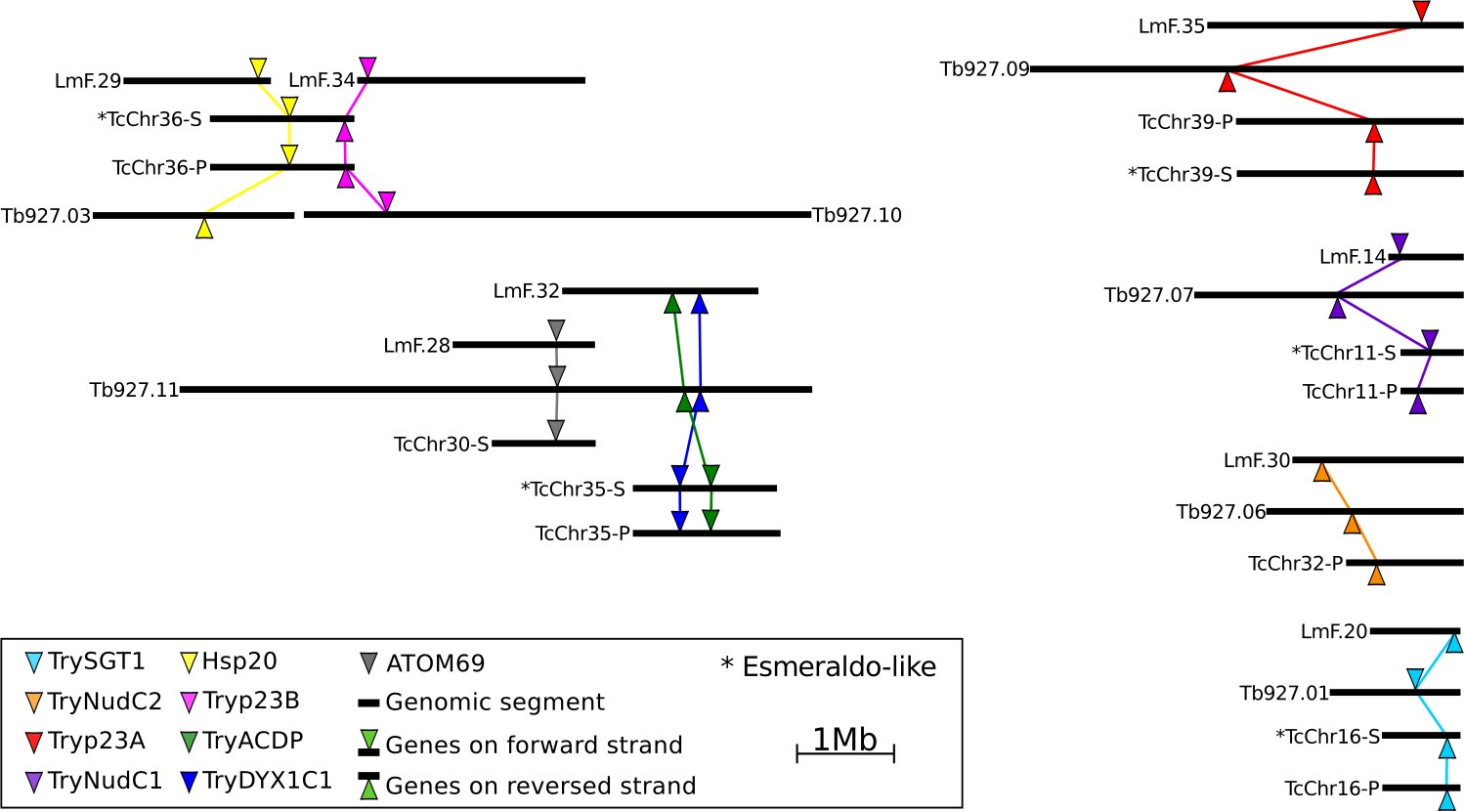
Genome organization of ACD protein locus in trypanosomatids. Chromosomal distribution of ACD proteins in the genomes of *T*. *brucei* TREU927, *L*. *major* Friedlin and *T*. *cruzi* CL Brener available in TriTryDB. Colored marks represent the respective position of each ACDp coding gene in assembled chromosomal segments. Syntenic loci from each gene are detailed in supplementary File 3.

Despite the ongoing nature of draft genomes hampering long-segment analyses, regions containing ACD genes and their neighborhood were shown to be predominantly syntenic, as herein illustrated with well-assembled genomes of *T*. *brucei* (TREU927), *T*. *cruzi* (CL Brener), and *L*. *major* (Friedlin). Synteny and gene neighborhood orientation indicate that ACDps belong to homologous genomic segments. The HSP20 and Tryp23B genes are in the same chromosomal segment in the alleles of both parentals (TcCh36-s and TcCh36-P) of the hybrid *T*. *cruzi* CL Brener. In *T*. *brucei* and *L*. *major*, HSP20 and *Try*p23B orthologs are in different chromosomes. *Try*p23A, *Try*SGT1, *Try*NudC1 and TryNudC2 are all present in syntenic genome segments, each in an individual chromosome. ATOM69, *Try*ACDP and *Try*DYX1C1 are co-located in a chromosomal segment in *T*. *brucei*, but not in *L*. *major* and *T*. *cruzi* genomes (Fig 8 and S1 Fig).

Overall existing as single copy genes (collapsed tandem repeats are undetectable in genome drafts), divergences in numbers of ACD genes in a few species is likely the result of gene loss or duplication. Preliminary analyses suggested that the more basal bodonids tend to present additional families and duplicated copies of ACD-like orthologs, suggesting some reduction of copy number during the transition from free-living to parasitic lifestyles. Interestingly, a lizard trypanosome (TCC 878) that belongs to the most basal clade of *Trypanosoma* was the trypanosomatid with the largest number of duplicated genes (SGT1, DYX1C1, NudC1 and *Try*ACDp), thus also suggesting the loss of duplicated ACD-like genes by most trypanosomatids. This feature is not conserved in trypanosomes from toads (TCC 339) or frogs (TCC2186 and TCC1307), which are very closely related to that from lizard, all belonging to the basal aquatic clade. More than one copy of ATOM69, *Try*p23A and *Try*p23B genes were detected in other trypanosomatids, whereas other genes such as HSP20, DYX1C1 and SGT1 rarely present more than one copy (S4 Table).

### Phylogeny and episodic diversifying selection in ACD-like proteins of kinetoplastids

**Fig 9:**
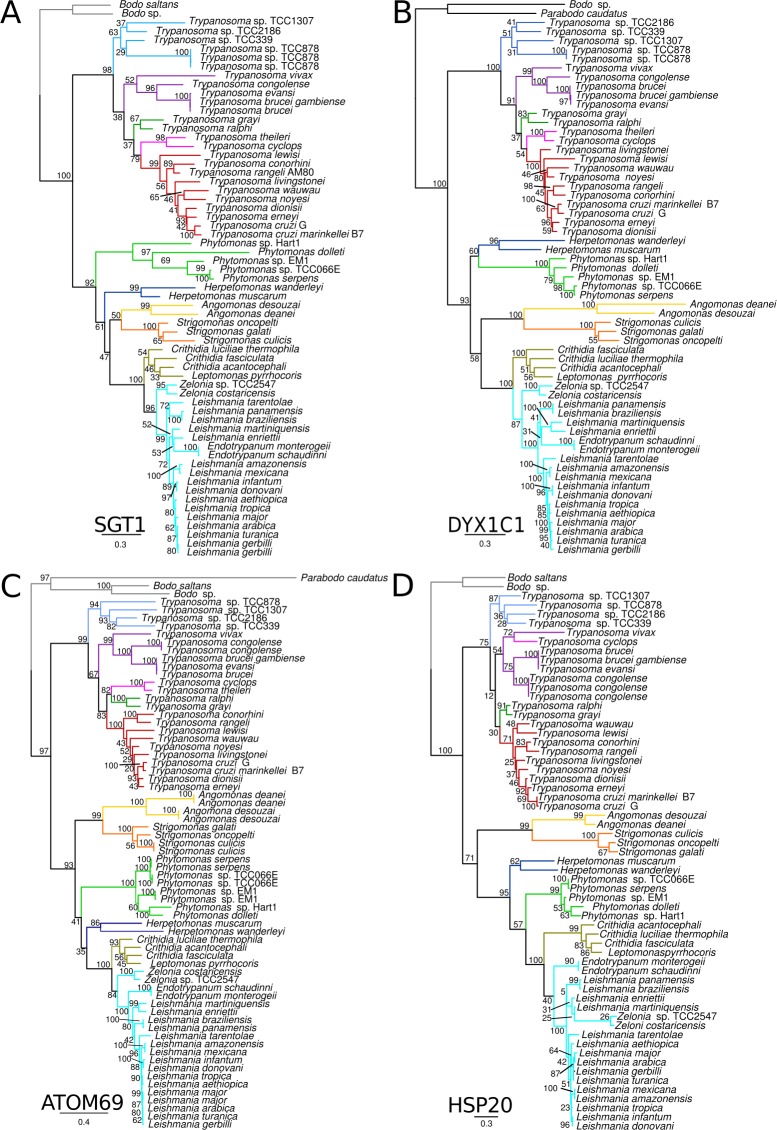
Kinetoplastid genealogies of ACD protein families. Maximum likelihood genealogies of TrySGT1 (A); TryDYX1C1 (B); ATOM69 (C) and HSP20 (D). ML trees inferred using whole predicted amino acid sequences and models were automatic selected by AIC criteria using PROTGAMMAAUTO option. Branch are colored according to clades and lineages as fallowed: gray, free-living bodonids; skyblue, trypanosome Aquatic clade; purple, *T*. *brucei c*lade; forest-green, crocodilian clade; magenta, *T*. *theileri*/*T*. *cyclops* clade; red, *T*. *lewisi*/*T*. *cruzi* clade; green, *Phytomonas*; blue, *Herpetomonas*; yellow, *Angomonas*; orange, *Strigomonas*; olive-green, *Crithidia*/*Leptomonas;* Cyan, Leishmaniinae. Numbers on branches represent bootstrap support estimated with 500 pseudoreplicates.

Phylogenetic trees were inferred from orthologs of each ACD-like family identified in the trypanosomatid genomes. Sequences of bodonids and parabonids were positioned basal to the clade comprising all trypanosomatids. In all analyses, species of the same genus (and isolates of the same species) clustered in monophyletic assemblages. The best resolved phylogenetic relationships were obtained using *Try*HSP20, *Try*ATOM69, *Try*SGT1 and *Try*DYX1C1 (Fig 9), in agreement with traditional phylogeny (SSU rRNA and gGAPDH) of Trypanosomatidae [1–3]. The analysis of kinetoplastid-specific *TryACDp* supports similar topology despite low support values in many branches, whereas more considerable inconsistencies were observed in genealogies of *Try*p23A, *Try*p23B, and both paralogs of *Try*NudC (S4 Fig).

Comparison of phylogenies derived from ACDp, the SSU rRNA or gGAPDH (glycosomal glyceraldehyde 3-phosphate dehydrogenase) revealed remarkable congruence on branching patterns, with ACD-like genes supporting two major phylogenetic lineages. One lineage comprises *Trypanosoma*, and the other harbored the species of subfamily Leishmaniinae comprising human infective and pathogenic species of *Leishmania*, and parasites predominantly of sloths of the genus *Endotrypanum*, all transmitted by sand flies, plus closely related insect trypanosomatids of the genera *Zelonia*, *Crithidia*, and *Leptomonas*. Relatives of Leishmaniinae are the species of *Herpetomonas* (monoxenous) and *Phytomonas* (plants and phytophagous hemipterans), and the more distantly related insect parasites of *Angomonas* and *Strigomonas* [2,67,68]. In agreement with the expected phylogenetic relationships inferred by conventional phylogenies based on SSU rRNA and gGAPDH [1], the most basal clade of the genus *Trypanosoma* (the aquatic clade) comprises trypanosomes from fishes, anurans and lizards (Fig 9), transmitted by both aquatic leeches and biting flies [1,69,70]. The species of trypanosomes infective to mammals were distributed in two main previously recognized phylogenetic lineages referred to as follows: clade *T*. *brucei* (Fig 9), constituted of African trypanosomes pathogenic to ungulates and transmitted by tsetse flies [1,4,71]; clade *T*. *cruzi* (Fig 9), comprising the trypanosome generalists of mammals and human infective *T*. *cruzi* (pathogenic) and *T*. *rangeli* (non-pathogenic), trypanosomes restricted to bats, and some species parasitic in rodents, monkeys, carnivores and marsupials from both the New and Old Worlds. The species nested into the clade *T*. *cruzi* are transmitted by triatomine and cimicid bugs [6,72,73]. In addition, our analyses provide additional support to other well-established phylogenetic lineages such as the clade clustering *T*. *theileri* (cosmopolitan parasites of ruminants) and *T*. *cyclops* (Asian monkeys) [1,74], and the crocodilian clade (Fig 9) formed by closely related trypanosomes from African crocodiles (transmitted by tsetse flies), and American caimans [75].

Trypanosomatid evolution is remarkable affected by events of host switching with life stages in different environments, and a range of vector-transmission strategies. These events lead to drastic changes in selective pressures, which are usually recorded in organism behavior, morphology, and genetics. To assess if specific clades evolved at different rates and could be under different selective pressures, we tested for significant positive diversifying selection on individual branches of the phylogenetic trees inferred for each ACDp. Most ACD-like genes were evidently influenced by positive selection, but some genes were more readily influenced than others. Positive selection of *Try*HSP20 and *Try*NUDC2 acted in a few branches, whereas *Try*p23A, *Try*p23B, *Try*ATOM69, *Try*NUDC1 and *Try*DYX1C1 genes diversified under selection pressure at many branches representing major lineages, genus, and species. Our analyses revealed positive selection (p>0.05) at the *Trypanosoma* branch in almost all ACDps: *Try*p23B, *Try*SGT1, *Try*DYX1C1, *Tr*yNudC1, *Tr*yNudC2, ATOM69 and ACDP. The trypanosomes of the “aquatic” clade have been submitted to strong purifying selection on ATOM69 while this process on *Try*p23B and *Try*ACDp affected only the “terrestrial” clade. Therefore, different ACDp genes evolved under strong selective pressure on different branches in *Trypanosoma*. Species of the Leishmaniinae were affected by selection pressure on *Try*p23B, *Try*SGT1, *Try*DYX1C1, *Try*NudC1, ATOM69 and *Try*ACDP. Positive selection on *Tr*yNudC2 was exclusively inferred for trypanosomes (S5 Fig). However, positive selection was supported by different ACDps at the branches of *Herpetomonas* (TryNudC1, TryDYX1C1 and ATOM69), and *Angomonas/Strigomonas* (*Tryp*23A, *Tryp*23B, *Try*NudC1 and ATOM69). In contrast, *Phytomonas* spp. shows no evidence of episodic selection pressure in any ACDp.

Our analyses suggested relevant selection pressure on the *Tryp*23A and *Try*NudC1 gene families during the transition from free living to the obligate parasitic lifestyle (S5 Fig). *Try*NudC1 but not its paralog *Try*NudC2 were strongly affected by positive selection at main branches (S5 Fig). These two paralogs also differed in transcriptomic and proteomic profiles, suggesting that after the duplication, functional divergence (neofunctionalization) may have occurred in NUDC-like proteins in the kinetoplastids. The recurrent detection of episodic selection suggested that the adaptation of trypanosomatids to dixeneous life alternating between vertebrate/plant and invertebrate hosts had impacted ACDps evolution (S5 Fig).

## Conclusions

Together, phylogeny and syntenic information, domain architecture and predicted structures clearly showing the hallmarks of ACD bearing proteins permitted the classification of ACD-like genes from 58 trypanosomatids and three bodonids in 9 orthologs groups. MDS analysis strongly supported 9 orthologous clusters, each corresponding to one ACDp family. Our analysis revealed the well known HSP20, p23 and ATOM69 in all kinetoplastid genomes. The unveiled ACDp repertoires of the kinetoplastids include families not previously characterized or even not identified before this study; i.e., *Try*NudC1, *Try*NudC2, *Try*SGT1, *Try*DYX1C1 and *Try*ACDP. Two families are so far exclusive and ubiquitous of kinetoplastids: ATOM69 and *Try*ACDP. Although transcripts were detected in all developmental stages, each *Try*ACD gene family displays species- and stage regulated expression profiles of proteins, suggesting early diversification of these proteins, shaped by strong episodic diversification pressures, to play specific roles in the adaptability of kinetoplastid through free-living and parasitic lifestyles. Our comprehensive *in silico* analyzes uncovered previously unknown families of ACDps evolutionary conserved in kinetoplastids, providing the basis for the greatest challenge of exploring their functions in further investigations. Beyond evolutionary relevance, the ACD domain architecture and structural insights provided by this study have potential applicability for selection of new targets for control of diseases caused by these parasites.

## Supporting information

S1 Table Species, strains and accession numbers of 61 kinetoplastid genomes analyzed in this study. (DOC)

S2 Table Accession numbers of sequences deposited in GenBank, predicted genes from TriTrypDB, PSSMs used in RPS-BLAST, and templates used to homology-modeling in Swiss-Model. (XLS)

S3 Table Transcriptomic and proteomic datasets examined for expression analyses of ACDp family. (XLS)

S4 Table Copy number of each ACDp family found in kinetoplastid genomic survey. (XLS)

S1 Fig Synteny data of each ACDp family locus. (DOC)

S2 Fig Schematic representation of expression data available for each ACDp family in insect and mammalian stages of *T*. *brucei*, *T*. *cruzi*, and *Leishmania* spp. (PDF)

S3 Fig Multiple sequence alignment of NudC, DYX1C1, SGT1, Tryp23A, Tryp23B and *Try*ACDp families. (PDF)

S4 Fig Genealogies of HSP20, Tryp23A, Tryp23B, TryNucC1, TryNucC2 and TryACDp families. (PDF)

S5 Fig Selection pressure analyses of orthologs from *Try*ACDp families. (PDF)
