# Supplementary material for: Genome-wide identification of evolutionarily conserved Small Heat-Shock and eight other proteins bearing α-crystallin domain-like in kinetoplastid protists
Source: PLoS One. 2018 Oct 22;13(10):e0206012. doi: 10.1371/journal.pone.0206012 (PMC6197667; doi:10.1371/journal.pone.0206012)
Supplement: S1 Table — (DOC) [file pone.0206012.s001.doc]

| **S1-Table** Trypanosomes, other trypanosomatids and free living kinetoplastids examined in this study | | |
| --- | --- | --- |
| **Species isolate** | **Host** | **Genome data origin** |
| **Bodonids** |  |  |
| *Bodo saltans* Lake Konstanz | free living | ^&^Wellcome Trust Sanger, GenBank GCA_001460835.1 |
| *Bodo* sp. ATCC 50149 | free living | **^#^**ATOL |
| *Parabodo caudatus* ATCC 30905 | free living | **^#^**ATOL |
| ***Trypanosoma* spp. of mammals** |  |  |
| *Trypanosoma cyclops* TCC052 | *Macaca ira* | **^#^**ATOL/USP |
| *Trypanosoma* livingstonei TCC1270 | *Rhinolophus landeri* | **^#^**USP |
| *Trypanosoma* wauwau TCC1873 | *Pteronotus personatus* | **^#^**USP |
| *Trypanosoma evansi* STIB 805 | *Bubalus bubalis* | ^&^TriTrypDB |
| *Trypanosoma brucei brucei* TREU927 | *Glossina pallidipes* | ^&^TriTrypDB |
| *Trypanosoma brucei gambiense* DAL972 | *Homo sapiens* | ^&^TriTrypDB |
| *Trypanosoma congolense* IL30000 | *Bos* sp. | ^&^TriTrypDB |
| *Trypanosoma vivax* Y486 | *Bos taurus* | ^&^TriTrypDB |
| *Trypanosoma theileri* TCC165 | *Bubalus bubalis* | **^#^**ATOL |
| *Trypanosoma lewisi* TCC34 | *Rattus rattus* | **^#^**ATOL |
| *Trypanosoma rangeli* AM80 | *Homo sapiens* | **^#^**ATOL |
| *Trypanosoma conorhini* TCC025E | *Rattus rattus* | **^#^**ATOL |
| *Trypanosoma dionisii* TCC211 | *Eptesicus brasiliensis* | **^#^**ATOL |
| *Trypanosoma erneyi* TCC1946 | *Mops condylurus* | **^#^**ATOL |
| *Trypanosoma cruzi cruzi* G | *Didelphis marsupialis* | **^#^**USP |
| *Trypanosoma cruzi marinkellei* B7 | *Phyllostomus discolor* | ^&^TriTrypDB |
| *Trypanosoma* noyesi TCC16 | *Macropus giganteus* | **^#^**ATOL/USP |
| ***Trypanosoma* spp. of cold-blooded vertebrates** |  |  |
| *Trypanosoma grayi* ANR4 | *Glossina palpalis* | ^&^TriTrypDB |
| *Trypanosoma* sp. TCC1307 | *Ptychadena mossambica* | **^#^**ATOL/USP |
| *Trypanosoma* sp. TCC2186 | *Leptodactylus ocellatus* | **^#^**ATOL/USP |
| *Trypanosoma ralphi* TCC1838 | *Melanosuchus niger* | **^#^**USP |
| *Trypanosoma* sp. TCC339 | *Rhinella marina* | **^#^**ATOL |
| *Trypanosoma* sp. TCC878 | *Mabuya frenata* | **^#^**USP |
| ***Leishmaniinae* spp.** |  |  |
| *Leptomonas pyrrhocoris* TCC 2441 | *Dysdercus* sp. | **^#^**ATOL |
| *Zelonia costaricensis* TCC169E | *Zelinae* sp. | **^#^**ATOL |
| *Zelonia* sp. TCC2547 | *Ricolla* sp. | **^#^**USP |
| *Crithidia acantocephali* TCC037E | *Acanthocephala femorata* | **^#^**ATOL |
| *Crithidia luciliae thermophila* TCC050E | *Zelus leucogrammus* | **^#^**ATOL |
| *Crithidia fasciculata* Cf-CI | *NA* | ^&^TriTrypDB |
| *Endotrypanum monterogeii* LV88 | *Choloepus hoffmani* | ^&^TriTrypDB |
| *Endotrypanum schaudinni* TCC224 | *Choloepus hoffmani* | **^#^**ATOL |
| *Leishmania (M.) enrietti* LEM3045 | *Cavia porcellus* | ^&^TriTrypDB |
| *Leishmania (M.) martiniquensis* MARLEM2494 | *Homo sapiens* | ^&^TriTrypDB |
| *Leishmania (V.) braziliensis* MHOMBR75M2904 | *Homo sapiens* | ^&^TriTrypDB |
| *Leishmania (V.) panamensis* MHOM/COL/81/L13 | *Homo sapiens* | ^&^TriTrypDB |
| *Leishmania (L.) aethiopica* L147 | *Homo sapiens* | ^&^TriTrypDB |
| *Leishmania (L.) amazonensis* MHOM/BR/71973/M2269 | *Homo sapiens* | ^&^TriTrypDB |
| *Leishmania (L.) arabica* LEM1108 | *Homo sapiens* | ^&^TriTrypDB |
| *Leishmania (L.) donovani* BPK282A1 | *Homo sapiens* | ^&^TriTrypDB |
| *Leishmania (L.) gerbilli* LEM452 | *Homo sapiens* | ^&^TriTrypDB |
| *Leishmania (L.) infantum* JPCM5 | *Homo sapiens* | ^&^TriTrypDB |
| *Leishmania (L.) major* Friedlin | *Homo sapiens* | ^&^TriTrypDB |
| *Leishmania (L.) mexicana* MHOM/GT/2001/U1103 | *Homo sapiens* | ^&^TriTrypDB |
| *Leishmania (L.) tropica* L590 | *Homo sapiens* | ^&^TriTrypDB |
| *Leishmania (L.) turanica* LEM423 | *Homo sapiens* | ^&^TriTrypDB |
| *Leishmania (S.) tarentolae* ParrotTarII | *Tarentola mauritanica* | ^&^TriTrypDB |
| **Plant pathogen *Phytomonas*** |  |  |
| *Phytomonas serpens* P9 | *Lycopersicon esculentum* | ^&^GenBank AIHY00000000.1 |
| *Phytomonas* sp. HART1 | *Cocos nucifera* | ^&^GenBank CAVR000000000.2 |
| *Phytomonas* sp. EM1 | *Euphorbia* sp. | ^&^GenBank CAVQ000000000.1 |
| *Phytomonas dolleti* TCC 418 | *Pachycoris torridus* | **^#^**ATOL |
| *Phytomonas* sp. TCC066E | *Jatropha macrantha* | **^#^**ATOL |
| ***Herpetomonas*** |  |  |
| *Herpetomonas wanderleyi* TCC1982 | *Cochliomy macellaria* | **^#^**ATOL/USP |
| *Herpetomonas muscarum* TCC001E | *musca dosmestica* | ^&^ATOL & GenBank AUXJ01000000.1 |
| **Symbiont-harboring trypanosomatids** |  |  |
| *Angomonas deanei* TCC036E | *Zelus leucogrammus* | ^&^ATOL & GenBank AUXM01000000.1 |
| *Angomonas desouzai* TCC079E | *Ornidia obesa* | ^&^ATOL & GenBank AUXL01000000.1 |
| *Strigomonas culicis* TCC012E | *Aedes vexans* | ^&^ATOL & GenBank AUXH01000000.1 |
| *Strigomonas galati* TCC219 | *Lutzomya almerioi* | ^&^ATOL & GenBank AUXN00000000.1 |
| *Strigomonas oncopelti* TCC290E | *Oncopeltus fasciatus* | ^&^ATOL & GenBank AUXK01000000.1 |

TriTrypDB - http://tritrypdb.org/tritrypdb/

GenBank - https://www.ncbi.nlm.nih.gov/genbank/

ATOL: Assembling the Tree of Life (NSF-USA);

USP: Department of Parasitology, University of São Paulo, USP.

& publicly available genomes; # access to these ongoing genomes can be obtained by contacting the corresponding author (Marta M.G. Teixeira -mmgteix@icb.usp.br).
