# Supplementary figures and images for: Genome-wide identification of evolutionarily conserved Small Heat-Shock and eight other proteins bearing α-crystallin domain-like in kinetoplastid protists
Source: PLoS One. 2018 Oct 22;13(10):e0206012. doi: 10.1371/journal.pone.0206012 (PMC6197667; doi:10.1371/journal.pone.0206012)

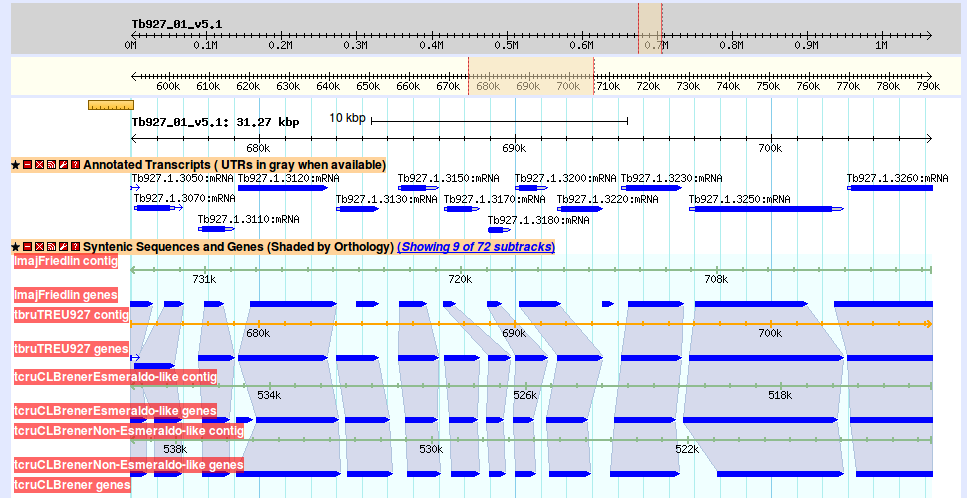


TrySGT1

HSP20


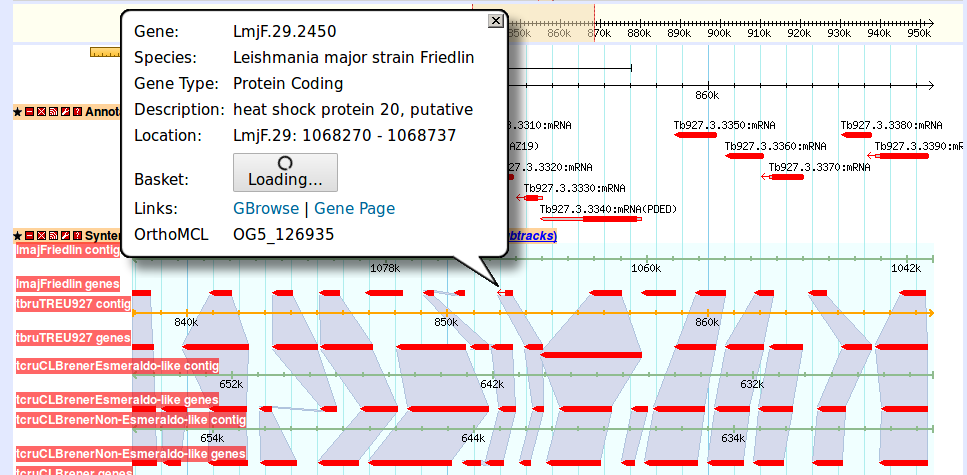


TryNudC2


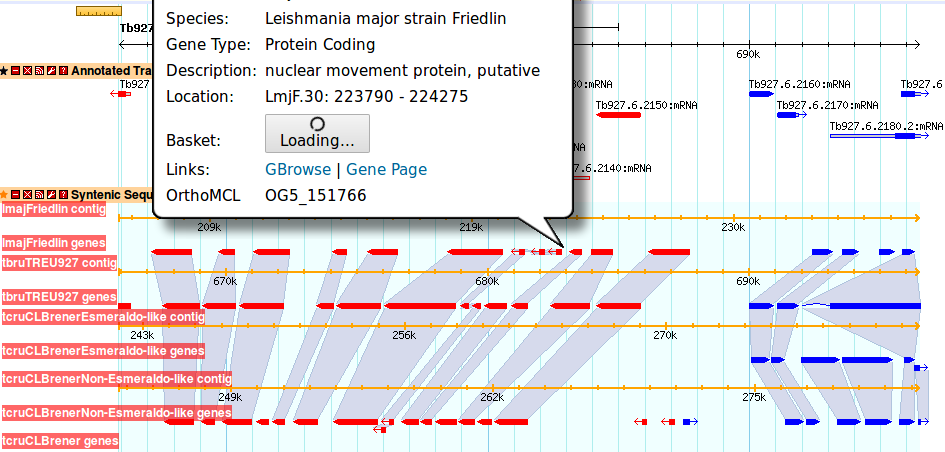


TryNudC1


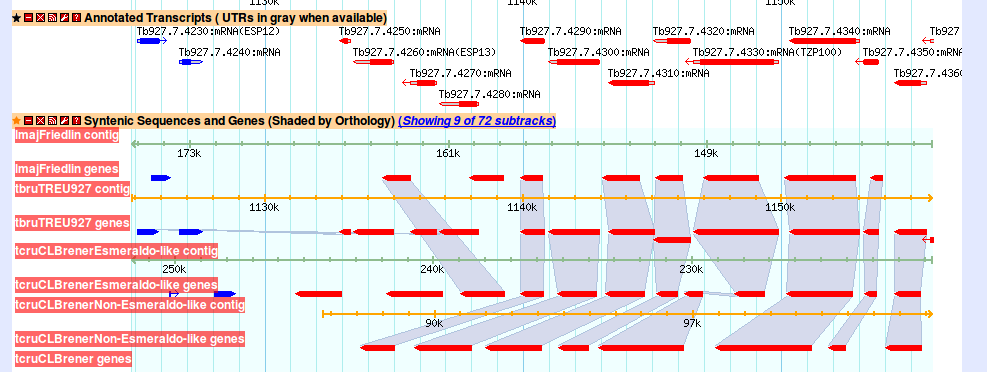


p23A


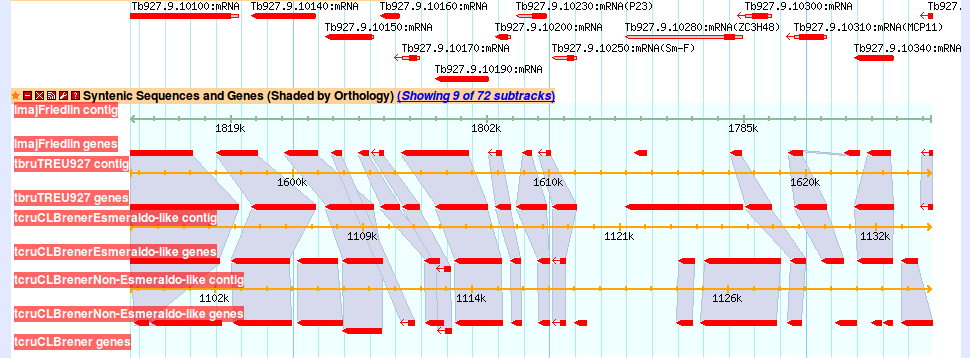


p23b


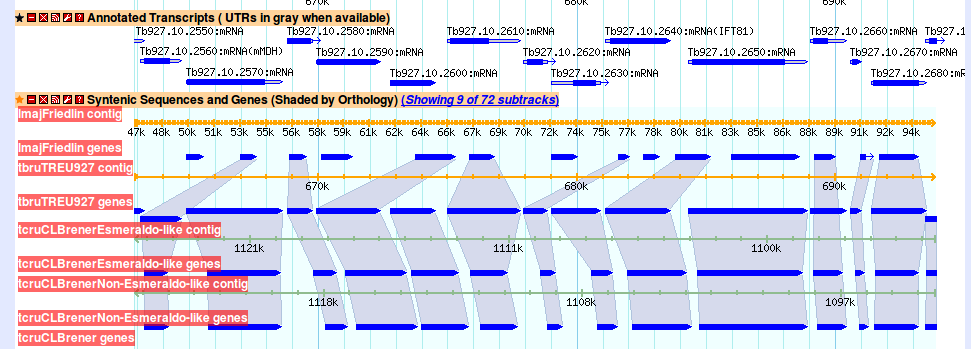


ATOM69


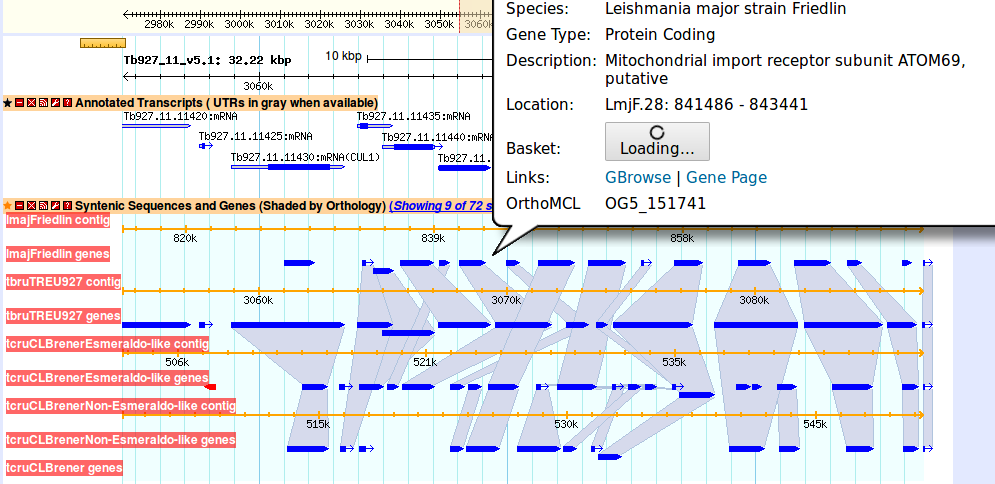


ACDp


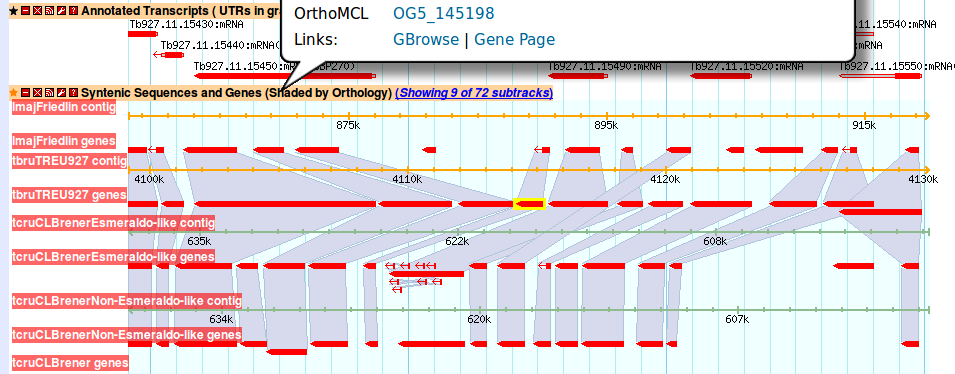


ACDp – T. vivax and T. brucei brucei synteny


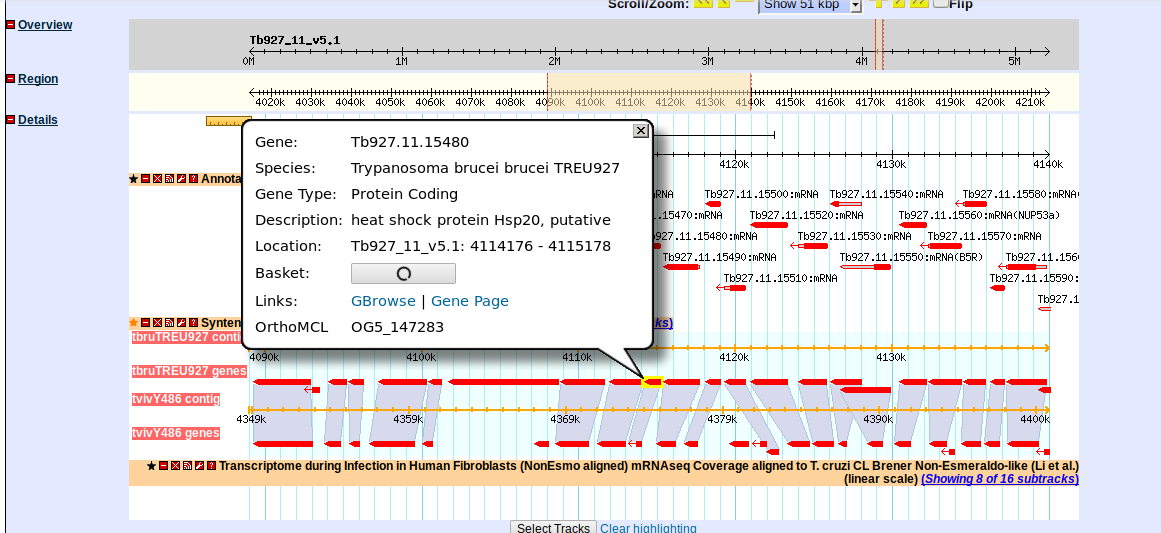


TryDYX1C1


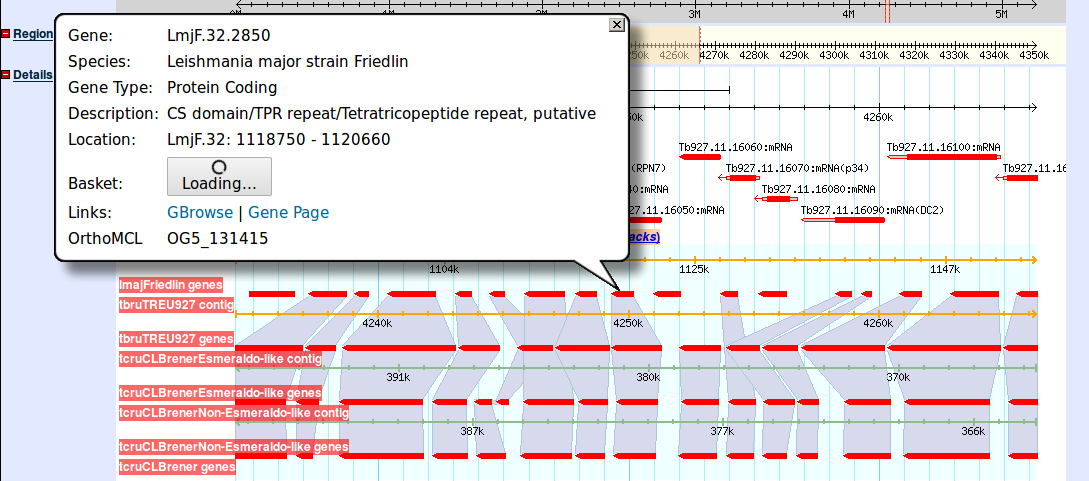

Supplement: S1 Fig — (DOC) [file pone.0206012.s005.doc]

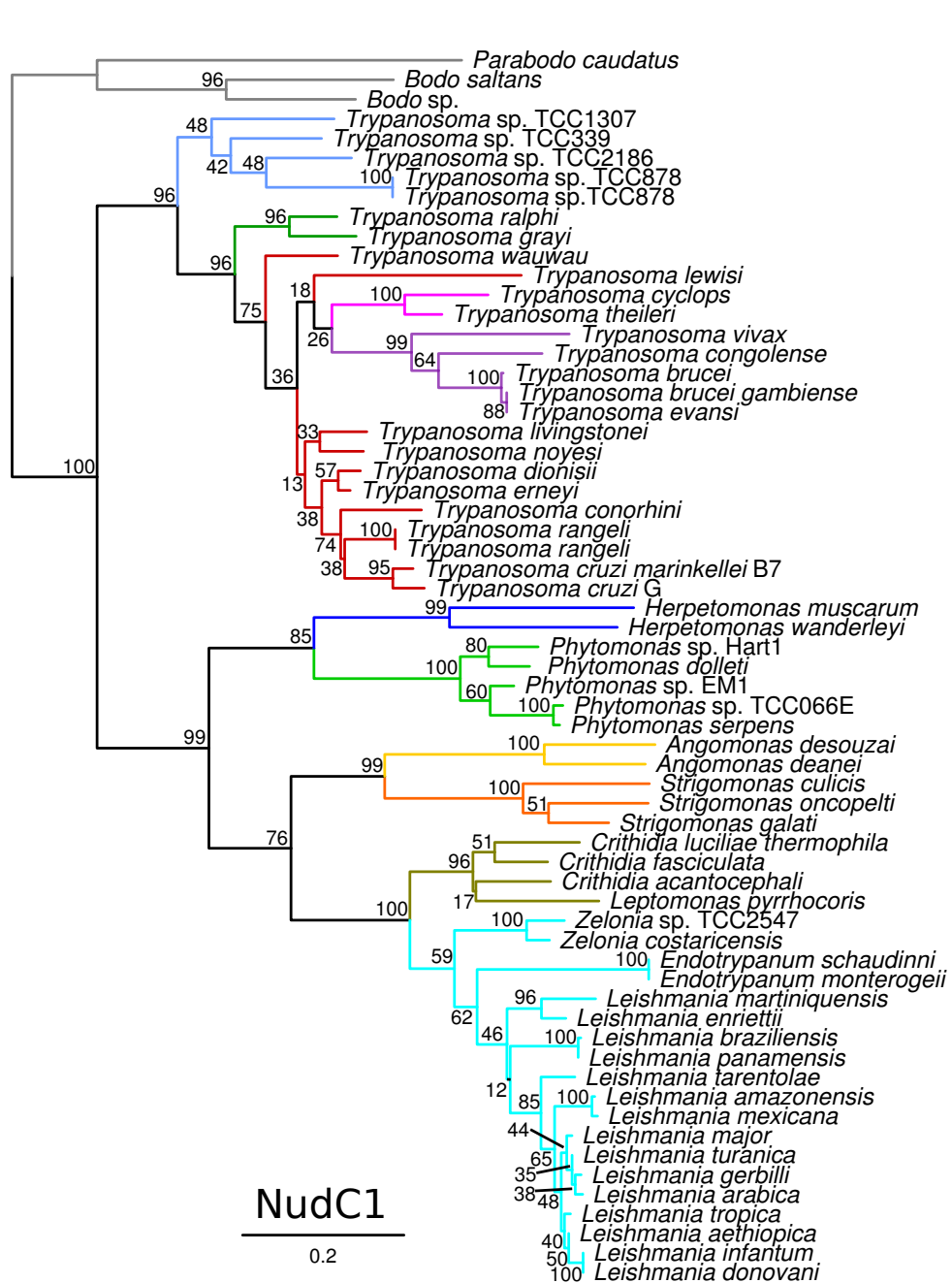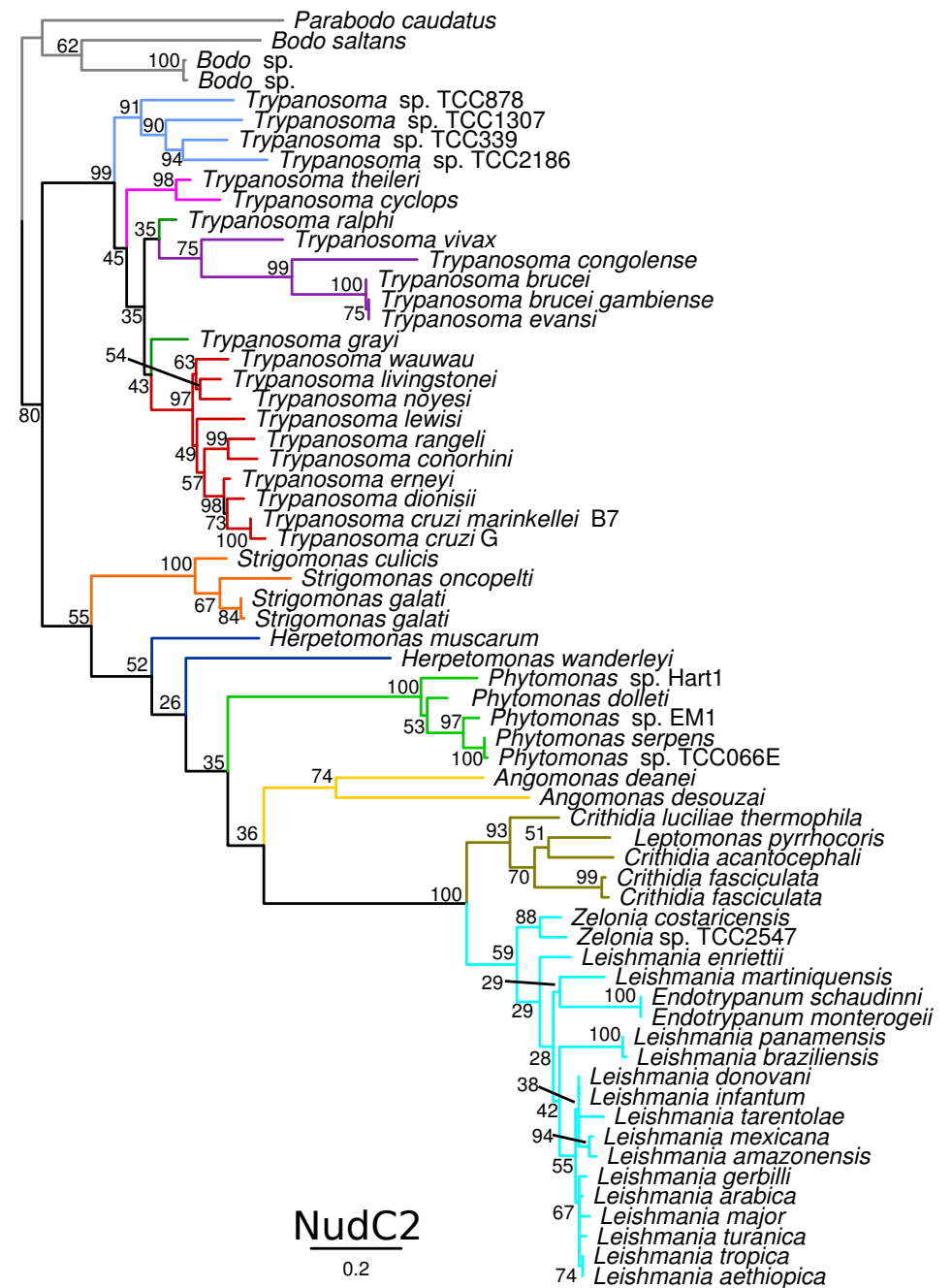

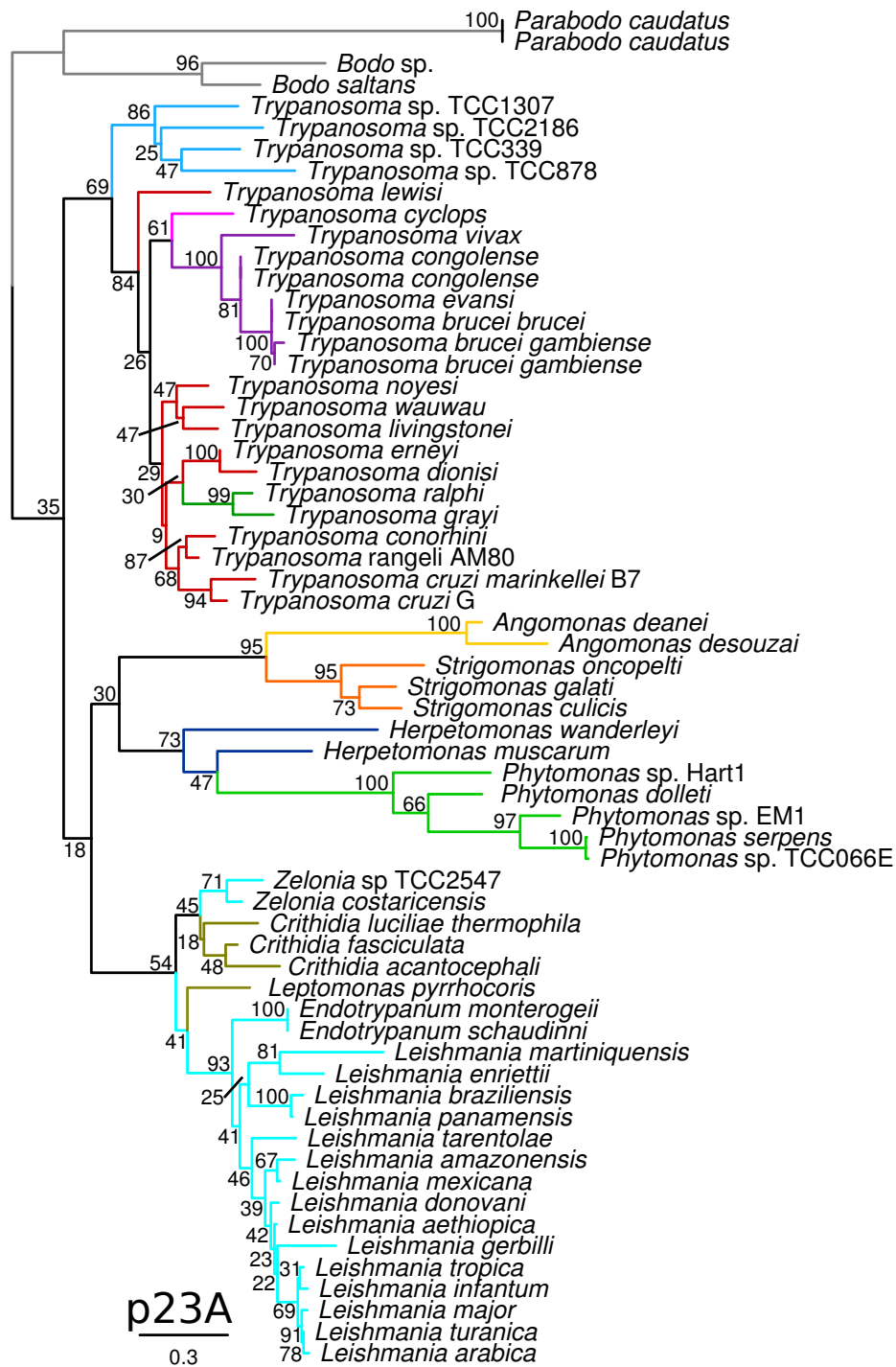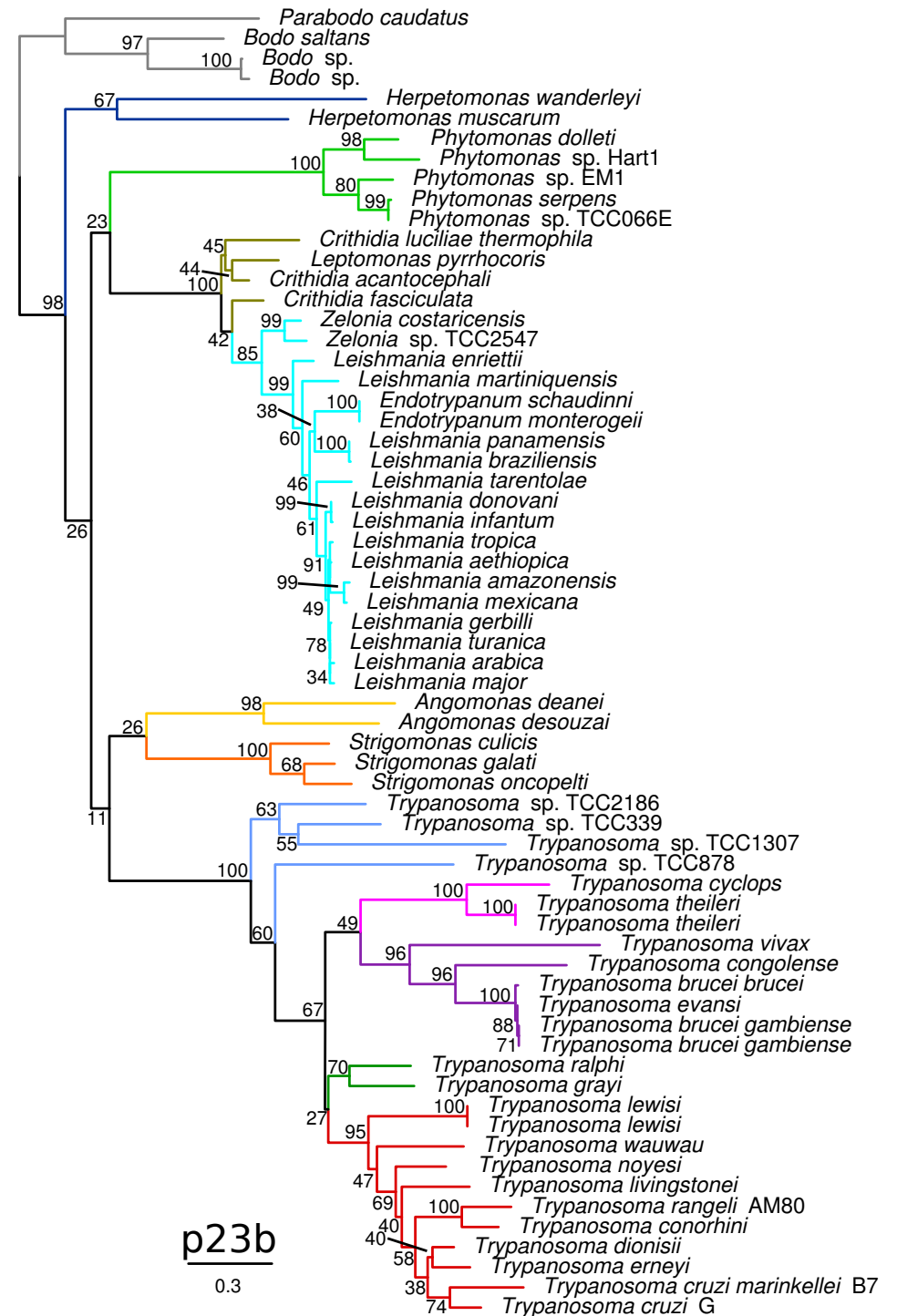

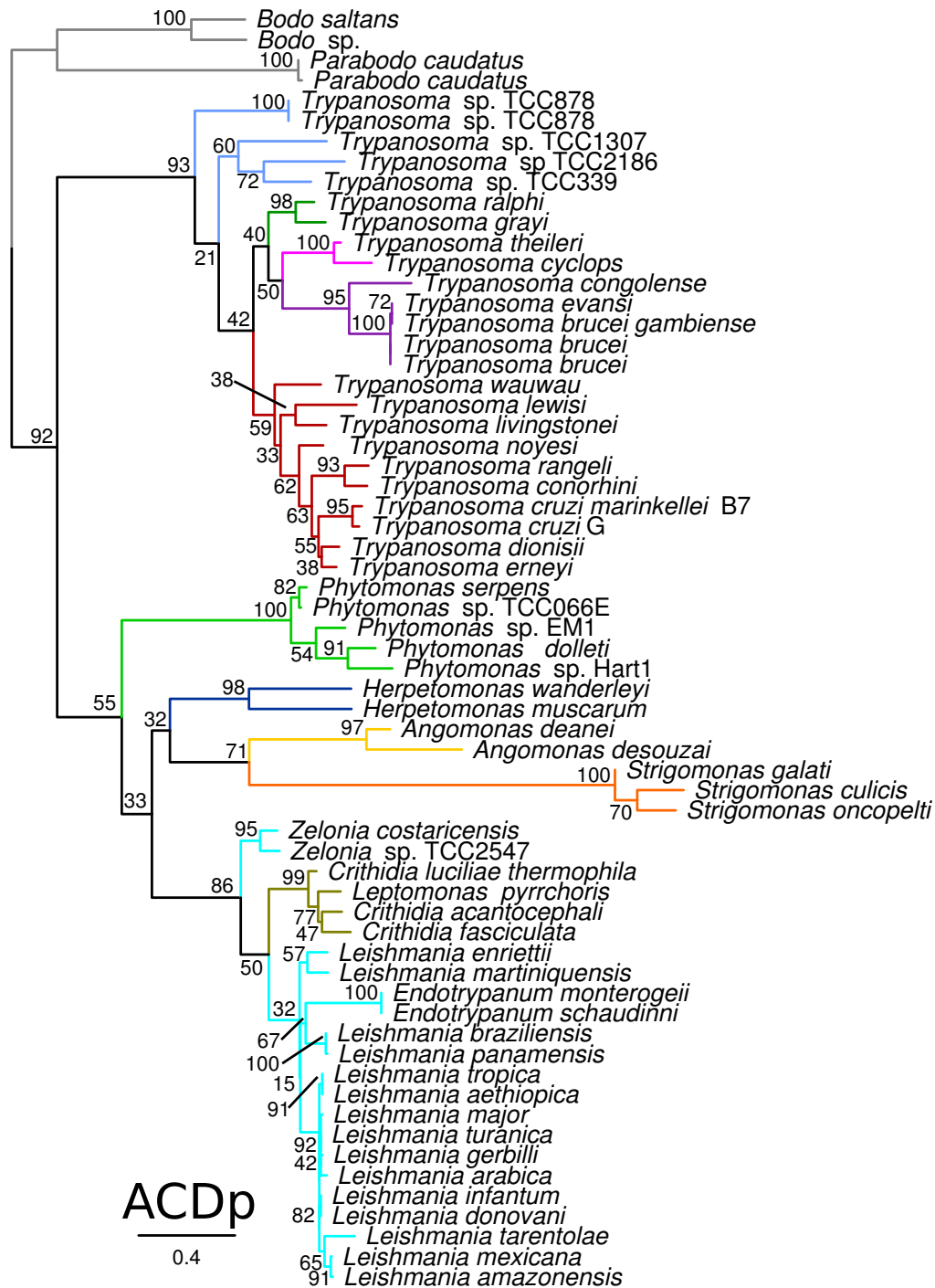

Supplement: S4 Fig — (PDF) [file pone.0206012.s008.pdf]
