# Supplementary material for: Genome-wide identification of evolutionarily conserved Small Heat-Shock and eight other proteins bearing α-crystallin domain-like in kinetoplastid protists
Source: PLoS One. 2018 Oct 22;13(10):e0206012. doi: 10.1371/journal.pone.0206012 (PMC6197667; doi:10.1371/journal.pone.0206012)
Supplement: S2 Fig — (PDF) [file pone.0206012.s006.pdf]

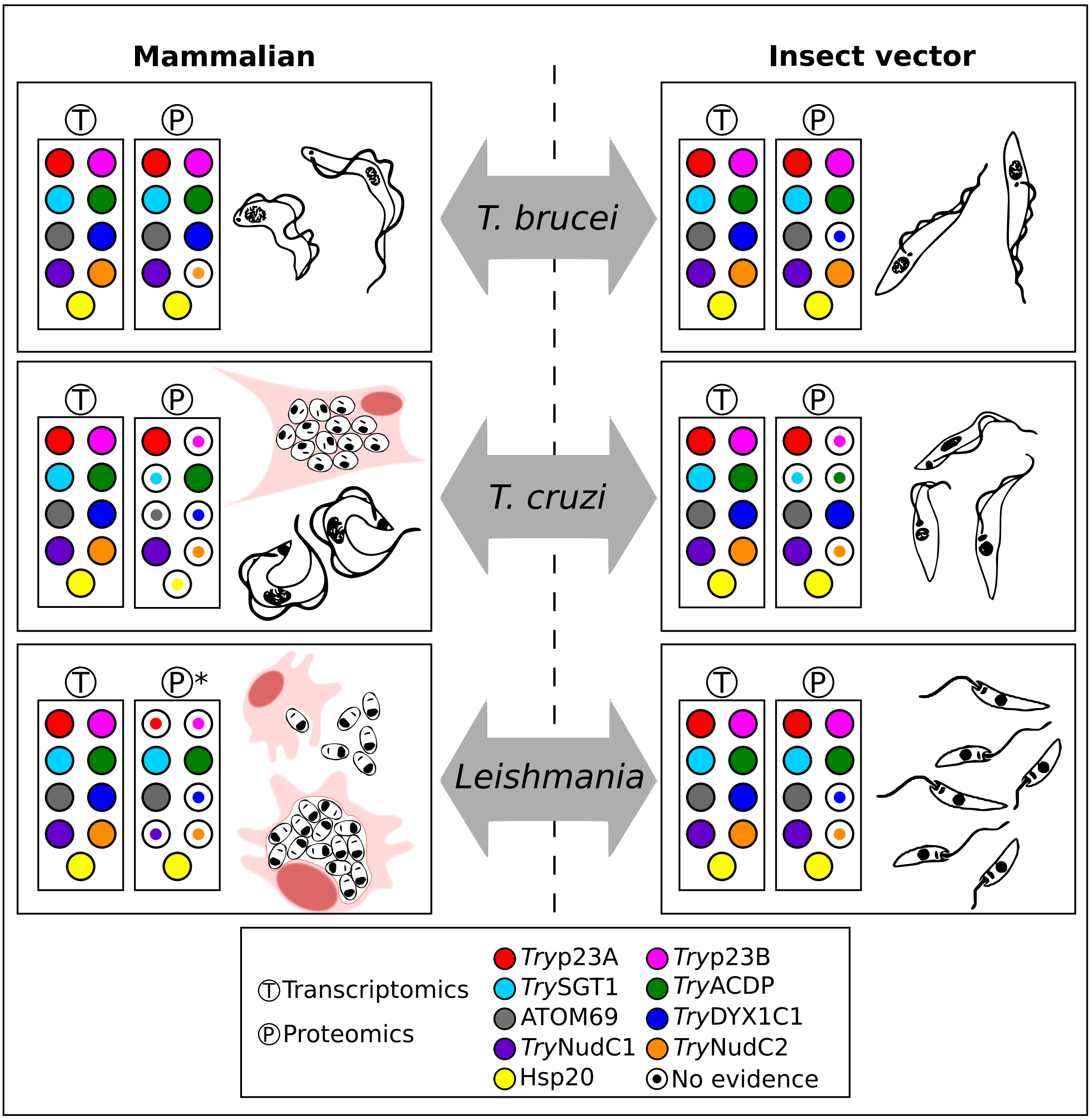

Brandão et al., 1997; Porcel et al., 2000; Almeida et al., 2004; Atwood et al., 2005; Cordero et al., 2009; Jensen et al., 2009; Minning et al., 2009; Nett et al., 2009; Panigrahi et al., 2009; Emmer et al., 2011; Bayona et al., 2011; Siegel et al., 2010; Paape et al., 2010; Oberholzer et al., 2011; Ulrich et al., 2011; dos Santos et al., 2012; Lott et al., 2013; Subota et al., 2014; Pawar et al., 2014; Urbaniak et al., 2012; Smircich et al., 2015; Li et al., 2016
