# Supplementary material for: Genome-wide identification of evolutionarily conserved Small Heat-Shock and eight other proteins bearing α-crystallin domain-like in kinetoplastid protists
Source: PLoS One. 2018 Oct 22;13(10):e0206012. doi: 10.1371/journal.pone.0206012 (PMC6197667; doi:10.1371/journal.pone.0206012)
Supplement: S3 Fig — (PDF) [file pone.0206012.s007.pdf]

NudC1 homologs

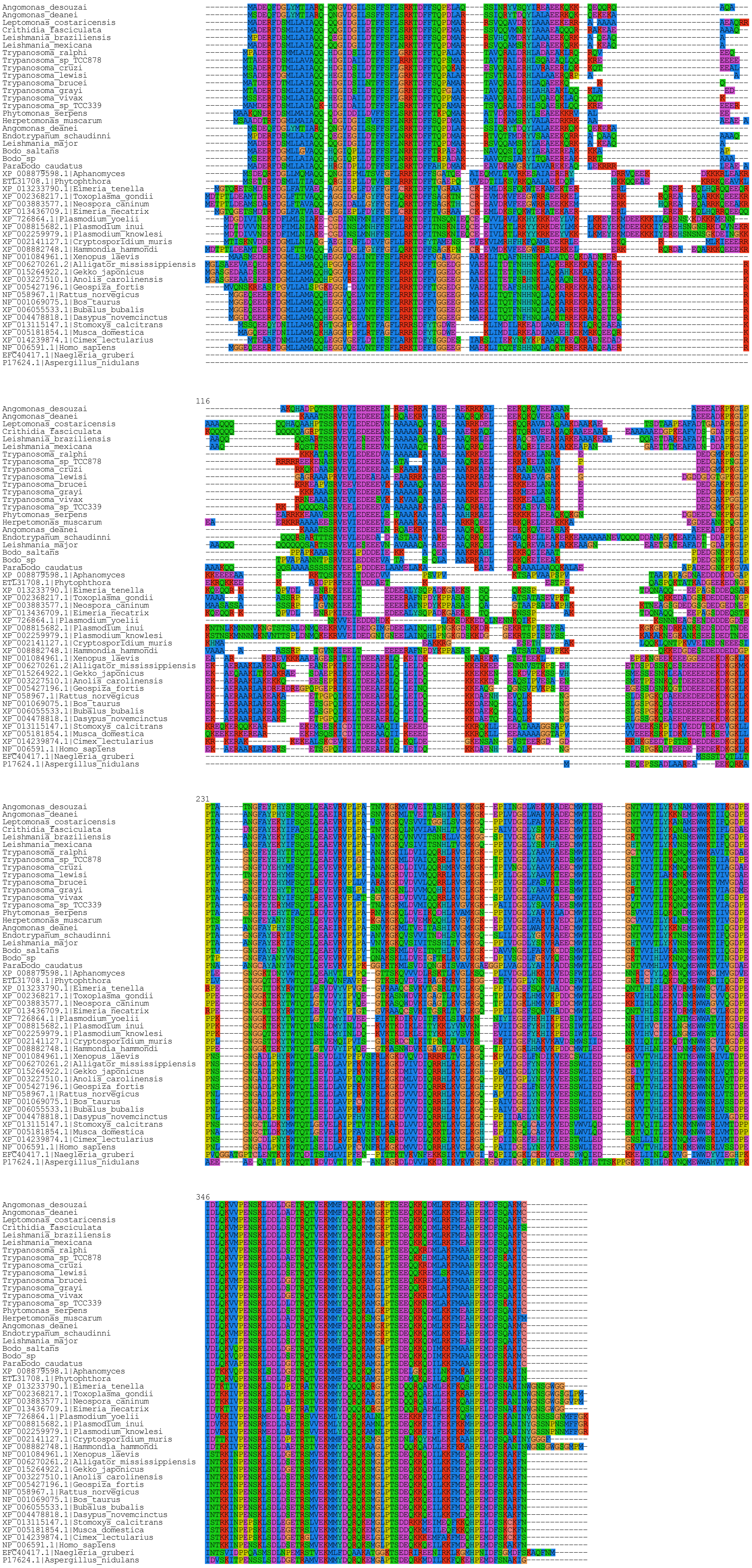

TryNudC2

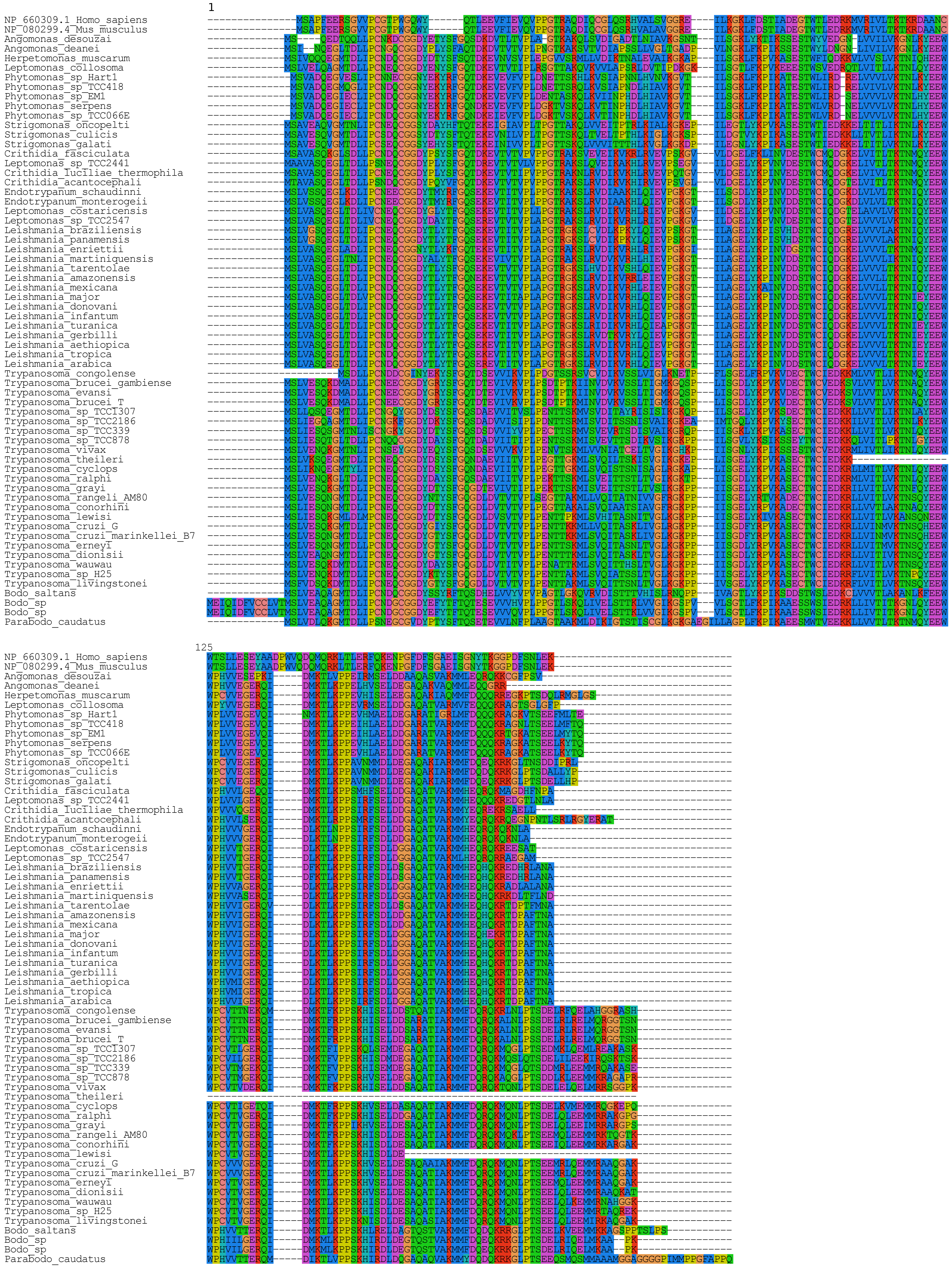
1
125
NP_660309.1_Homo_sapiens
NP_080299.4_Mus_musculus
Angomonas_desouzai
Angomonas_deanei
Herpetomonas_muscarum
Leptomonas_collosoma
Phytomonas_sp_Hart1
Phytomonas_sp_TCC418
Phytomonas_sp_EM1
Phytomonas_serpens
Phytomonas_sp_TCC066E
Strigomonas_oncopelti
Strigomonas_culicis
Strigomonas_galati
Crithidia_fasciculata
Leptomonas_sp_TCC2441
Crithidia_lucilliae_thermophila
Crithidia_acantocephali
Endotrypanum_schaudinni
Endotrypanum_monterogeii
Leptomonas_costaricensis
Leptomonas_sp_TCC2547
Leishmania_braziliensis
Leishmania_panamensis
Leishmania_enriettii
Leishmania_martiniquensis
Leishmania_tarentolae
Leishmania_amazonensis
Leishmania_mexicana
Leishmania_major
Leishmania_donovani
Leishmania_infantum
Leishmania_turanica
Leishmania_gerbilli
Leishmania_aethiopica
Leishmania_tropica
Leishmania_arabica
Trypanosoma_congolense
Trypanosoma_brucei_gambiense
Trypanosoma_evansi
Trypanosoma_brucei_T
Trypanosoma_sp_TCC1307
Trypanosoma_sp_TCC2186
Trypanosoma_sp_TCC339
Trypanosoma_sp_TCC878
Trypanosoma_vivax
Trypanosoma_theileri
Trypanosoma_cyclops
Trypanosoma_ralphi
Trypanosoma_grayi
Trypanosoma_rangeli_AM80
Trypanosoma_conorhini
Trypanosoma_lewisi
Trypanosoma_cruzi_G
Trypanosoma_cruzi_marinkellei_B7
Trypanosoma_erneyi
Trypanosoma_dionisii
Trypanosoma_wauwau
Trypanosoma_sp_H25
Trypanosoma_livingstonei
Bodo_saltans
Bodo_sp
Bodo_sp
Parabodo_caudatus

TryACDp

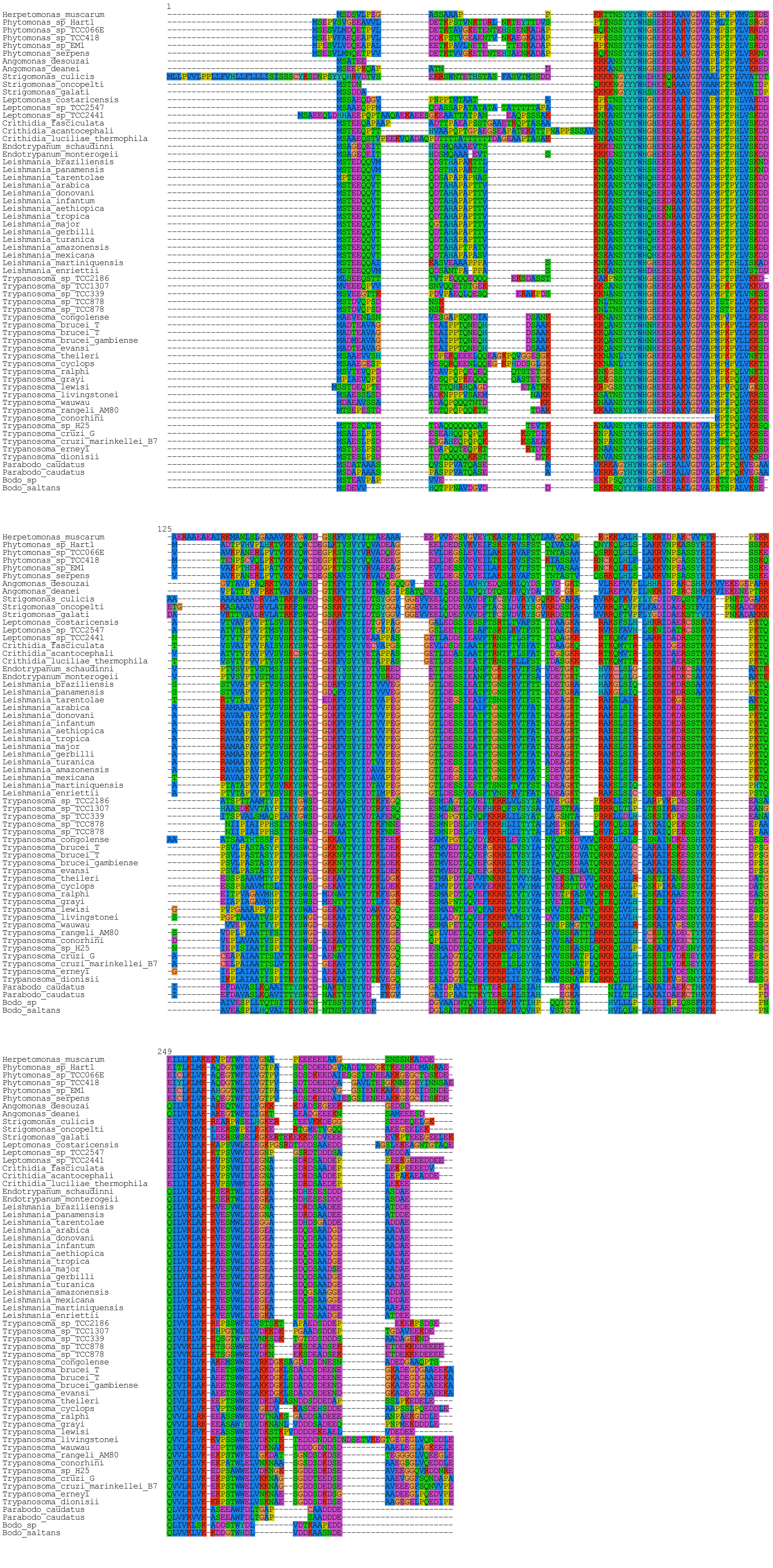

TryDYX1C1

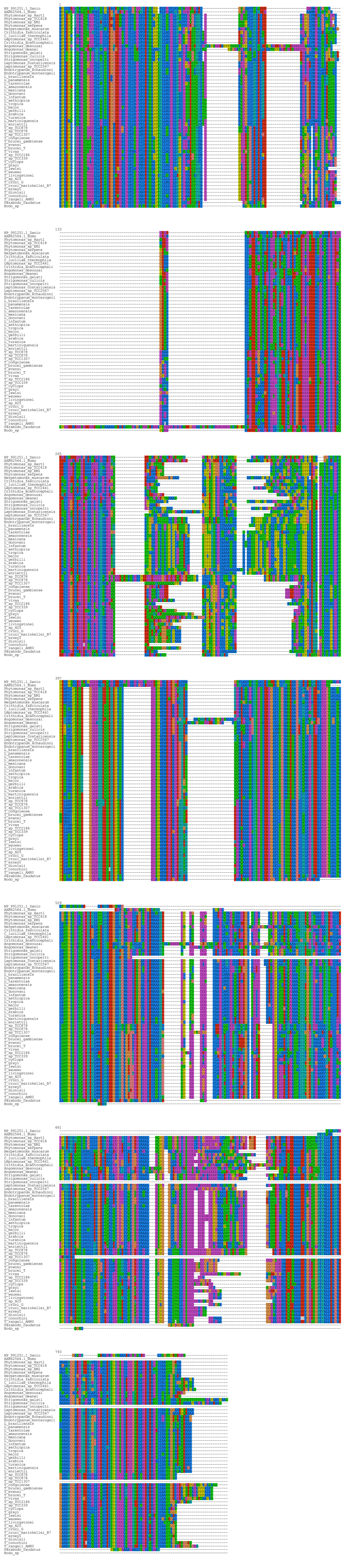

TrySGT1

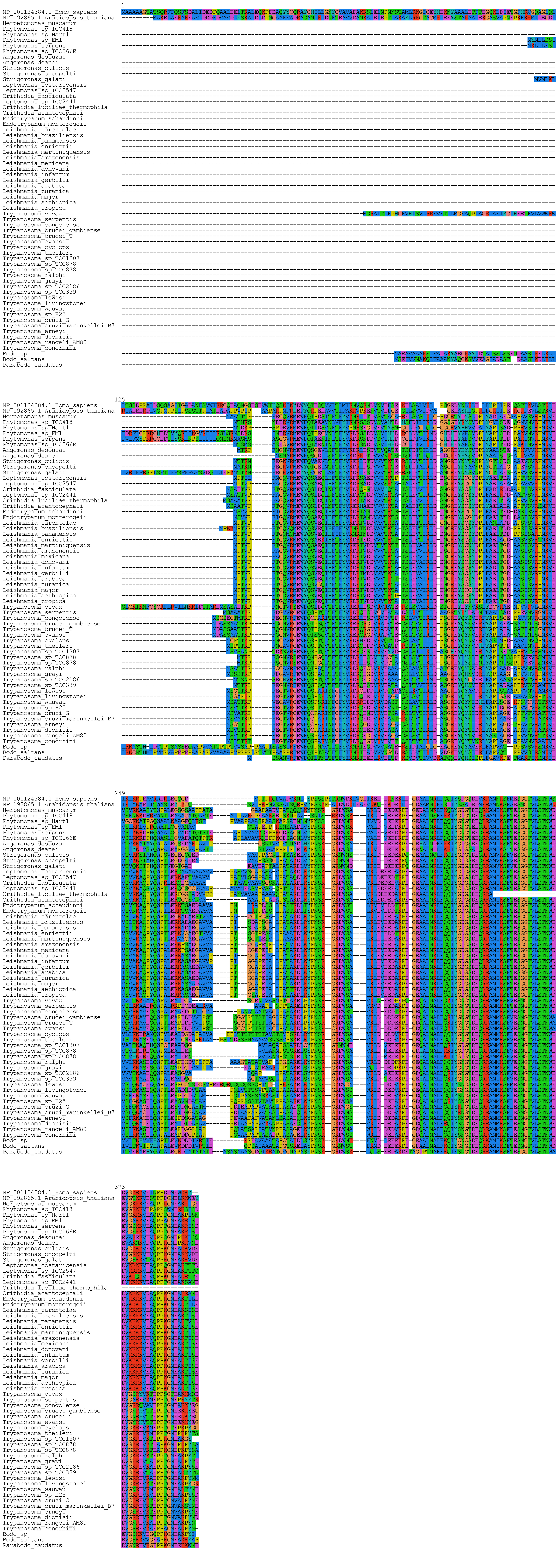

Tryp23A

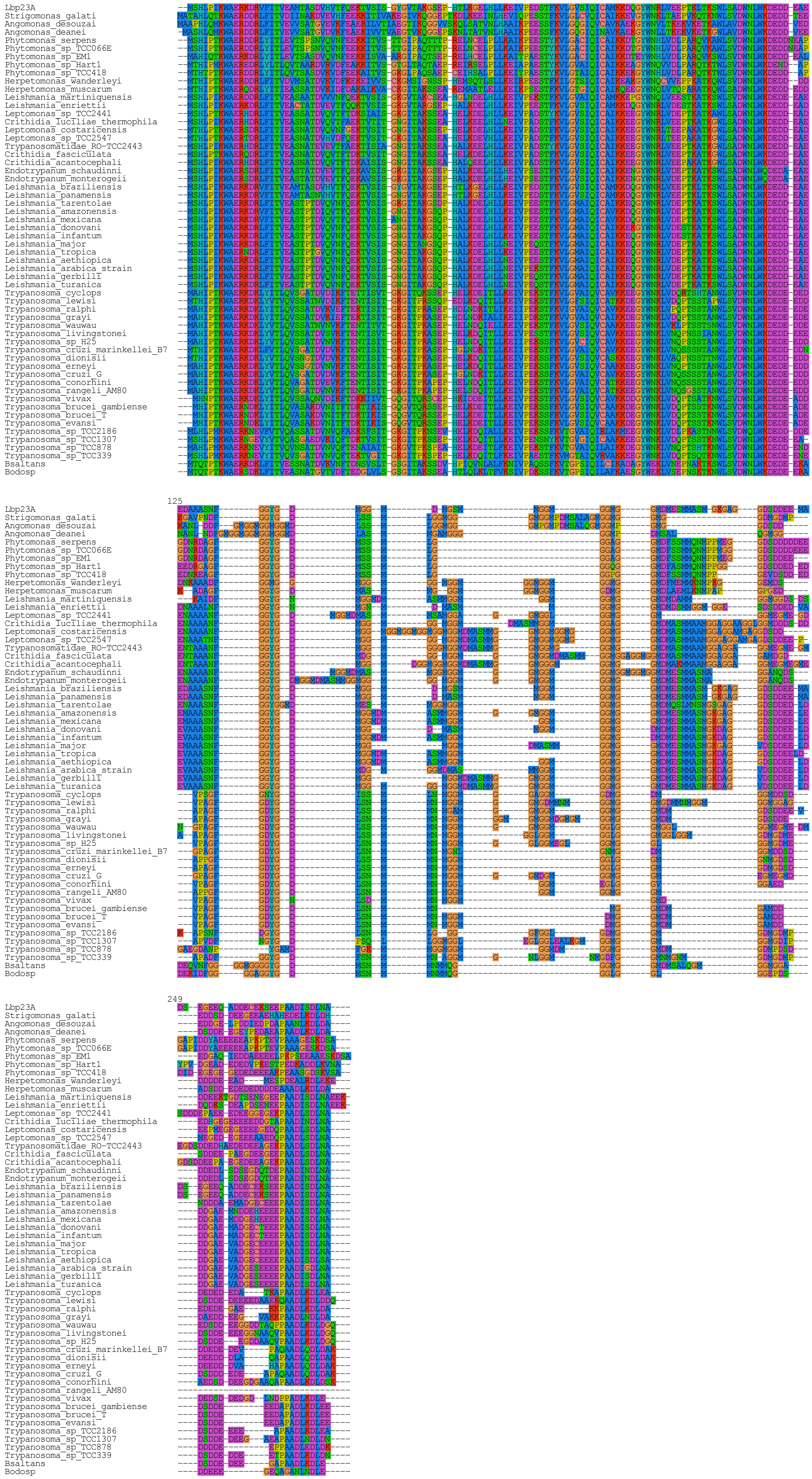
Lbp23A
Strigomonas_galati
Angomonas_desouzai
Angomonas_deanei
Phytomonas_serpens
Phytomonas_sp_TCC066E
Phytomonas_sp_EM1
Phytomonas_sp_Hart1
Phytomonas_sp_TCC418
Herpetomonas_wanderleyi
Herpetomonas_muscarum
Leishmania_martiniquensis
Leishmania_enriettii
Leptomonas_sp_TCC2441
Crithidia_luciliae_thermophila
Leptomonas_costaricensis
Leptomonas_sp_TCC2547
Trypanosomatidae_RO-TCC2443
Crithidia_fasciculata
Crithidia_acantocephali
Endotrypanum_schaudinni
Endotrypanum_monterogeii
Leishmania_braziliensis
Leishmania_panamensis
Leishmania_tarentolae
Leishmania_amazonensis
Leishmania_mexicana
Leishmania_donovani
Leishmania_infantum
Leishmania_major
Leishmania_tropica
Leishmania_aethiopica
Leishmania_arabica_strain
Leishmania_gerbilli
Leishmania_turanica
Trypanosoma_cyclops
Trypanosoma_lewisi
Trypanosoma_ralphi
Trypanosoma_grayi
Trypanosoma_wauwau
Trypanosoma_livingstonei
Trypanosoma_sp_H25
Trypanosoma_cruzi_marinkellei_B7
Trypanosoma_dionisii
Trypanosoma_erneyi
Trypanosoma_cruzi_G
Trypanosoma_conorhini
Trypanosoma_rangeli_AM80
Trypanosoma_vivax
Trypanosoma_brucei_gambiense
Trypanosoma_brucei_T
Trypanosoma_evansi
Trypanosoma_sp_TCC2186
Trypanosoma_sp_TCC1307
Trypanosoma_sp_TCC878
Trypanosoma_sp_TCC339
Bsaltans
Bodosp
125
249

Tryp23B

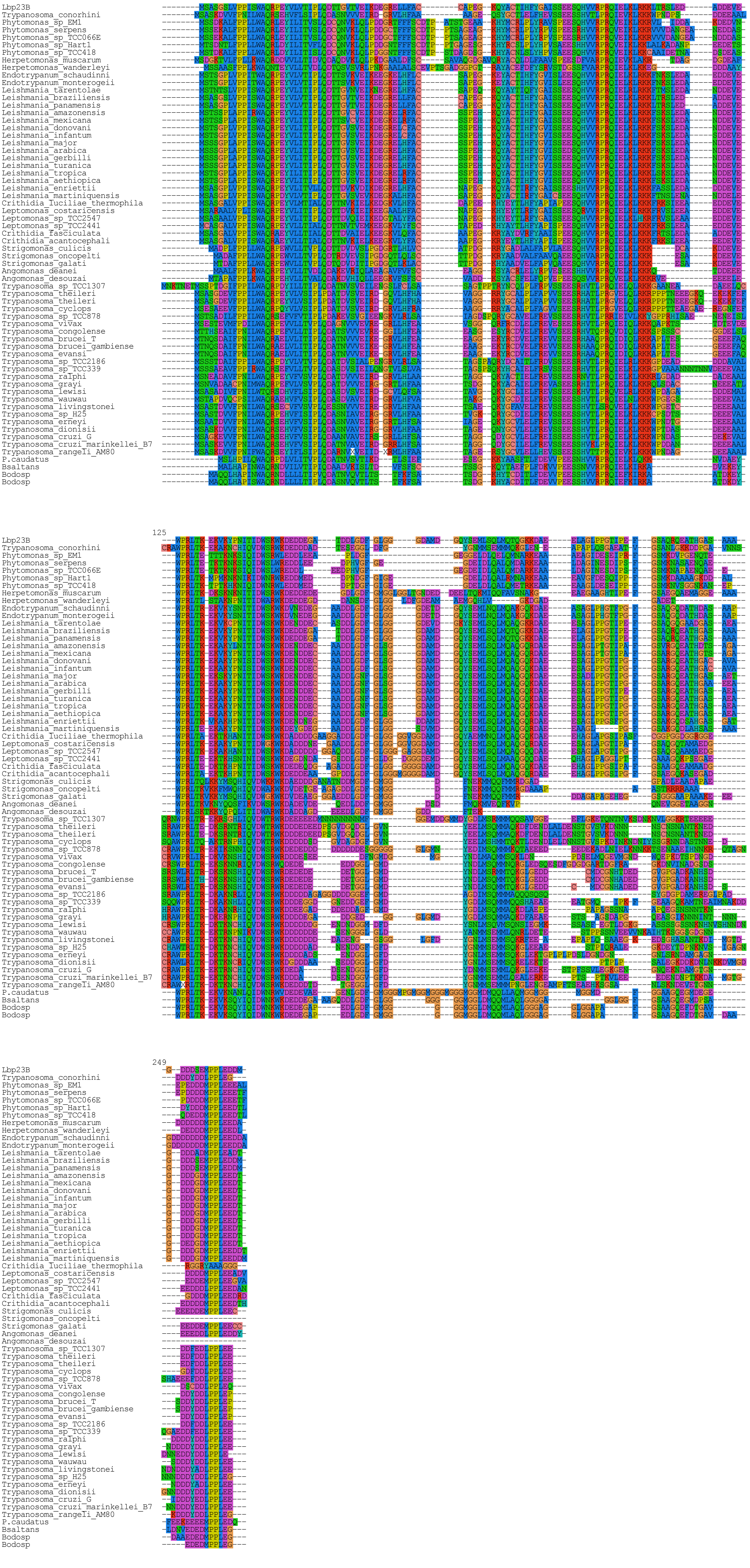
Lbp23B
Trypanosoma_conorhini
Phytomonas_sp_EM1
Phytomonas_serpens
Phytomonas_sp_TCC066E
Phytomonas_sp_Hart1
Phytomonas_sp_TCC418
Herpetomonas_muscarum
Herpetomonas_wanderleyi
Endotrypanum_schaudinni
Endotrypanum_monterogeii
Leishmania_tarentolae
Leishmania_braziliensis
Leishmania_panamensis
Leishmania_amazonensis
Leishmania_mexicana
Leishmania_donovani
Leishmania_infantum
Leishmania_major
Leishmania_arabica
Leishmania_gerbilli
Leishmania_turanica
Leishmania_tropica
Leishmania_aethiopica
Leishmania_enriettii
Leishmania_martiniquensis
Crithidia_luciliae_thermophila
Leptomonas_costaricensis
Leptomonas_sp_TCC2547
Leptomonas_sp_TCC2441
Crithidia_fasciculata
Crithidia_acantocephali
Strigomonas_culicis
Strigomonas_oncopelti
Strigomonas_galati
Angomonas_deanei
Angomonas_desouzai
Trypanosoma_sp_TCC1307
Trypanosoma_theileri
Trypanosoma_theileri
Trypanosoma_cyclops
Trypanosoma_sp_TCC878
Trypanosoma_vivax
Trypanosoma_congolense
Trypanosoma_brucei_T
Trypanosoma_brucei_gambiense
Trypanosoma_evansi
Trypanosoma_sp_TCC2186
Trypanosoma_sp_TCC339
Trypanosoma_ralphi
Trypanosoma_grayi
Trypanosoma_lewisi
Trypanosoma_wauwau
Trypanosoma_livingstonei
Trypanosoma_sp_H25
Trypanosoma_erneyi
Trypanosoma_dionisii
Trypanosoma_cruzi_G
Trypanosoma_cruzi_marinkellei_B7
Trypanosoma_rangeli_AM80
P.caudatus
Bsaltans
Bodosp
Bodosp
125
249

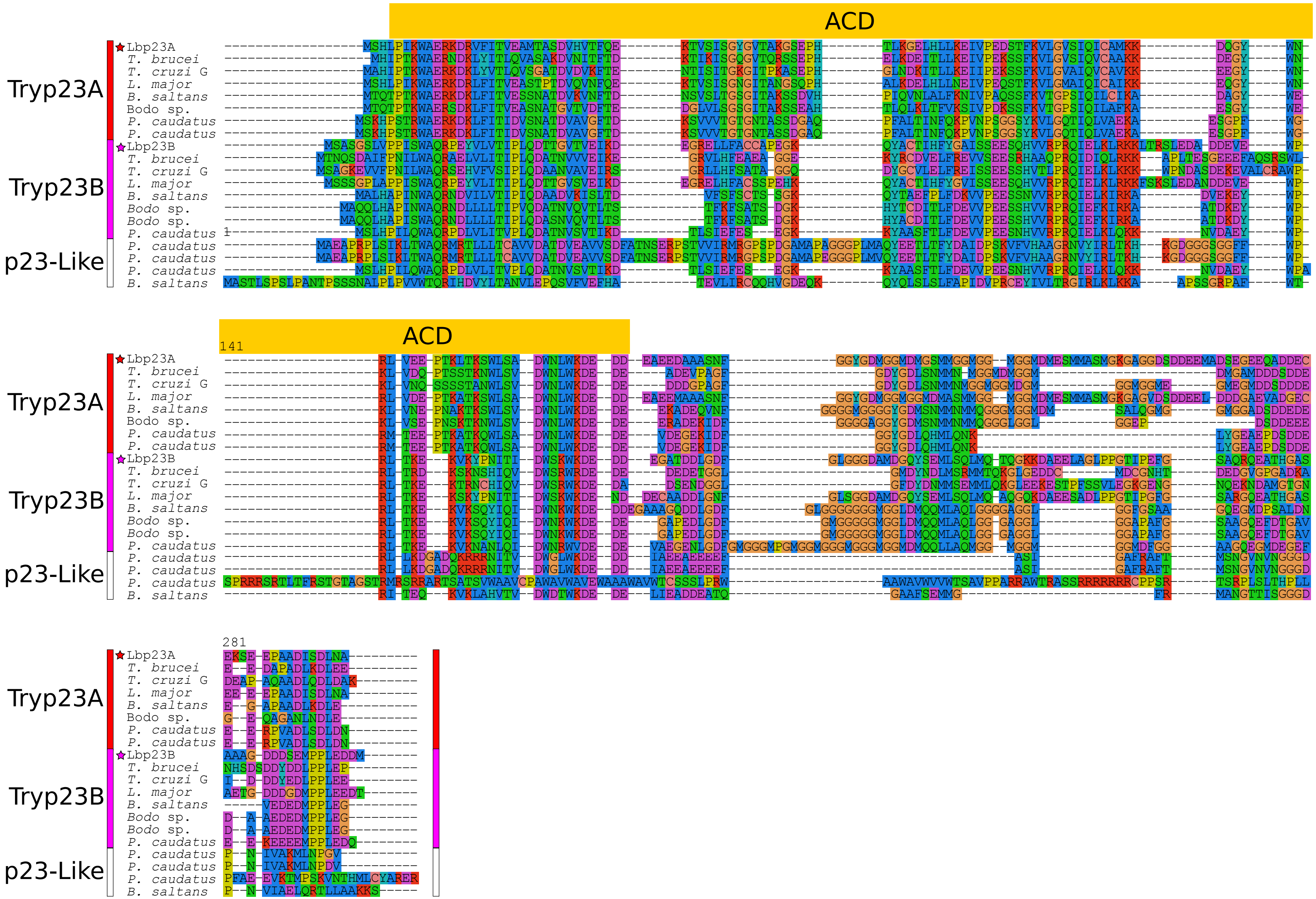
ACD
Tryp23A
Tryp23B
p23-Like
Lbp23A
T. brucei
T. cruzi G
L. major
B. saltans
Bodo sp.
P. caudatus
Lbp23B
1
141
281
