# Supplementary material for: Genome-wide identification of evolutionarily conserved Small Heat-Shock and eight other proteins bearing α-crystallin domain-like in kinetoplastid protists
Source: PLoS One. 2018 Oct 22;13(10):e0206012. doi: 10.1371/journal.pone.0206012 (PMC6197667; doi:10.1371/journal.pone.0206012)
Supplement: S5 Fig — (PDF) [file pone.0206012.s009.pdf]

ACDp --
Summary of branches under episodic selection (123 were tested, of which 67 required optimizations) :
Node5 p = 0.000158934382335385 (Non-Trypanosome trypanossomatids ancestor)
Node39 p = 0.001392524424110864 (Trypanosoma – Terrestrial clade ancestor)
Node15 p = 0.009012673981737218 (*Strigomomas* ancestor)
Strigomonas culicis p = 0.02485066779951189
Node37 p = 0.03553635467089977 (*Trypanosoma* ancestor)

ATOM69 --
Summary of branches under episodic selection (135 were tested, of which 64 required optimizations) :
Endotrypanum schaudinni p = 0
Leishmania infantum p = 0
Node36 p = 4.254125940406084e-10 (subfamily Leishmaninae ancestor)
Node65 p = 1.669438087770203e-08 (*Strigomomas* ancestor)
Strigomonas galati p = 2.719404295081773e-07
Node57 p = 6.654557798690952e-07 (*Strigomomas/Angomonas* ancestor)
Node73 p = 2.867100081349605e-05 (*Trypanosoma* ancestor)
Node125 p = 9.47043304997397e-05 (Zelonia costaricensis clade)
Strigomonas oncopelti p = 0.000627969690893293
Node29 p = 0.0006547045679319852 (*Crithidia fasciculata* clade)
Node74 p = 0.002747850117420958 (*Trypanosoma* Aquatic clade)
Node58 p = 0.005070714886195871 (Angomonas ancestor)
Node72 p = 0.007579468434232295 (Non-Trypanosome trypanossomatids ancestor)
Node28 p = 0.01181723800526335 (Zelonia/Leishmania/Endotrrypanum)
Node39 p = 0.02442350284057992 (Phytomonas sp. EM1 – gene copies)
Node94 p = 0.02613873219021023 (Trypanosoma grayi clade)
Node53 p = 0.04479682326085255 (*Herpetomonas* ancestor)

DYX1C1 --
Summary of branches under episodic selection (119 were tested, of which 54 required optimizations) :
Node11 p = 5.995204332975845e-14 (*Trypanosoma* ancestor)
Endotrypanum monterogeii p = 2.235081564272434e-10
Node66 p = 1.585307440876704e-09 (*Strigomomas* ancestor)
Phytomonas sp TCC066E p = 2.297754093494309e-06
Node18 p = 0.0001632461131566565 (*Crithidia fasciculata* clade)
Trypanosoma ralphi p = 0.0002022691839162483
Node49 p = 0.0003389629830572449 (*L. infantum/ L. donovani*)
Trypanosoma sp TCC2186 p = 0.001817478320643884
Node17 p = 0.002351794786299877 (subfamily Leishmaninae ancestor)
Strigomonas culicis p = 0.003286528578563264
Node72 p = 0.003830559032013681 (*Herpetomonas* ancestor)
Leishmania panamensis p = 0.02215728280065937
Node71 p = 0.02414245945492999 (*Herpetomonas/Phytomonas* ancestor)

HSP20 --
Summary of branches under episodic selection (119 were tested, of which 35 required optimizations) :
Node99 p = 0.000210433620318029 (*Crithidia fasciculata* clade)

NUDC1 --
Summary of branches under episodic selection (123 were tested, of which 63 required optimizations) :
Node101 p = 1.018052309120776e-11 (*L. infantum/ L. donovani / L. aethiopica*)
Node74 p = 6.962167642399208e-08 (subfamily Leishmaninae ancestor)
Node69 p = 6.525848019744984e-07 (*Strigomomas* ancestor)
Leishmania arabica p = 3.245209608870869e-06
Node109 p = 2.075612154150974e-05 (*L. major* / *L. turanica* / *L. arabica*)
Node44 p = 0.0003476616517361641 (*Trypanosoma* ancestor)
Node61 p = 0.0003606601937355958 (*Herpetomonas* ancestor)
Trypanosoma sp TCC2186 p = 0.0007430180124178287
Node75 p = 0.0009183907065883901 (*Crithidia fasciculata* clade)
Leishmania braziliensis p = 0.0009353995733929477
Node51 p = 0.008493007562787813 (*Herpetomonas/Phytomonas* ancestor)
Node45 p = 0.01569012162976041 (Free-living/parasites)
Crithidia acantocephali p = 0.02029364280919665
Node65 p = 0.02577746829366512 (*Strigomomas/Angomonas* ancestor)
Trypanosoma conorhini p = 0.03656848423389314
Trypanosoma theileri p = 0.03758845825561047

NUDC2 --
Summary of branches under episodic selection (125 were tested, of which 41 required optimizations) :
Node6 p = 0.02950276511807098 (*Trypanosoma* ancestor)

p23A --
Summary of branches under episodic selection (123 were tested, of which 72 required optimizations) :
Endotrypanum monterogeii p = 3.931810229684807e-05
Node57 p = 8.762825332070401e-05 (Angomonas ancestor)
Node66 p = 0.0001090712578475372 (*Crithidia fasciculata* clade)
Node2 p = 0.0002569951173285734 (Trypanosomatidae ancestor)
Node51 p = 0.001426630021823527 (*Strigomomas/Angomonas* ancestor)
Node109 p = 0.00377063781451864 (Phytomonas serpens / Phytomonas sp. TCC066)
Node119 p = 0.004299105677418646 (Paraboso caudatus gene copies)
Node50 p = 0.008067784839998982 (Non-Trypanosome trypanossomatids ancestor)
Leptomonas costaricensis p = 0.008623680068982731
Node60 p = 0.01318684488188809 (*Herpetomonas/Phytomonas/Leishmaninae)*
Herpetomonas wanderleyi p = 0.01427410733392487
Crithidia luciliae thermophila p = 0.01569653833288259
Trypanosoma congolense p = 0.02485519142779236

p23B --
Summary of branches under episodic selection (127 were tested, of which 68 required optimizations) :
Parabodo caudatus p = 4.694022948115162e-13
Node6 p = 2.175415930727453e-07 (*Trypanosoma* ancestor)
Trypanosoma sp TCC2186 p = 0.000159108945360309
Node106 p = 0.0006689297392468152 (*Strigomomas* ancestor)
Node58 p = 0.0008788886711308397 (subfamily Leishmaninae ancestor)
Node47 p = 0.002448725771772797 (T. rangeli / T. conorhini clade ancestor)

Trypanosoma cruzi marinkellei p = 0.00607750448577693
Node103 p = 0.008799236467612381 (*Strigomomas/Angomonas* ancestor)
Node14 p = 0.01636662228345487 (*Trypanosoma* Terrestrial clade ancestor)
Bodo sp p = 0.02287741116838204
Crithidialu ciliae thermophila p = 0.03175189013363627

SGT1 ---
Summary of branches under episodic selection (123 were tested, of which 51 required optimizations) :
Node48 p = 1.199222943171208e-10 (subfamily Leishmaninae ancestor)
Node31 p = 5.807178538042024e-06 (*Trypanosoma* ancestor)
Node100 p = 0.0006507391240253568 (*Herpetomonas* ancestor)
Trypanosoma lewisi p = 0.04100387581892023

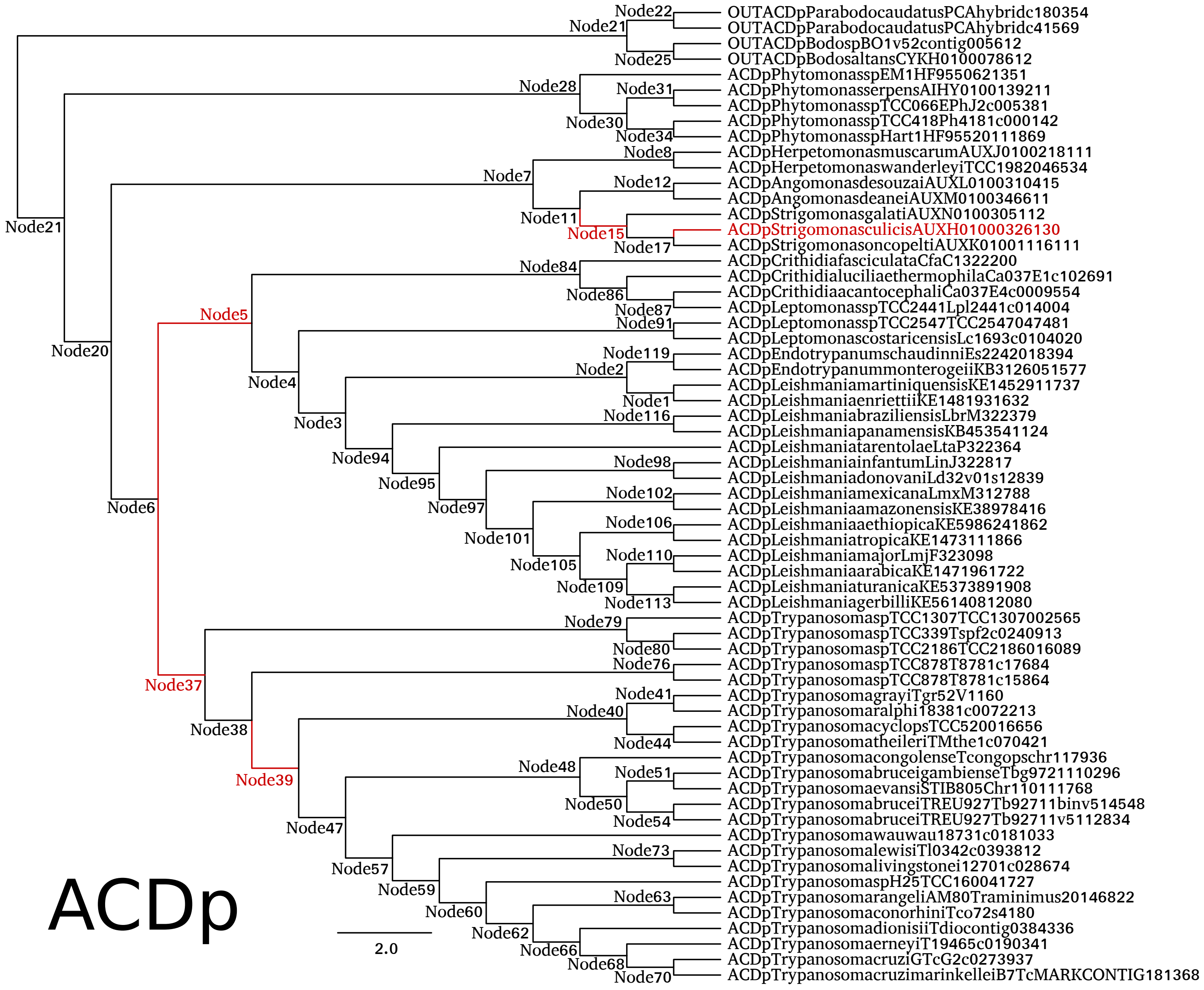
OUTACDpParabodocaudatusPCAhybridc180354
OUTACDpParabodocaudatusPCAhybridc41569
OUTACDpBodospBO1v52contig005612
OUTACDpBodosaltansCYKH0100078612
ACDpPhytomonasspEM1HF9550621351
ACDpPhytomonasserpensAIHY0100139211
ACDpPhytomonasspTCC066EPhJ2c005381
ACDpPhytomonasspTCC418Ph4181c000142
ACDpPhytomonasspHart1HF95520111869
ACDpHerpetomonasmuscarumAUXJ0100218111
ACDpHerpetomonaswanderleyiTCC1982046534
ACDpAngomonasdesouzaiAUXL0100310415
ACDpAngomonasdeaneiAUXM0100346611
ACDpStrigomonasgalatiAUXN0100305112
ACDpStrigomonasculicisAUXH01000326130
ACDpStrigomonasoncopeltiAUXK01001116111
ACDpCrithidiafasciculataCfaC1322200
ACDpCrithidialuciliaethermophilaCa037E1c102691
ACDpCrithidiaacantocephaliCa037E4c0009554
ACDpLeptomonasspTCC2441Lpl2441c014004
ACDpLeptomonasspTCC2547TCC2547047481
ACDpLeptomonascostaricensisLc1693c0104020
ACDpEndotrypanumschaudinniEs2242018394
ACDpEndotrypanummonterogeiiKB3126051577
ACDpLeishmaniamartiniquensisKE1452911737
ACDpLeishmaniaenriettiiKE1481931632
ACDpLeishmaniabraziliensisLbrM322379
ACDpLeishmaniapanamensisKB453541124
ACDpLeishmaniatarentolaeLtaP322364
ACDpLeishmaniainfantumLinJ322817
ACDpLeishmaniadonovaniLd32v01s12839
ACDpLeishmaniamexicanaLmxM312788
ACDpLeishmaniaamazonensisKE38978416
ACDpLeishmaniaaethiopicaKE5986241862
ACDpLeishmaniatropicaKE1473111866
ACDpLeishmaniamajorLmjF323098
ACDpLeishmaniaarabicaKE1471961722
ACDpLeishmaniaturanicaKE5373891908
ACDpLeishmaniagerbilliKE56140812080
ACDpTrypanosomaspTCC1307TCC1307002565
ACDpTrypanosomaspTCC339Tspf2c0240913
ACDpTrypanosomaspTCC2186TCC2186016089
ACDpTrypanosomaspTCC878T8781c17684
ACDpTrypanosomaspTCC878T8781c15864
ACDpTrypanosomagrayiTgr52V1160
ACDpTrypanosomaralphi18381c0072213
ACDpTrypanosomacyclopsTCC520016656
ACDpTrypanosomatheileriTMthe1c070421
ACDpTrypanosomacongolenseTcongopschr117936
ACDpTrypanosomabruceigambienseTbg9721110296
ACDpTrypanosomaevansiSTIB805Chr110111768
ACDpTrypanosomabruceiTREU927Tb92711binv514548
ACDpTrypanosomabruceiTREU927Tb92711v5112834
ACDpTrypanosomawauwau18731c0181033
ACDpTrypanosomalewisiTl0342c0393812
ACDpTrypanosomalivingstonei12701c028674
ACDpTrypanosomaspH25TCC160041727
ACDpTrypanosomarangeliAM80Traminimus20146822
ACDpTrypanosomaconorhiniTco72s4180
ACDpTrypanosomadionisiiTdiocontig0384336
ACDpTrypanosomaerneyiT19465c0190341
ACDpTrypanosomacruziGTcG2c0273937
ACDpTrypanosomacruzimarinkelleiB7TcMARKCONTIG181368
Node22
Node21
Node25
Node28
Node31
Node30
Node34
Node8
Node7
Node12
Node11
Node15
Node17
Node21
Node84
Node86
Node87
Node5
Node91
Node20
Node119
Node2
Node4
Node1
Node116
Node3
Node94
Node98
Node95
Node97
Node102
Node6
Node106
Node101
Node110
Node105
Node109
Node113
Node79
Node80
Node76
Node37
Node41
Node38
Node40
Node44
Node39
Node48
Node51
Node50
Node47
Node54
Node73
Node57
Node59
Node63
Node60
Node62
Node66
Node68
Node70
ACDp
2.0

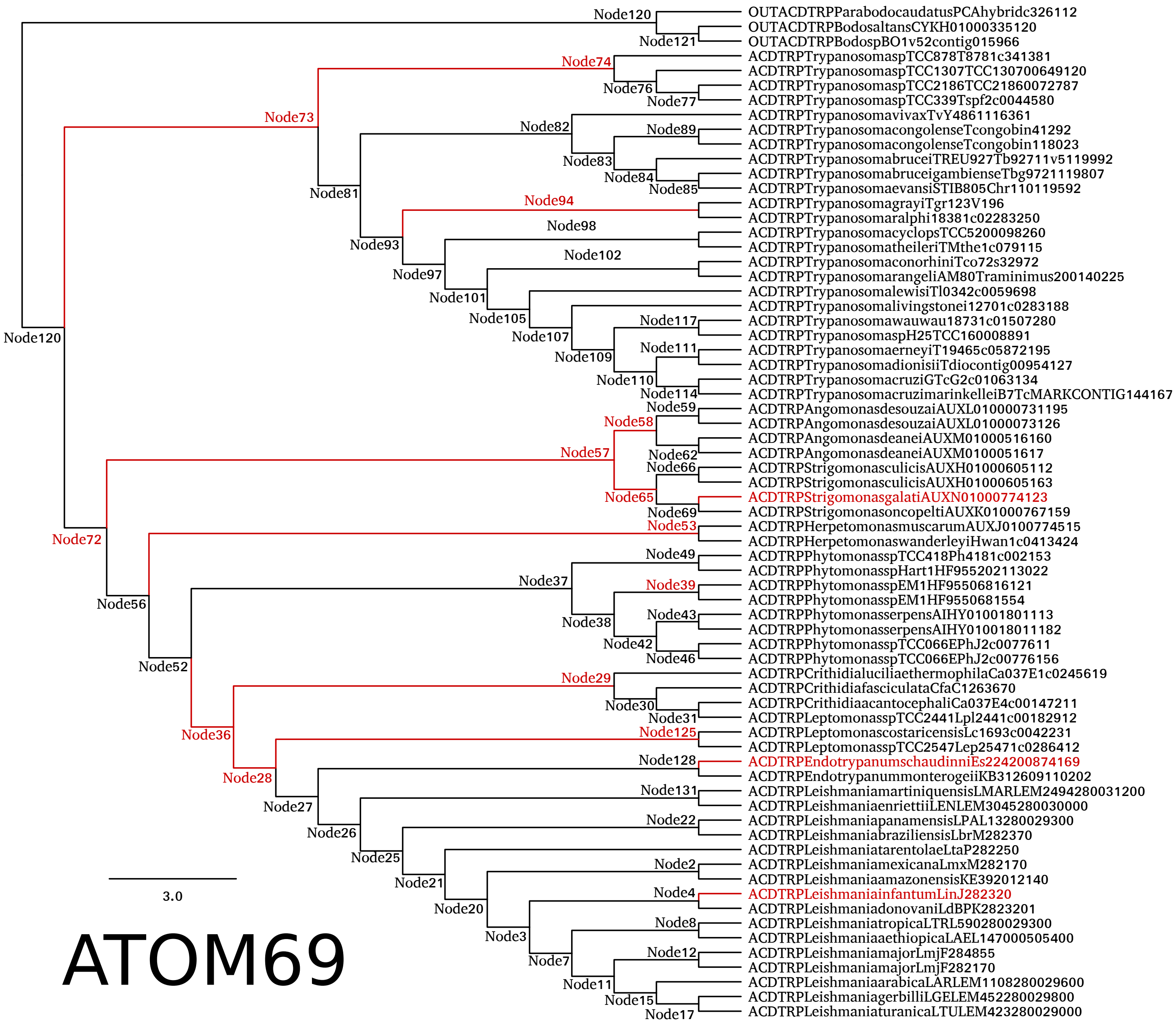

Node120
OUTACDTRPParabodocaudatusPCAhybridc326112
Node121
OUTACDTRPBodosaltansCYKH01000335120
OUTACDTRPBodospBO1v52contig015966
Node74
ACDTRPTrypanosomaspTCC878T8781c341381
Node76
ACDTRPTrypanosomaspTCC1307TCC130700649120
Node77
ACDTRPTrypanosomaspTCC2186TCC21860072787
ACDTRPTrypanosomaspTCC339Tspf2c0044580
Node73
Node82
ACDTRPTrypanosomavivaxTvY4861116361
Node89
ACDTRPTrypanosomacongolenseTcongobin41292
ACDTRPTrypanosomacongolenseTcongobin118023
Node83
ACDTRPTrypanosomabruceiTREU927Tb92711v5119992
Node84
ACDTRPTrypanosomabruceigambienseTbg9721119807
Node85
ACDTRPTrypanosomaevansiSTIB805Chr110119592
Node81
Node94
ACDTRPTrypanosomagrayiTgr123V196
ACDTRPTrypanosomaralphi18381c02283250
Node98
ACDTRPTrypanosomacyclopsTCC5200098260
ACDTRPTrypanosomatheileriTMthe1c079115
Node93
Node102
ACDTRPTrypanosomaconorhiniTco72s32972
ACDTRPTrypanosomarangeliAM80Traminimus200140225
Node97
ACDTRPTrypanosomalewisiTl0342c0059698
Node101
ACDTRPTrypanosomalivingstonei12701c0283188
Node105
Node117
ACDTRPTrypanosomawauwau18731c01507280
Node107
ACDTRPTrypanosomaspH25TCC160008891
Node111
ACDTRPTrypanosomaerneyiT19465c05872195
Node109
ACDTRPTrypanosomadionisiiTdiocontig00954127
Node110
ACDTRPTrypanosomacruziGTcG2c01063134
Node114
ACDTRPTrypanosomacruzimarinkelleiB7TcMARKCONTIG144167
Node59
ACDTRPAngomonasdesouzaiAUXL010000731195
Node58
ACDTRPAngomonasdesouzaiAUXL01000073126
ACDTRPAngomonasdeaneiAUXM01000516160
Node57
Node62
ACDTRPAngomonasdeaneiAUXM0100051617
Node66
ACDTRPStrigomonasculicisAUXH01000605112
ACDTRPStrigomonasculicisAUXH01000605163
Node65
ACDTRPStrigomonasgalatiAUXN01000774123
Node72
Node69
ACDTRPStrigomonasoncopeltiAUXK01000767159
Node53
ACDTRPHerpetomonasmuscarumAUXJ0100774515
ACDTRPHerpetomonaswanderleyiHwan1c0413424
Node49
ACDTRPPhytomonasspTCC418Ph4181c002153
Node37
ACDTRPPhytomonasspHart1HF955202113022
Node56
Node39
ACDTRPPhytomonasspEM1HF95506816121
ACDTRPPhytomonasspEM1HF9550681554
Node43
ACDTRPPhytomonasserpensAIHY01001801113
Node38
ACDTRPPhytomonasserpensAIHY010018011182
Node42
ACDTRPPhytomonasspTCC066EPhJ2c0077611
Node46
ACDTRPPhytomonasspTCC066EPhJ2c00776156
Node52
ACDTRPCrithidialuciliaethermophilaCa037E1c0245619
Node29
ACDTRPCrithidiafasciculataCfaC1263670
Node30
ACDTRPCrithidiaacantocephaliCa037E4c00147211
Node31
ACDTRPLeptomonasspTCC2441Lpl2441c00182912
Node36
Node125
ACDTRPLeptomonascostaricensisLc1693c0042231
ACDTRPLeptomonasspTCC2547Lep25471c0286412
Node128
ACDTRPEndotrypanumschaudinniEs224200874169
Node28
ACDTRPEndotrypanummonterogeiiKB312609110202
Node131
ACDTRPLeishmaniamartiniquensisLMARLEM2494280031200
Node27
ACDTRPLeishmaniaenriettiiLENLEM3045280030000
Node22
ACDTRPLeishmaniapanamensisLPAL13280029300
Node26
ACDTRPLeishmaniabraziliensisLbrM282370
ACDTRPLeishmaniatarentolaeLtaP282250
Node25
Node2
ACDTRPLeishmaniamexicanaLmxM282170
Node21
ACDTRPLeishmaniaamazonensisKE392012140
3.0
Node4
ACDTRPLeishmaniainfantumLinJ282320
Node20
ACDTRPLeishmaniadonovaniLdBPK2823201
Node8
ACDTRPLeishmaniatropicaLTRL590280029300
Node3
ACDTRPLeishmaniaaethiopicaLAEL147000505400
Node12
ACDTRPLeishmaniamajorLmjF284855
Node7
ACDTRPLeishmaniamajorLmjF282170
Node11
ACDTRPLeishmaniaarabicaLARLEM1108280029600
Node15
ACDTRPLeishmaniagerbilliLGELEM452280029800
Node17
ACDTRPLeishmaniaturanicaLTULEM423280029000

ATOM69

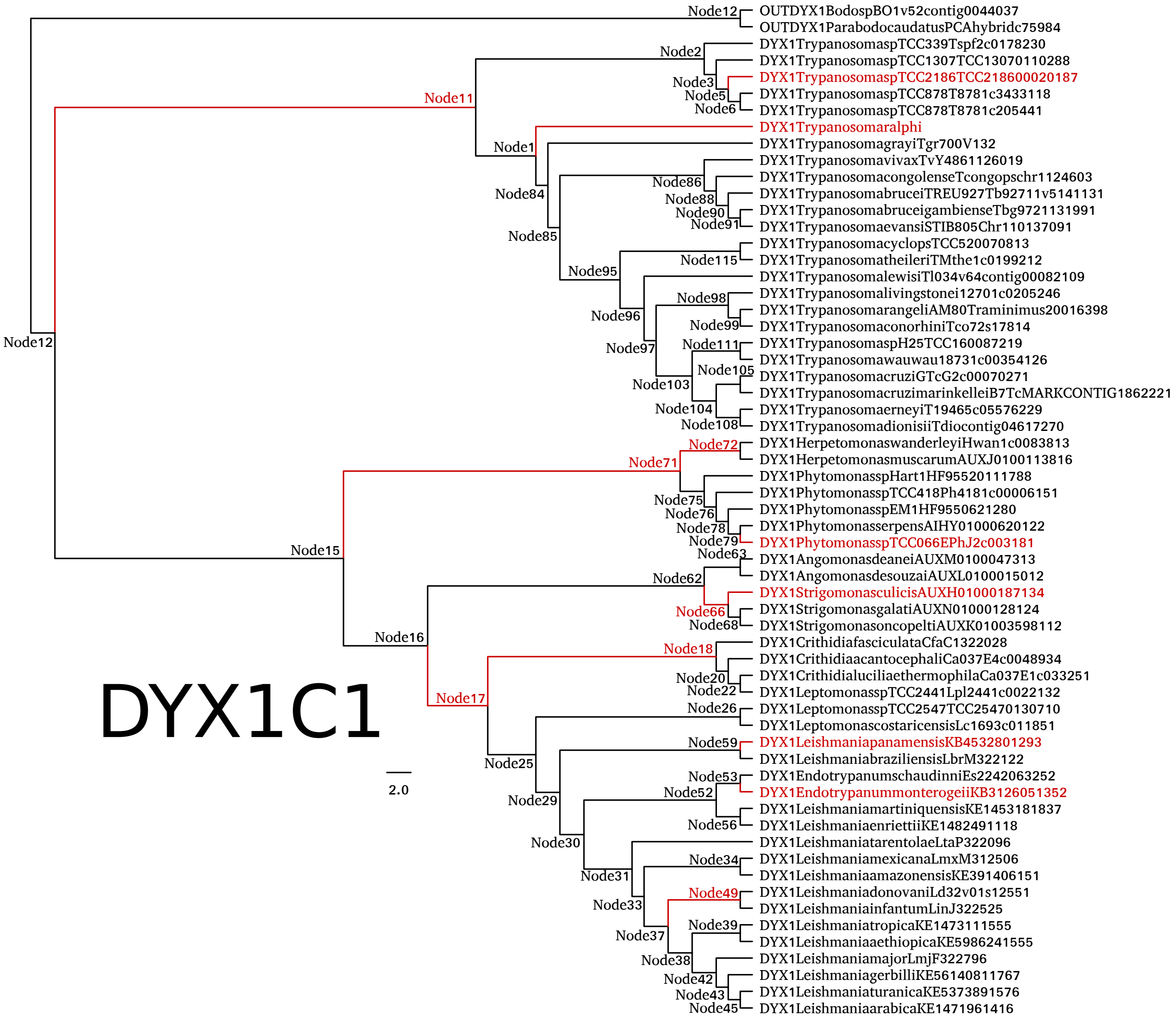
DYX1C1
2.0
Node12
OUTDYX1BodospBO1v52contig0044037
OUTDYX1ParabodocaudatusPCAhybridc75984
Node11
Node2
DYX1TrypanosomaspTCC339Tspf2c0178230
DYX1TrypanosomaspTCC1307TCC13070110288
Node3
DYX1TrypanosomaspTCC2186TCC218600020187
Node5
DYX1TrypanosomaspTCC878T8781c3433118
Node6
DYX1TrypanosomaspTCC878T8781c205441
Node1
DYX1Trypanosomaralphi
Node84
DYX1TrypanosomagrayiTgr700V132
Node85
DYX1TrypanosomavivaxTvY4861126019
Node86
DYX1TrypanosomacongolenseTcongopschr1124603
Node88
DYX1TrypanosomabruceiTREU927Tb92711v5141131
Node90
DYX1TrypanosomabruceigambienseTbg9721131991
Node91
DYX1TrypanosomaevansiSTIB805Chr110137091
Node95
Node115
DYX1TrypanosomacyclopsTCC520070813
DYX1TrypanosomatheileriTMthe1c0199212
Node96
DYX1TrypanosomalewisiTl034v64contig00082109
Node97
Node98
DYX1Trypanosomalivingstonei12701c0205246
Node99
DYX1TrypanosomarangeliAM80Traminimus20016398
DYX1TrypanosomaconorhiniTco72s17814
Node103
Node111
DYX1TrypanosomaspH25TCC160087219
DYX1Trypanosomawauwau18731c00354126
Node104
Node105
DYX1TrypanosomacruziGTcG2c00070271
DYX1TrypanosomacruzimarinkelleiB7TcMARKCONTIG1862221
Node108
DYX1TrypanosomaerneyiT19465c05576229
DYX1TrypanosomadionisiiTdiocontig04617270
Node15
Node71
Node72
DYX1HerpetomonaswanderleyiHwan1c0083813
DYX1HerpetomonasmuscarumAUXJ0100113816
Node75
DYX1PhytomonasspHart1HF95520111788
Node76
DYX1PhytomonasspTCC418Ph4181c00006151
Node78
DYX1PhytomonasspEM1HF9550621280
Node79
DYX1PhytomonasserpensAIHY01000620122
DYX1PhytomonasspTCC066EPhJ2c003181
Node16
Node62
Node63
DYX1AngomonasdeaneiAUXM0100047313
DYX1AngomonasdesouzaiAUXL0100015012
Node66
DYX1StrigomonasculicisAUXH01000187134
Node68
DYX1StrigomonasgalatiAUXN01000128124
DYX1StrigomonasoncopeltiAUXK01003598112
Node17
Node18
DYX1CrithidiafasciculataCfaC1322028
Node20
DYX1CrithidiaacantocephaliCa037E4c0048934
Node22
DYX1CrithidialuciliaethermophilaCa037E1c033251
DYX1LeptomonassspTCC2441Lpl2441c0022132
Node25
Node26
DYX1LeptomonasspTCC2547TCC25470130710
DYX1LeptomonascostaricensisLc1693c011851
Node29
Node59
DYX1LeishmaniapanamensisKB4532801293
DYX1LeishmaniabraziliensisLbrM322122
Node30
Node52
Node53
DYX1EndotrypanumschaudinniEs2242063252
DYX1EndotrypanummonterogeiiKB3126051352
Node56
DYX1LeishmaniamartiniquensisKE1453181837
DYX1LeishmaniaenriettiiKE1482491118
Node31
DYX1LeishmaniatarentolaeLtaP322096
Node33
Node34
DYX1LeishmaniamexicanaLmxM312506
DYX1LeishmaniaamazonensisKE391406151
Node37
Node49
DYX1LeishmaniadonovaniLd32v01s12551
DYX1LeishmaniainfantumLinJ322525
Node38
Node39
DYX1LeishmaniatropicaKE1473111555
DYX1LeishmaniaaethiopicaKE5986241555
Node42
DYX1LeishmaniamajorLmjF322796
Node43
DYX1LeishmaniagerbilliKE56140811767
Node45
DYX1LeishmaniaturanicaKE5373891576
DYX1LeishmaniaarabicaKE1471961416

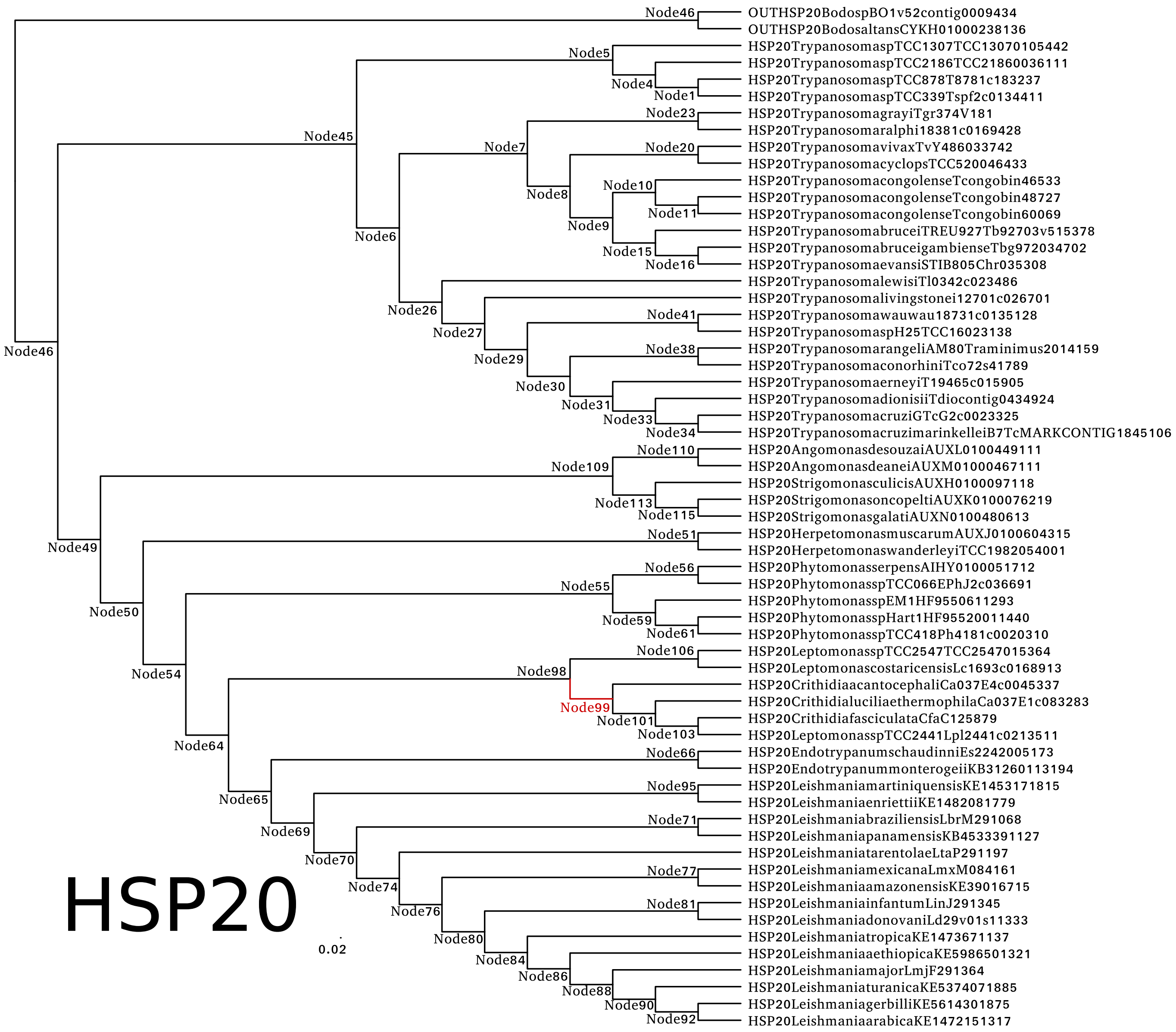
OUTHSP20BodospBO1v52contig0009434
OUTHSP20BodosaltansCYKH01000238136
HSP20TrypanosomaspTCC1307TCC13070105442
HSP20TrypanosomaspTCC2186TCC21860036111
HSP20TrypanosomaspTCC878T8781c183237
HSP20TrypanosomaspTCC339Tspf2c0134411
HSP20TrypanosomagrayiTgr374V181
HSP20Trypanosomaralphi18381c0169428
HSP20TrypanosomavivaxTvY486033742
HSP20TrypanosomacyclopsTCC520046433
HSP20TrypanosomacongolenseTcongobin46533
HSP20TrypanosomacongolenseTcongobin48727
HSP20TrypanosomacongolenseTcongobin60069
HSP20TrypanosomabruceiTREU927Tb92703v515378
HSP20TrypanosomabruceigambienseTbg972034702
HSP20TrypanosomaevansiSTIB805Chr035308
HSP20TrypanosomalewisiTl0342c023486
HSP20Trypanosomalivingstonei12701c026701
HSP20Trypanosomawauwau18731c0135128
HSP20TrypanosomaspH25TCC16023138
HSP20TrypanosomarangeliAM80Traminimus2014159
HSP20TrypanosomaconorhiniTco72s41789
HSP20TrypanosomaerneyiT19465c015905
HSP20TrypanosomadionisiiTdiocontig0434924
HSP20TrypanosomacruziGTcG2c0023325
HSP20TrypanosomacruzimarinkelleiB7TcMARKCONTIG1845106
HSP20AngomonasdesouzaiAUXL0100449111
HSP20AngomonasdeaneiAUXM01000467111
HSP20StrigomonasculicisAUXH0100097118
HSP20StrigomonasoncopeltiAUXK0100076219
HSP20StrigomonasgalatiAUXN0100480613
HSP20HerpetomonasmuscarumAUXJ0100604315
HSP20HerpetomonaswanderleyiTCC1982054001
HSP20PhytomonasserpensAIHY0100051712
HSP20PhytomonassppTCC066EPhJ2c036691
HSP20PhytomonassppEM1HF9550611293
HSP20PhytomonassppHart1HF95520011440
HSP20PhytomonassppTCC418Ph4181c0020310
HSP20LeptomonassppTCC2547TCC2547015364
HSP20LeptomonascostaricensisLc1693c0168913
HSP20CrithidiaacantocephaliCa037E4c0045337
HSP20CrithidialuciliaethermophilaCa037E1c083283
HSP20CrithidiafasciculataCfaC125879
HSP20LeptomonassppTCC2441Lpl2441c0213511
HSP20EndotrypanumschaudinniEs2242005173
HSP20EndotrypanummonterogeiiKB31260113194
HSP20LeishmaniamartiniquensisKE1453171815
HSP20LeishmaniaenriettiiKE1482081779
HSP20LeishmaniabraziliensisLbrM291068
HSP20LeishmaniapanamensisKB4533391127
HSP20LeishmaniatarentolaeLtaP291197
HSP20LeishmaniamexicanaLmxM084161
HSP20LeishmaniaamazonensisKE39016715
HSP20LeishmaniainfantumLinJ291345
HSP20Leishmaniadonovanild29v01s11333
HSP20LeishmaniatropicaKE1473671137
HSP20LeishmaniaaethiopicaKE5986501321
HSP20LeishmaniamajorLmjF291364
HSP20LeishmaniaturanicaKE5374071885
HSP20LeishmaniagerbilliKE5614301875
HSP20LeishmaniaarabicaKE1472151317
HSP20
0.02

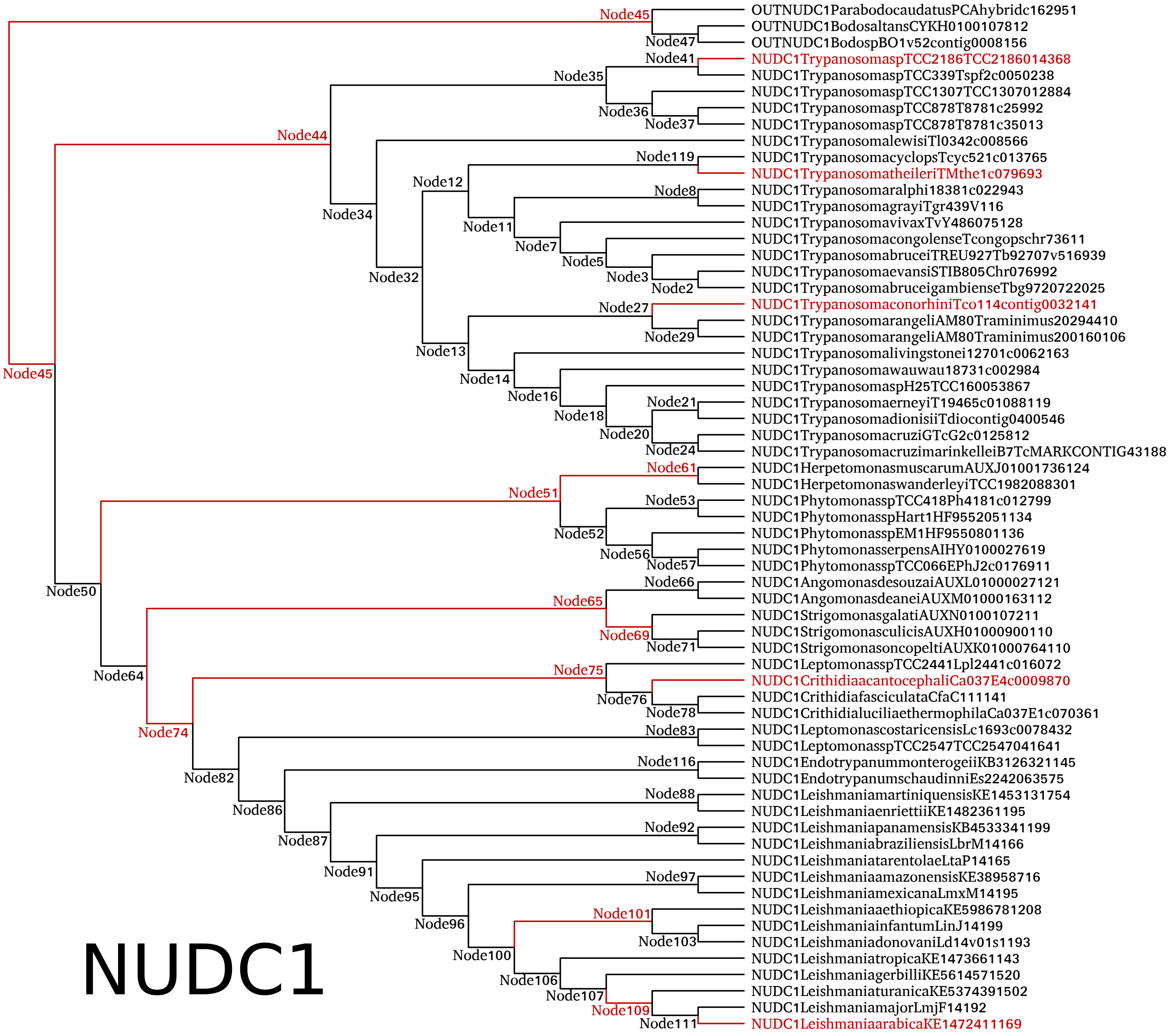
NUDC1
Node45
Node45
Node47
Node41
Node35
Node36
Node37
Node44
Node34
Node119
Node12
Node8
Node11
Node7
Node5
Node3
Node2
Node32
Node27
Node29
Node13
Node14
Node16
Node21
Node18
Node20
Node24
Node61
Node51
Node53
Node52
Node56
Node57
Node50
Node66
Node65
Node69
Node71
Node64
Node75
Node76
Node78
Node74
Node83
Node82
Node116
Node86
Node88
Node87
Node92
Node91
Node95
Node97
Node96
Node101
Node103
Node100
Node106
Node107
Node109
Node111
OUTNUDC1ParabodocaudatusPCAhybridc162951
OUTNUDC1BodosaltansCYKH0100107812
OUTNUDC1BodospBO1v52contig0008156
NUDC1TrypanosomaspTCC2186TCC2186014368
NUDC1TrypanosomaspTCC339Tspf2c0050238
NUDC1TrypanosomaspTCC1307TCC1307012884
NUDC1TrypanosomaspTCC878T8781c25992
NUDC1TrypanosomaspTCC878T8781c35013
NUDC1TrypanosomalewisiTl0342c008566
NUDC1TrypanosomacyclopsTcyc521c013765
NUDC1TrypanosomatheileriTMthe1c079693
NUDC1Trypanosomaralphi18381c022943
NUDC1TrypanosomagrayiTgr439V116
NUDC1TrypanosomavivaxTvY486075128
NUDC1TrypanosomacongolenseTcongopschr73611
NUDC1TrypanosomabruceiTREU927Tb92707v516939
NUDC1TrypanosomaevansiSTIB805Chr076992
NUDC1TrypanosomabruceigambienseTbg9720722025
NUDC1TrypanosomaconorhiniTco114contig0032141
NUDC1TrypanosomarangeliAM80Traminimus20294410
NUDC1TrypanosomarangeliAM80Traminimus200160106
NUDC1Trypanosomalivingstonei12701c0062163
NUDC1Trypanosomawauwau18731c002984
NUDC1TrypanosomaspH25TCC160053867
NUDC1TrypanosomaerneyiT19465c01088119
NUDC1TrypanosomadionisiiTdiocontig0400546
NUDC1TrypanosomacruziGTcG2c0125812
NUDC1TrypanosomacruzimarinkelleiB7TcMARKCONTIG43188
NUDC1HerpetomonasmuscarumAUXJ01001736124
NUDC1HerpetomonaswanderleyiTCC1982088301
NUDC1PhytomonasspTCC418Ph4181c012799
NUDC1PhytomonasspHart1HF9552051134
NUDC1PhytomonasspEM1HF9550801136
NUDC1PhytomonasserpensAIHY0100027619
NUDC1PhytomonasspTCC066EPhJ2c0176911
NUDC1AngomonasdesouzaiAUXL01000027121
NUDC1AngomonasdeaneiAUXM01000163112
NUDC1StrigomonasgalatiAUXN0100107211
NUDC1StrigomonasculicisAUXH01000900110
NUDC1StrigomonasoncopeltiAUXK01000764110
NUDC1LeptomonasspTCC2441Lpl2441c016072
NUDC1CrithidiaacantocephaliCa037E4c0009870
NUDC1CrithidiafasciculataCfaC111141
NUDC1CrithidialuciliaethermophilaCa037E1c070361
NUDC1LeptomonascostaricensisLc1693c0078432
NUDC1LeptomonasspTCC2547TCC2547041641
NUDC1EndotrypanummonterogeiiKB3126321145
NUDC1EndotrypanumschaudinniEs2242063575
NUDC1LeishmaniamartiniquensisKE1453131754
NUDC1LeishmaniaenriettiiKE1482361195
NUDC1LeishmaniapanamensisKB4533341199
NUDC1LeishmaniabraziliensisLbrM14166
NUDC1LeishmaniatarentolaeLtaP14165
NUDC1LeishmaniaamazonensisKE38958716
NUDC1LeishmaniamexicanaLmxM14195
NUDC1LeishmaniaaethiopicaKE5986781208
NUDC1LeishmaniainfantumLinJ14199
NUDC1LeishmaniadonovaniLd14v01s1193
NUDC1LeishmaniatropicaKE1473661143
NUDC1LeishmaniagerbilliKE5614571520
NUDC1LeishmaniaturanicaKE5374391502
NUDC1LeishmaniamajorLmjF14192
NUDC1LeishmaniaarabicaKE1472411169

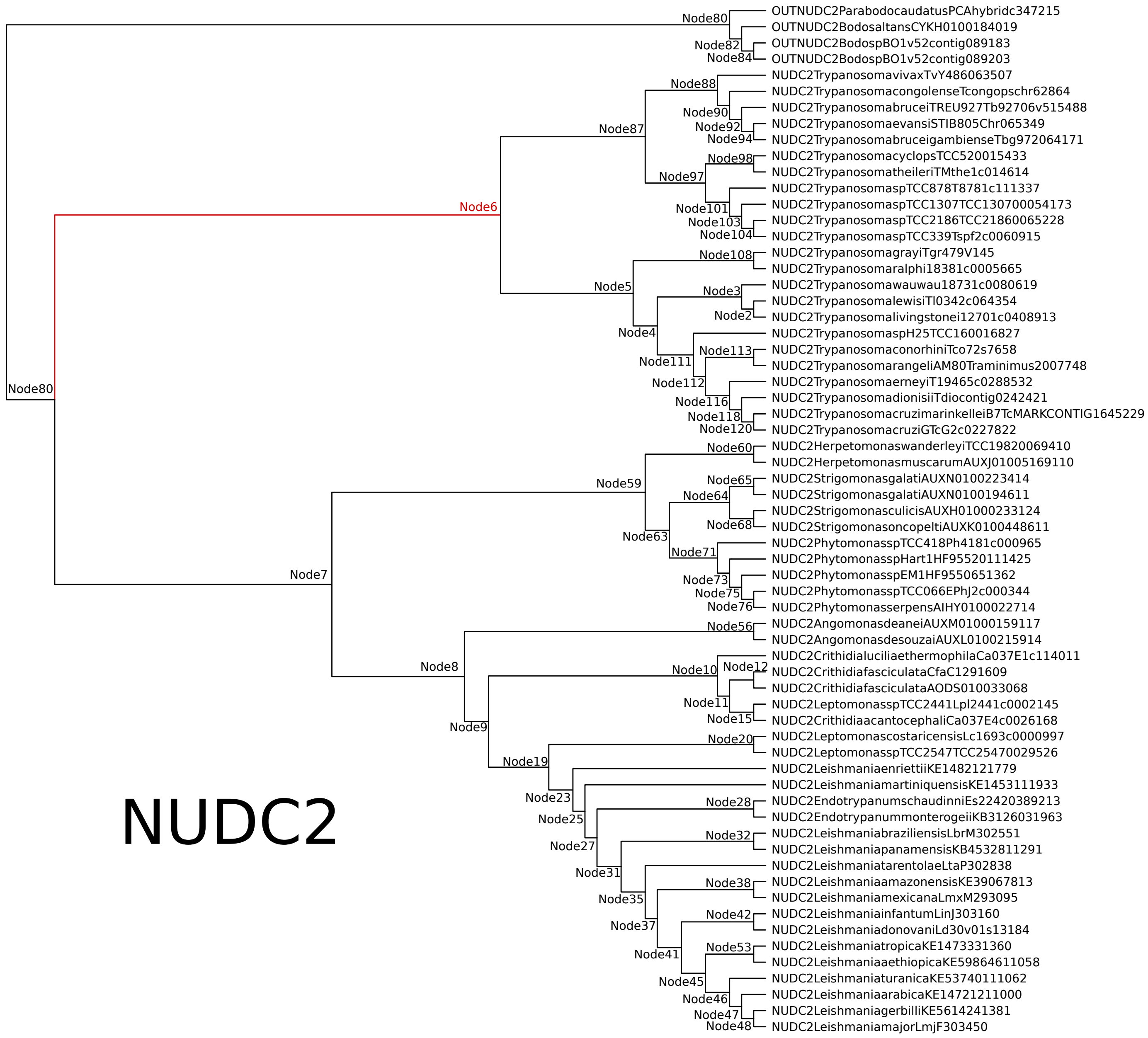
NUDC2
OUTNUDC2ParabodocaudatusPCAhybridc347215
OUTNUDC2BodosaltansCYKH0100184019
OUTNUDC2BodospBO1v52contig089183
OUTNUDC2BodospBO1v52contig089203
NUDC2TrypanosomavivaxTvY486063507
NUDC2TrypanosomacongolenseTcongopschr62864
NUDC2TrypanosomabruceiTREU927Tb92706v515488
NUDC2TrypanosomaevansiSTIB805Chr065349
NUDC2TrypanosomabruceigambienseTbg972064171
NUDC2TrypanosomacyclopsTCC520015433
NUDC2TrypanosomatheileriTmthe1c014614
NUDC2TrypanosomaspTCC878T8781c111337
NUDC2TrypanosomaspTCC1307TCC130700054173
NUDC2TrypanosomaspTCC2186TCC21860065228
NUDC2TrypanosomaspTCC339Tspf2c0060915
NUDC2TrypanosomagrayiTgr479V145
NUDC2Trypanosomaralphi18381c0005665
NUDC2Trypanosomawauwau18731c0080619
NUDC2TrypanosomalewisiTl0342c064354
NUDC2Trypanosomalivingstonei12701c0408913
NUDC2TrypanosomaspH25TCC160016827
NUDC2TrypanosomaconorhiniTco72s7658
NUDC2TrypanosomarangeliAM80Traminimus2007748
NUDC2TrypanosomaerneyiT19465c0288532
NUDC2TrypanosomadionisiiTdiocontig0242421
NUDC2TrypanosomacruzimarinkelleiB7TcMARKCONTIG1645229
NUDC2TrypanosomacruziGTcG2c0227822
NUDC2HerpetomonaswanderleyiTCC19820069410
NUDC2HerpetomonasmuscarumAUXJ01005169110
NUDC2StrigomonasgalatiAUXN0100223414
NUDC2StrigomonasgalatiAUXN0100194611
NUDC2StrigomonasculicisAUXH01000233124
NUDC2StrigomonasoncopeltiAUXK0100448611
NUDC2PhytomonasspTCC418Ph4181c000965
NUDC2PhytomonasspHart1HF95520111425
NUDC2PhytomonasspEM1HF9550651362
NUDC2PhytomonasspTCC066EPhJ2c000344
NUDC2PhytomonasserpensAIHY0100022714
NUDC2AngomonasdeaneiAUXM01000159117
NUDC2AngomonasdesouzaiAUXL0100215914
NUDC2CrithidialuciliaethermophilaCa037E1c114011
NUDC2CrithidiafasciculataCfaC1291609
NUDC2CrithidiafasciculataAODS010033068
NUDC2LeptomonasspTCC2441Lpl2441c0002145
NUDC2CrithidiaacantocephaliCa037E4c0026168
NUDC2LeptomonascostaricensisLc1693c0000997
NUDC2LeptomonasspTCC2547TCC25470029526
NUDC2LeishmaniaenriettiiKE1482121779
NUDC2LeishmaniamartiniquensisKE1453111933
NUDC2EndotrypanumschaudinniEs22420389213
NUDC2EndotrypanummonterogeiiKB3126031963
NUDC2LeishmaniabraziliensisLbrM302551
NUDC2LeishmaniapanamensisKB4532811291
NUDC2LeishmaniatarentolaeLtaP302838
NUDC2LeishmaniaamazonensisKE39067813
NUDC2LeishmaniamexicanaLmxM293095
NUDC2LeishmaniainfantumLinJ303160
NUDC2LeishmaniadonovaniLd30v01s13184
NUDC2LeishmaniatropicaKE1473331360
NUDC2LeishmaniaaethiopicaKE59864611058
NUDC2LeishmaniaturanicaKE53740111062
NUDC2LeishmaniaarabicaKE14721211000
NUDC2LeishmaniagerbilliKE5614241381
NUDC2LeishmaniamajorLmjF303450

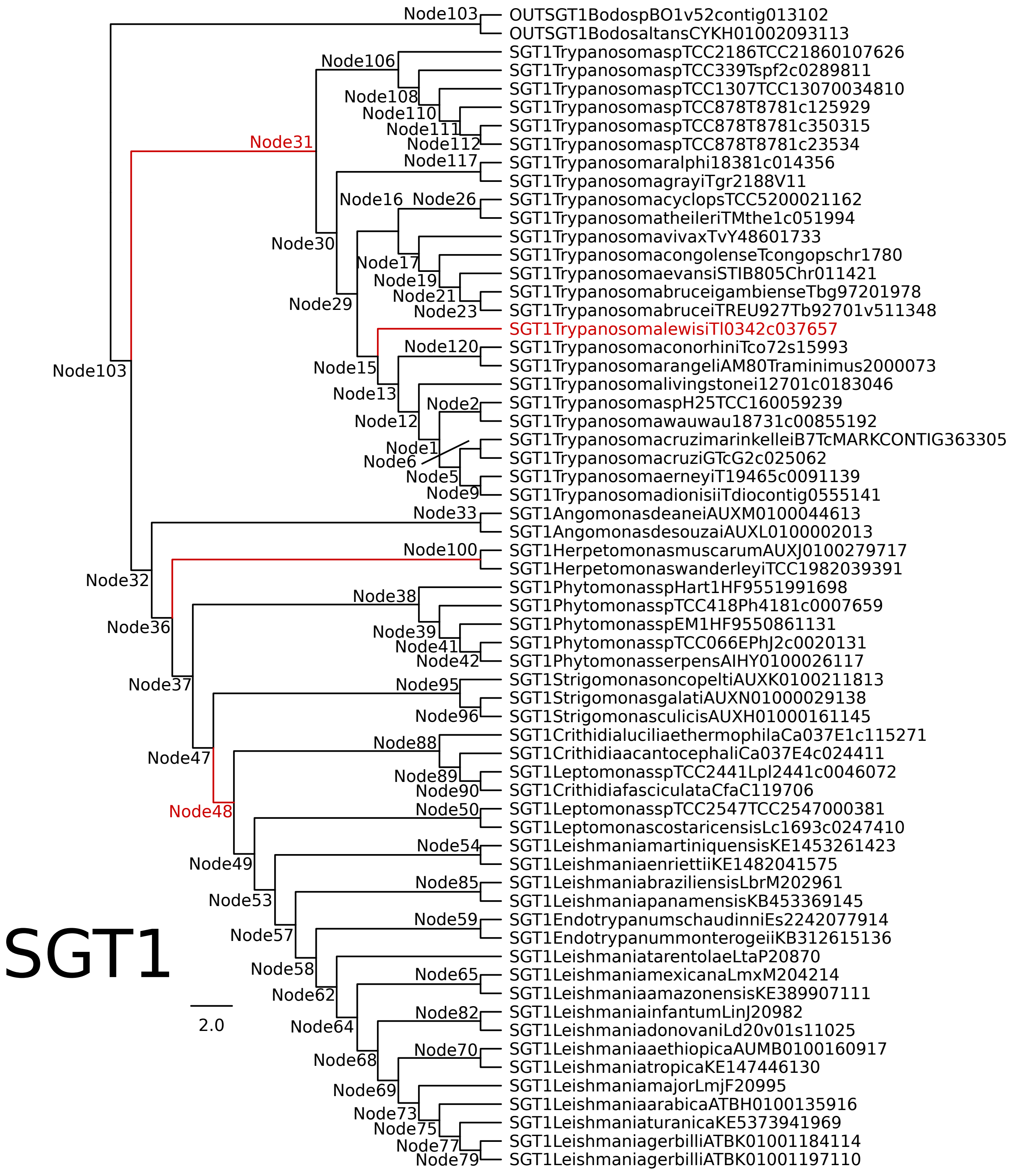
Node103
OUTSGT1BodospBO1v52contig013102
OUTSGT1BodosaltansCYKH01002093113
Node106
SGT1TrypanosomaspTCC2186TCC21860107626
SGT1TrypanosomaspTCC339Tspf2c0289811
Node108
SGT1TrypanosomaspTCC1307TCC13070034810
Node110
SGT1TrypanosomaspTCC878T8781c125929
Node111
SGT1TrypanosomaspTCC878T8781c350315
Node112
SGT1TrypanosomaspTCC878T8781c23534
Node31
Node117
SGT1Trypanosomaralphi18381c014356
SGT1TrypanosomagrayiTgr2188V11
Node16
Node26
SGT1TrypanosomacyclopsTCC5200021162
SGT1TrypanosomatheileriTMthe1c051994
Node30
SGT1TrypanosomavivaxTvY48601733
Node17
SGT1TrypanosomacongolenseTcongopschr1780
Node19
SGT1TrypanosomaevansiSTIB805Chr011421
Node21
SGT1TrypanosomabruceigambienseTbg97201978
Node29
Node23
SGT1TrypanosomabruceiTREU927Tb92701v511348
SGT1TrypanosomalewisiTl0342c037657
Node120
SGT1TrypanosomaconorhiniTco72s15993
Node103
Node15
SGT1TrypanosomarangeliAM80Traminimus2000073
Node13
SGT1Trypanosomalivingstonei12701c0183046
Node2
SGT1TrypanosomaspH25TCC160059239
Node12
SGT1Trypanosomawauwau18731c00855192
Node1
SGT1TrypanosomacruzimarinkelleiB7TcMARKCONTIG363305
Node6
SGT1TrypanosomacruziGTcG2c025062
Node5
SGT1TrypanosomaerneyiT19465c0091139
Node9
SGT1TrypanosomadionisiiTdiocontig0555141
Node33
SGT1AngomonasdeaneiAUXM0100044613
SGT1AngomonasdesouzaiAUXL0100002013
Node100
SGT1HerpetomonasmuscarumAUXJ0100279717
Node32
SGT1HerpetomonaswanderleyiTCC1982039391
Node38
SGT1PhytomonasspHart1HF9551991698
Node36
SGT1PhytomonasspTCC418Ph4181c0007659
Node39
SGT1PhytomonasspEM1HF9550861131
Node41
SGT1PhytomonasspTCC066EPhJ2c0020131
Node42
SGT1PhytomonasserpensAIHY0100026117
Node37
Node95
SGT1StrigomonasoncopeltiAUXK0100211813
SGT1StrigomonasgalatiAUXN01000029138
Node96
SGT1StrigomonasculicisAUXH01000161145
Node88
SGT1CrithidialuciliaethermophilaCa037E1c115271
Node47
SGT1CrithidiaacantocephaliCa037E4c024411
Node89
SGT1LeptomonasspTCC2441Lpl2441c0046072
Node90
SGT1CrithidiafasciculataCfaC119706
Node48
Node50
SGT1LeptomonasspTCC2547TCC2547000381
SGT1LeptomonascostaricensisLc1693c0247410
Node54
SGT1LeishmaniamartiniquensisKE1453261423
Node49
SGT1LeishmaniaenriettiiKE1482041575
Node85
SGT1LeishmaniabraziliensisLbrM202961
Node53
SGT1LeishmaniapanamensisKB453369145
Node59
SGT1EndotrypanumschaudinniEs2242077914
Node57
SGT1EndotrypanummonterogeiiKB312615136
SGT1LeishmaniatarentolaeLtaP20870
Node58
Node65
SGT1LeishmaniamexicanaLmxM204214
Node62
SGT1LeishmaniaamazonensisKE389907111
Node82
SGT1LeishmaniainfantumLinJ20982
Node64
SGT1LeishmaniadonovaniLd20v01s11025
Node70
SGT1LeishmaniaaethiopicaAUMB0100160917
Node68
SGT1LeishmaniatropicaKE147446130
Node69
SGT1LeishmaniamajorLmjF20995
Node73
SGT1LeishmaniaarabicaATBH0100135916
Node75
SGT1LeishmaniaturanicaKE5373941969
Node77
SGT1LeishmaniagerbilliATBK01001184114
Node79
SGT1LeishmaniagerbilliATBK01001197110
SGT1
2.0

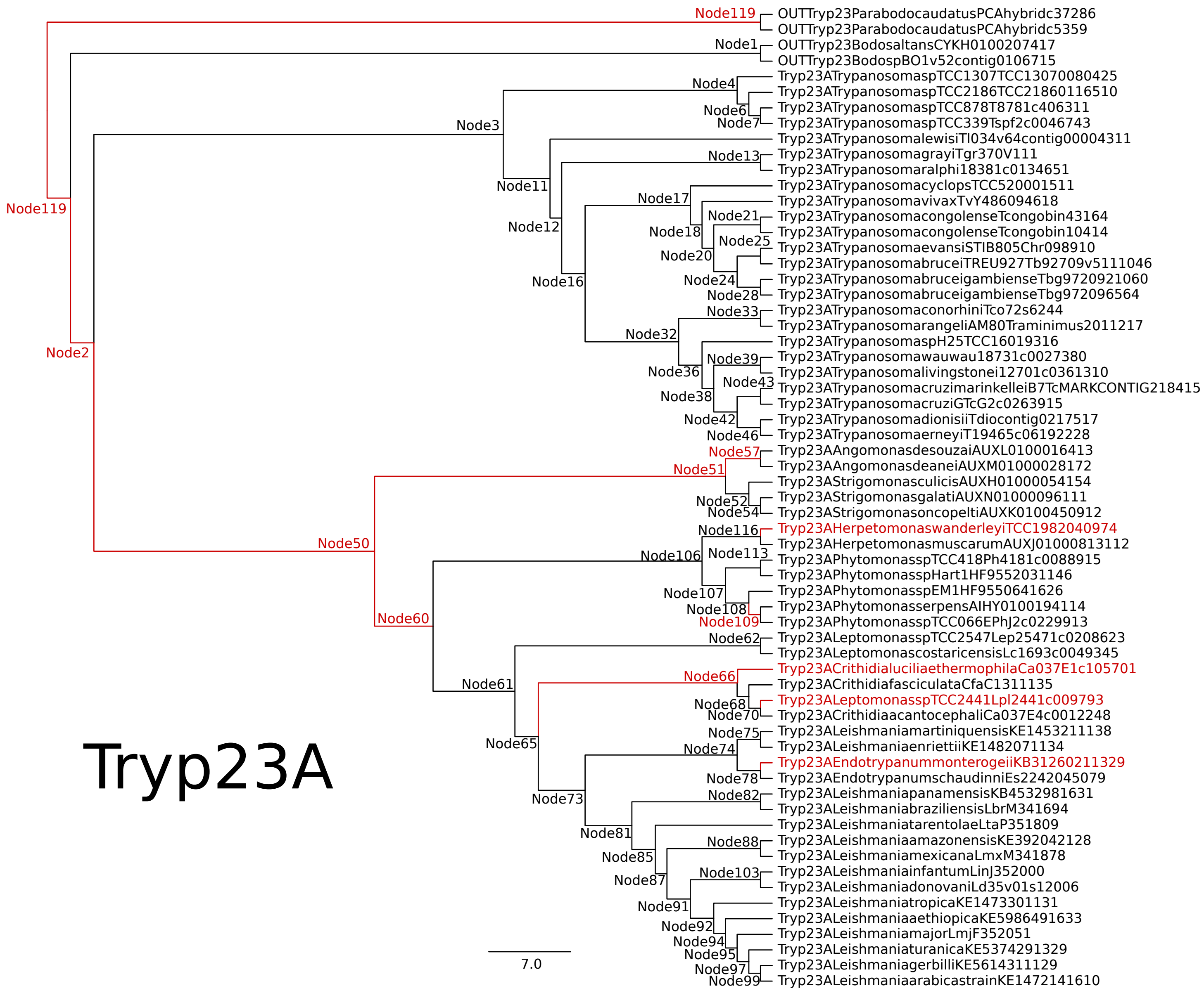

Tryp23A
OUTTryp23ParabodocaudatusPCAhybridc37286
OUTTryp23ParabodocaudatusPCAhybridc5359
OUTTryp23BodosaltansCYKH0100207417
OUTTryp23BodospBO1v52contig0106715
Tryp23ATrypanosomaspTCC1307TCC13070080425
Tryp23ATrypanosomaspTCC2186TCC21860116510
Tryp23ATrypanosomaspTCC878T8781c406311
Tryp23ATrypanosomaspTCC339Tspf2c0046743
Tryp23ATrypanosomalewisiTl034v64contig00004311
Tryp23ATrypanosomagrayiTgr370V111
Tryp23ATrypanosomaralphi18381c0134651
Tryp23ATrypanosomacyclopsTCC520001511
Tryp23ATrypanosomavivaxTvY486094618
Tryp23ATrypanosomacongolenseTcongobin43164
Tryp23ATrypanosomacongolenseTcongobin10414
Tryp23ATrypanosomaevansiSTIB805Chr098910
Tryp23ATrypanosomabruceiTREU927Tb92709v5111046
Tryp23ATrypanosomabruceigambienseTbg9720921060
Tryp23ATrypanosomabruceigambienseTbg972096564
Tryp23ATrypanosomaconorhiniTco72s6244
Tryp23ATrypanosomarangeliAM80Traminimus2011217
Tryp23ATrypanosomaspH25TCC16019316
Tryp23ATrypanosomawauwau18731c0027380
Tryp23ATrypanosomalivingstonei12701c0361310
Tryp23ATrypanosomacruzimarinkelleiB7TcMARKCONTIG218415
Tryp23ATrypanosomacruziGTcG2c0263915
Tryp23ATrypanosomadionisiiTdiocontig0217517
Tryp23ATrypanosomaerneyiT19465c06192228
Tryp23AAngomonasdesouzaiAUXL0100016413
Tryp23AAngomonasdeaneiAUXM01000028172
Tryp23AStrigomonasculicisAUXH01000054154
Tryp23AStrigomonasgalatiAUXN01000096111
Tryp23AStrigomonasoncopeltiAUXK0100450912
Tryp23AHerpetomonaswanderleyiTCC1982040974
Tryp23AHerpetomonasmuscarumAUXJ01000813112
Tryp23APhytomonasspTCC418Ph4181c0088915
Tryp23APhytomonasspHart1HF9552031146
Tryp23APhytomonasspEM1HF9550641626
Tryp23APhytomonasserpensAIHY0100194114
Tryp23APhytomonasspTCC066EPhJ2c0229913
Tryp23ALeptomonasspTCC2547Lep25471c0208623
Tryp23ALeptomonascostaricensisLc1693c0049345
Tryp23ACrithidialuciliaethermophilaCa037E1c105701
Tryp23ACrithidiafasciculataCfaC1311135
Tryp23ALeptomonasspTCC2441Lpl2441c009793
Tryp23ACrithidiaacantocephaliCa037E4c0012248
Tryp23ALeishmaniamartiniquensisKE1453211138
Tryp23ALeishmaniaenriettiiKE1482071134
Tryp23AEndotrypanummonterogeiiKB31260211329
Tryp23AEndotrypanumschaudinniEs2242045079
Tryp23ALeishmaniapanamensisKB4532981631
Tryp23ALeishmaniabraziliensisLbrM341694
Tryp23ALeishmaniatarentolaeLtaP351809
Tryp23ALeishmaniaamazonensisKE392042128
Tryp23ALeishmaniamexicanaLmxM341878
Tryp23ALeishmaniainfantumLinJ352000
Tryp23ALeishmaniadonovaniLd35v01s12006
Tryp23ALeishmaniatropicaKE1473301131
Tryp23ALeishmaniaaethiopicaKE5986491633
Tryp23ALeishmaniamajorLmjF352051
Tryp23ALeishmaniaturanicaKE5374291329
Tryp23ALeishmaniagerbilliKE5614311129
Tryp23ALeishmaniaarabicastrainKE1472141610
Node119
Node1
Node2
Node3
Node4
Node6
Node7
Node11
Node12
Node13
Node16
Node17
Node18
Node20
Node21
Node24
Node25
Node28
Node32
Node33
Node36
Node38
Node39
Node42
Node43
Node46
Node50
Node51
Node52
Node54
Node57
Node60
Node61
Node62
Node65
Node66
Node68
Node70
Node73
Node74
Node75
Node78
Node81
Node82
Node85
Node87
Node88
Node91
Node92
Node94
Node95
Node97
Node99
Node103
Node106
Node107
Node108
Node109
Node113
Node116
7.0

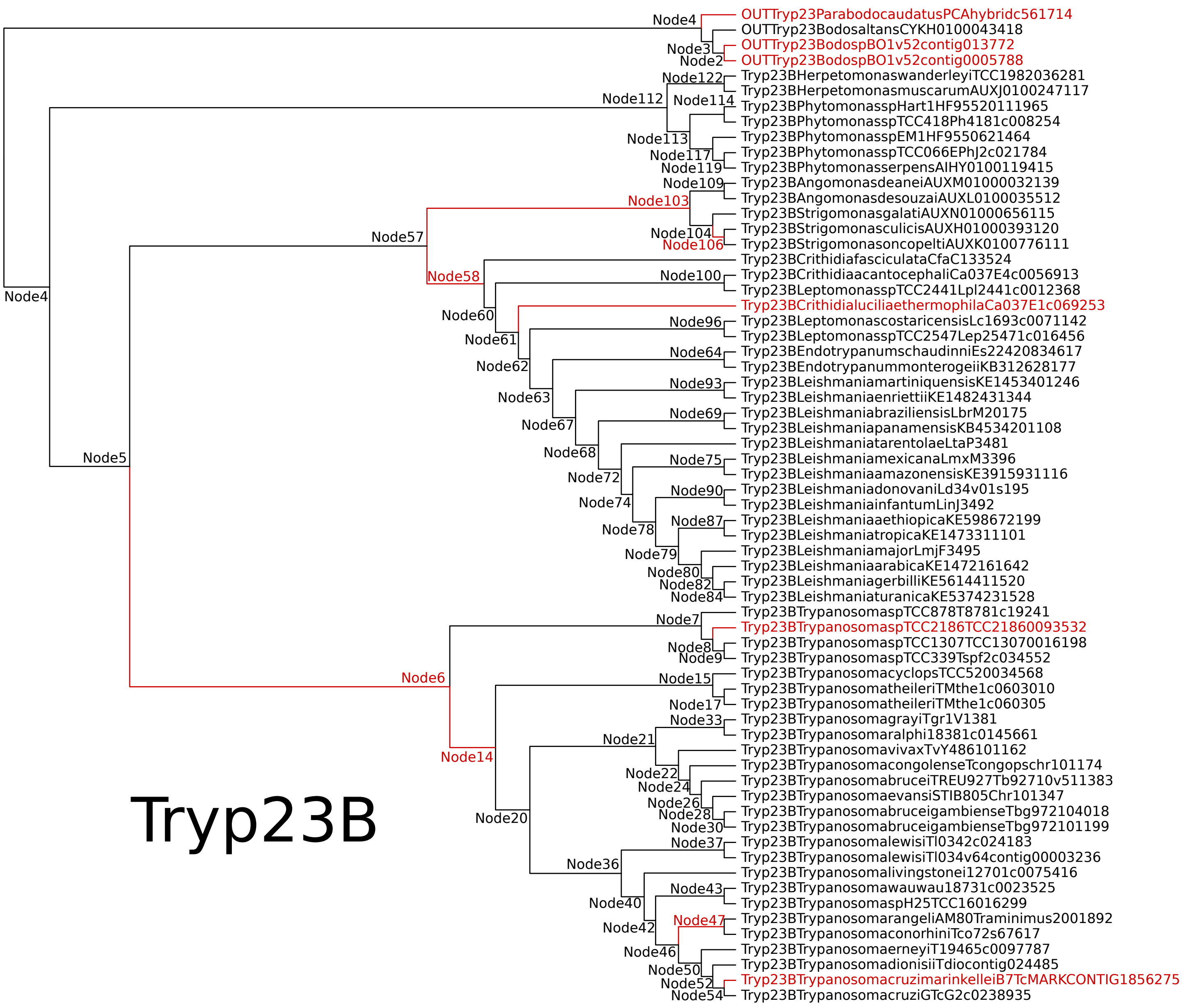
Tryp23B
Node4
Node3
Node2
OUTTryp23ParabodocaudatusPCAhybridc561714
OUTTryp23BodosaltansCYKH0100043418
OUTTryp23BodospBO1v52contig013772
OUTTryp23BodospBO1v52contig0005788
Node112
Node122
Node114
Node113
Node117
Node119
Tryp23BHerpetomonaswanderleyiTCC1982036281
Tryp23BHerpetomonasmuscarumAUXJ0100247117
Tryp23BPhytomonasspHart1HF95520111965
Tryp23BPhytomonasspTCC418Ph4181c008254
Tryp23BPhytomonasspEM1HF9550621464
Tryp23BPhytomonasspTCC066EPhJ2c021784
Tryp23BPhytomonasserpensAIHY0100119415
Node5
Node57
Node103
Node109
Node104
Node106
Tryp23BAngomonasdeaneiAUXM01000032139
Tryp23BAngomonasdesouzaiAUXL0100035512
Tryp23BStrigomonasgalatiAUXN01000656115
Tryp23BStrigomonasculicisAUXH01000393120
Tryp23BStrigomonasoncopeltiAUXK0100776111
Node58
Node60
Node61
Node62
Node63
Node67
Node68
Node72
Node74
Node78
Node79
Node80
Node82
Node84
Node100
Node96
Node64
Node93
Node69
Node75
Node90
Node87
Tryp23BCrithidiafasciculataCfaC133524
Tryp23BCrithidiaacantocephaliCa037E4c0056913
Tryp23BLeptomonasspTCC2441Lpl2441c0012368
Tryp23BCrithidialuciliaethermophilaCa037E1c069253
Tryp23BLeptomonascostaricensisLc1693c0071142
Tryp23BLeptomonasspTCC2547Lep25471c016456
Tryp23BEndotrypanumschaudinniEs22420834617
Tryp23BEndotrypanummonterogeiiKB312628177
Tryp23BLeishmaniamartiniquensisKE1453401246
Tryp23BLeishmaniaenriettiiKE1482431344
Tryp23BLeishmaniabraziliensisLbrM20175
Tryp23BLeishmaniapanamensisKB4534201108
Tryp23BLeishmaniatarentolaeLtaP3481
Tryp23BLeishmaniamexicanaLmxM3396
Tryp23BLeishmaniaamazonensisKE3915931116
Tryp23BLeishmaniadonovaniLd34v01s195
Tryp23BLeishmaniainfantumLinJ3492
Tryp23BLeishmaniaaethiopicaKE598672199
Tryp23BLeishmaniatropicaKE1473311101
Tryp23BLeishmaniamajorLmjF3495
Tryp23BLeishmaniaarabicaKE1472161642
Tryp23BLeishmaniagerbilliKE5614411520
Tryp23BLeishmaniaturanicaKE5374231528
Node6
Node7
Node8
Node9
Tryp23BTrypanosomaspTCC878T8781c19241
Tryp23BTrypanosomaspTCC2186TCC21860093532
Tryp23BTrypanosomaspTCC1307TCC13070016198
Tryp23BTrypanosomaspTCC339Tspf2c034552
Node14
Node15
Node17
Tryp23BTrypanosomacyclopsTCC520034568
Tryp23BTrypanosomatheileriTMthe1c0603010
Tryp23BTrypanosomatheileriTMthe1c060305
Node20
Node21
Node33
Node22
Node24
Node26
Node28
Node30
Tryp23BTrypanosomagrayiTgr1V1381
Tryp23BTrypanosomaralphi18381c0145661
Tryp23BTrypanosomavivaxTvY486101162
Tryp23BTrypanosomacongolenseTcongopschr101174
Tryp23BTrypanosomabruceiTREU927Tb92710v511383
Tryp23BTrypanosomaevansiSTIB805Chr101347
Tryp23BTrypanosomabruceigambienseTbg972104018
Tryp23BTrypanosomabruceigambienseTbg972101199
Node36
Node37
Node40
Node42
Node43
Node46
Node47
Node50
Node52
Node54
Tryp23BTrypanosomalewisiTl0342c024183
Tryp23BTrypanosomalewisiTl034v64contig00003236
Tryp23BTrypanosomalivingstonei12701c0075416
Tryp23BTrypanosomawauwau18731c0023525
Tryp23BTrypanosomaspH25TCC16016299
Tryp23BTrypanosomarangeliAM80Traminimus2001892
Tryp23BTrypanosomaconorhiniTco72s67617
Tryp23BTrypanosomaerneyiT19465c0097787
Tryp23BTrypanosomadionisiiTdiocontig024485
Tryp23BTrypanosomacruzimarinkelleiB7TcMARKCONTIG1856275
Tryp23BTrypanosomacruziGTcG2c0238935
